# Construal and Contact in Person–Situation Relations: Evidence from Controlled and Naturalistic Designs

**DOI:** 10.1177/08902070261449352

**Published:** 2026-05-22

**Authors:** Sophie C. Bauditz, Fabian Gander, Alexander Grob, Alex Christoph Traut, Maximiliane Uhlich, Ursula Hess, Matthias Ziegler

**Affiliations:** 1 Department of Psychology, 9373 Humboldt-Universität zu Berlin, Berlin, Germany; 2 Department of Psychology, 27209 University of Basel, Basel, Switzerland

**Keywords:** Big Five, ESM, person–situation relations, personality dynamics, situation perceptions

## Abstract

Personality is increasingly viewed as a dynamic system arising from the interaction between individuals and their situations. Yet, in naturalistic data, within-person links between personality states and situation perceptions often conflate two distinct mechanisms: situation contact—what people objectively encounter—and situation construal—how they interpret these encounters. To disentangle these components, we combined two longitudinal approaches: a standardized laboratory study (N = 189) in which situation contact was held largely constant, and a naturalistic experience-sampling study (N = 1,596) capturing the diversity of everyday contexts. Multilevel models revealed meaningful within-person covariation between Big Five states and situation perceptions in both designs, with effect sizes that were small to medium in magnitude and theoretically coherent across both settings. However, in the naturalistic study, person–situation associations varied systematically across situation cues—social partner, location, and activity—with some associations shifting in magnitude or reversing direction depending on the context. Together, these findings suggest that construal is a primary driver of person–situation associations, but that situation contact shapes which associations emerge, to what degree, and across which situation perception dimensions. Accounting for situation contact may therefore help to more accurately capture personality as a context-sensitive, dynamic system.

## Plain Language Summary

People act differently depending on the situation they are in, but it is unclear whether this is because of the kinds of situations they encounter or because of how they personally interpret those situations. To address this, we conducted two studies: a laboratory study (N = 189) in which participants experienced largely the same standardized situations, and a naturalistic study (N = 1,596) in which participants reported on their personality, their situation perceptions, and specific situation features (e.g., location, social partners, activity) multiple times throughout their daily lives. In both studies, how people perceived their situations was meaningfully linked to how they described their personality in that moment. However, in everyday life, these links changed depending on the specific situation people were in â€, for example, depending on whether they were alone or with friends. These findings suggest that understanding personality in everyday life requires considering not only how people interpret their situations but also what situations they actually encounter.

A foundational insight in personality psychology is that understanding behavior requires considering both the person and the situation (Hartshorne & May, 1928; Lewin, 1936; Mischel & Shoda, 1995). With the rise of experience-sampling methodology (ESM; Csikszentmihalyi & Larson, 1987) and advances in intensive longitudinal modeling (e.g., DSEM; Asparouhov et al., 2018) researchers can now study personality processes as they unfold in daily life. This work has shown that personality states—momentary expressions of traits—fluctuate meaningfully across situations (Fleeson, 2001; Fleeson & Jayawickreme, 2015, 2025) and are systematically linked to how individuals perceive those situations (e.g., Horstmann et al., 2021; Rauthmann et al., 2014, 2016; Sherman et al., 2015; Ziegler et al., 2019). However, a central limitation of these predominantly naturalistic studies is that assessments of situation perceptions conflate two conceptually distinct sources of variance: the kinds of situations individuals actually encounter (situation contact) and how they interpret those situations (situation construal; Rauthmann et al., 2015b). As a result, observed associations between situation perceptions and personality states may reflect differences in environmental exposure, differences in subjective interpretation, or both. For example, a positive link between momentary Neuroticism and perceived situational Negativity could arise because individuals encounter more stressful and negative environments—such as a conflict at work—or because they interpret neutral situations as more threatening. Separating these two sources is important for understanding the processes underlying person–situation dynamics, yet empirical work that directly addresses the role of contact and construal within a unified framework remains scarce (Abrahams et al., 2021; Morse et al., 2015; Rauthmann et al., 2015b). The present study addresses this gap using two complementary longitudinal designs. In a controlled laboratory study (Study 1) contact variability is constrained, allowing observed associations to primarily index construal. In a naturalistic ESM study (Study 2), contact variability is reintroduced. Beyond this, Study 2 examines whether features of situation contact—specifically, who participants are with, where they are, and what they are doing—moderate the associations between situation perceptions and personality states. Together, they provide evidence about the converging and contrasting roles of situation contact and construal in shaping person–situation dynamics.

## Personality: From Traits to States

Personality traits, defined “as characteristic, and automatic, patterns of thinking, feeling, and behaving that are consistent over time and across relevant situations” (Roberts & Yoon, 2022), are meant to describe how a person is *in general* across extended periods and diverse situations. While this trait-centric perspective has provided valuable insights, it seems not to fully encapsulate the complexity of people’s personalities (e.g., Baumert et al., 2017). As a result, researchers have increasingly focused on personality’s momentary manifestations, known as personality *states* (e.g., Fleeson, 2001; Fleeson & Jayawickreme, 2015). Personality states share the same affective, behavioral, and cognitive content as their corresponding traits but apply over shorter durations (Fleeson & Jayawickreme, 2015).

Empirical evidence highlights the within-person variability in Big Five personality states. For example, Fleeson (2001) demonstrated significant variability in the Big Five personality states within individuals, a finding corroborated by subsequent research (e.g., Horstmann et al., 2021; Sherman et al., 2015). For instance, intraclass correlations (ICCs) for Big Five personality states have been found to range from .18 to .33 (Horstmann et al., 2021), indicating that most of the variance in personality states lies within rather than between individuals. Interestingly, the extent of within-person variability differs across personality dimensions: among the Big Five, Extraversion exhibits the greatest moment-to-moment change, whereas Agreeableness is comparatively stable (e.g., Fleeson, 2001). Other personality constructs, such as narcissism (Bauditz et al., 2024), achievement motivation (Witte et al., 2024), and interests (Roemer et al., 2021), show comparatively less within-person variability, yet even these dimensions fluctuate meaningfully across situations. That personality states vary so substantially within individuals raises a natural question: what features of the situation drive these fluctuations?

## Situations: Concepts and Frameworks

Answering this question requires specifying what constitutes a situation—a challenge that has long occupied personality research. Numerous conceptualizations have been proposed (see Baumert et al., 2017, for a review). In a widely cited integrative effort, Baumert et al. (2017) define a situation as “a set of circumstances outside the person consisting of objectively quantifiable properties (often including other people) that may be perceived and interpreted by a person” (p. 528).

To establish a standardized, consistent terminology, Rauthmann, Sherman, and Funder (2015) distinguish three levels of situational information: cues, characteristics, and classes. Cues are the objective, measurable features of a situation—the location (e.g., a conference room), the social partner (e.g., colleagues), or the ongoing activity (e.g., preparing a presentation). They capture the structural composition of a situation. However, it has been proposed that the psychological impact of a situation likely depends less on these objective features per se than on how individuals perceive and interpret them (e.g., Furr & Funder, 2018; Rauthmann, Sherman, & Funder, 2015). For instance, the same conference-room context may feel socially evaluative and stressful to one person but energizing and opportunity-rich to another. These subjective appraisals are termed situation characteristics (or perceptions) and capture the psychologically salient meanings that people attach to situation cues (Rauthmann, Sherman, & Funder, 2015). Finally, classes denote broader categories that group situations by shared features, defined either by objective criteria (e.g., professional vs. affiliative contexts) or psychological similarity (e.g., stressful vs. energizing situations).

Although several proposals exist, we are not aware of any broadly accepted general taxonomy of situation cues and classes that has been established to date. In contrast, multiple taxonomies have been proposed to capture situation perceptions, such as the CAPTION (Parrigon et al., 2017), the DIAMONDS (Rauthmann et al., 2014), Situational Affordances for Adaptive Problems (SAAP: Brown et al., 2015), or the Situation Five (Ziegler et al., 2019). A comparative analysis of these taxonomies found that they largely overlap in content, converging on what has been termed the “replicable six” (Rauthmann & Sherman, 2018a), though a fully validated integrative taxonomy has not yet been empirically established. Most previous research has employed the DIAMONDS framework (Rauthmann et al., 2014), which assesses situation perceptions along eight key dimensions: Duty, Intellect, Adversity, Mating, pOsitivity, Negativity, Deception, and Sociality. The current research also employs the DIAMONDS to operationalize situation perceptions.

## Person–Situation Relations: Theory and Empirical Evidence

Understanding behavior has long been a central aim of personality research. Early work recognized that behavior is jointly shaped by the person and the situation (Hartshorne & May, 1928; Lewin, 1936), but it was Mischel’s (1968) critique of trait predictive validity that sparked the person–situation debate over the relative importance of stable dispositions versus contextual factors. This debate, in turn, catalyzed the development of social-cognitive personality theories (Mischel, 1973) that place situational processes at the center of personality functioning. Among the most influential frameworks are the Cognitive-Affective Processing System (CAPS; Mischel & Shoda, 1995), which conceptualizes personality as a network of cognitive-affective units that translate situational inputs into behavioral outputs, and the Whole Trait Theory (WTT; Fleeson & Jayawickreme, 2015, 2025), which proposes that traits reflect density distributions of momentary states, emphasizing the variability and situational sensitivity of personality expression. Across these frameworks, a shared conclusion emerges: to understand how personality unfolds in everyday life, it is essential to consider the situations individuals encounter and how they respond to them.

In recent years, empirical work has documented person–situation relations with increasing precision. Using standardized situation perception taxonomies such as the DIAMONDS, research has established robust associations between situation perceptions and personality states—for instance, perceiving an encounter as highly social is associated with higher state Extraversion (e.g., Horstmann et al., 2021; Parrigon et al., 2017; Rauthmann et al., 2014; Sherman et al., 2015; Ziegler et al., 2019). However, these associations are often broad and diffuse: in naturalistic data, most situation perception dimensions tend to correlate with most personality states. On the person side, this breadth may arise because individuals systematically differ in how perceptions and states are linked—for instance, accounting for momentary affect attenuates or eliminates several perception–state associations (Horstmann et al., 2021; see also Rauthmann, 2016). By the same logic, on the situation side, broad associations may reflect aggregation across heterogeneous situation cues: the same level of perceived Sociality, for example, can arise from very different constellations of activities, locations, or interaction partners, each carrying distinct affordances for personality expression (Rauthmann et al., 2014). Thus, disentangling situation contact from situation construal is a central methodological challenge for advancing person–situation research. Without separating the situations individuals encounter from how they subjectively interpret them, observed perception–state associations remain difficult to interpret.

## Situation Contact and Situation Construal

The distinction between contact and construal has a long theoretical history. Murray (1938) differentiated alpha-press—the objective properties of a situation—from beta-press—the situation as perceived by the individual (see also Rauthmann, 2021; Rauthmann et al., 2015b). In naturalistic designs, this distinction becomes empirically consequential because both sources of variance operate simultaneously when assessing situation perceptions. Consider two commuters stuck in the same traffic jam: one feels frustrated and trapped, perceiving the situation as stressful; the other uses the time to listen to music and feels calm. Despite identical contact, their expressions of personality states differ because they construe the situation differently. But naturalistic designs introduce an additional complication: not only do individuals differ in how they interpret situations, but they also differ in which situations they encounter in the first place. Some individuals may frequently find themselves in stressful environments, whereas others rarely do. When these two sources—differential exposure and differential interpretation—are not separated, observed associations between situation perceptions and personality states may reflect contact, construal, or both. This confound limits our ability to determine whether momentary variation in personality states is primarily driven by external situational features or by internal interpretive processes—a question central to understanding the mechanisms underlying person–situation dynamics.

### Naturalistic Studies

Several studies have attempted to empirically separate contact and construal within naturalistic designs. Rauthmann et al. (2015b) asked participants to describe their daily situations using W-questions (e.g., “Where were you?”; “What was happening?”) alongside their own DIAMONDS ratings (in situ). Independent ex situ raters later evaluated these W-question descriptions on the DIAMONDS dimensions, providing an external interpretation of the same situations. These ex situ ratings served as proxies for situation contact, whereas the discrepancy between in situ and ex situ ratings was interpreted as reflecting situation construal. Hong et al. (2020) implemented a similar multi-rater design. Abrahams et al. (2021) went a step further in a multi-informant experience-sampling study in which co-present observers (juxta situm)—who actually shared the situational context—provided DIAMONDS ratings alongside the focal participants. Because observer ratings are not filtered through the focal participant’s selective reporting, this approach more directly operationalized situation contact.

Across these studies, findings converge on the relative importance of construal. Rauthmann et al., 2015b documented associations between Big Five traits and DIAMONDS dimensions that were small to moderate in magnitude—for example, Neuroticism was negatively associated with Positivity, with this link driven primarily by construal rather than contact. Hong et al. (2020) extended these findings by showing that the effects of personality pathology on situation perception were predominantly construal-driven, with construal effects larger than corresponding contact effects. Abrahams et al. (2021) similarly found that in situ perceptions were more strongly predictive of personality states than juxta situm perceptions, highlighting construal as a central mechanism for personality expression.

Despite these advances, important limitations remain. When ex situ judgments are derived from participant-generated open descriptions, the input already reflects what participants deemed salient or worth reporting, thereby reintroducing construal into what is treated as the objective baseline. Even co-present observers bring their own subjective lens—unless ratings are based on expert judges or sufficiently large rater pools, they remain susceptible to rater-specific construal. Moreover, all three designs operationalized both contact and construal through perception ratings (e.g., DIAMONDS), differing only in the rater’s perspective. This leaves the contact side of the equation captured only indirectly—through consensus or external perception ratings—rather than through structured assessment of situation features themselves (e.g., location, activity, or social partners). In naturalistic designs, self-reported situation cues offer one step toward a more direct operationalization of contact, though they too remain subject to reporting biases.

### Laboratory Studies

Laboratory designs offer a complementary strategy by directly constraining contact: when all participants are exposed to the same standardized conditions, variance in situation ratings can be attributed primarily to individual differences in construal. Serfass and Sherman (2013) took this approach by presenting participants with identical ambiguous images from the Thematic Apperception Test (TAT) and asking them to rate each image’s psychological features. Another example comes from Morse et al. (2015), who had participants rate their perceptions (Situational Q-sort) of three standardized in-lab interactions with previously unacquainted others. Because all participants experienced a largely similar interactional paradigm, variance in situation ratings could again be attributed primarily to construal.

Both studies found meaningful links between personality and construal. Serfass and Sherman (2013) observed substantial within-situation agreement across raters alongside consistent individual differences in construal that were meaningfully linked to Big Five traits. Neuroticism was positively associated with perceptions of threat and interpersonal criticism and negatively associated with more positive situational features, whereas Openness predicted ratings of situational complexity and intellectual content. Morse et al. (2015) similarly found that Neuroticism was negatively associated with the positivity of situation construal, whereas Extraversion was positively linked to construal positivity and to downstream social outcomes. Notably, situation construal did not fully mediate the personality–outcome associations, suggesting that although construal plays a meaningful role, additional pathways also contribute to how personality traits translate into social behavior.

Despite these contributions, limitations remain. Serfass and Sherman (2013) relied on ecologically limited image-based stimuli, and their focus on traits rather than states leaves open how momentary personality expressions interact with differences in construal. Morse et al. (2015) similarly measured personality at the trait level, leaving the within-person dynamics of construal and personality states unexamined in a controlled setting.

## The Current Studies

**Figure 1.**
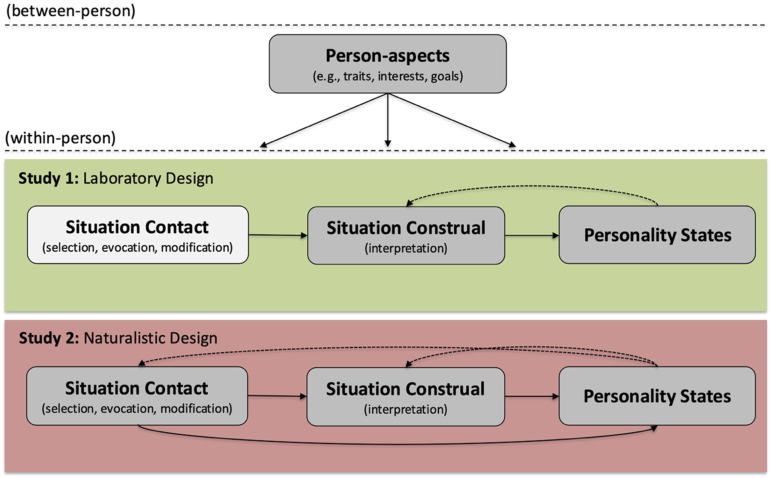
Conceptual model linking situation contact, situation construal, and personality states. *Note.* Between-person aspects (e.g., traits, interests, goals) influence momentary person–situation relations. At the within-person level, situation contact (exposure to situation cues) and situation construal (interpretation of situational meaning) jointly shape momentary personality state expression. Solid arrows represent situational effects; dashed arrows indicate feedback and reciprocal processes between situations and states. In Study 1, situation contact is standardized, whereas it is reintroduced in Study 2

The present research examines the within-person relations between Big Five personality states and situation perceptions across two complementary longitudinal designs: a controlled laboratory study and a naturalistic experience-sampling study. Both studies address the same core question, but through different approaches to situational control (Figure 1):

**RQ1:** How do Big Five personality states and situation perceptions co-occur within persons?

In the laboratory study, participants repeatedly interacted in standardized online group situations designed to hold situation contact largely constant. By minimizing opportunities for situation navigation mechanisms (e.g., situation selection, evocation, or modification; Buss, 1987; Rauthmann et al., 2015b; Rauthmann & Sherman, 2016)—the design ensured that participants were exposed to comparable situational inputs. To limit variability due to interactional dynamics, research assistants followed a standardized session protocol, and the procedure was regularly interrupted by short questionnaire assessments (approx. every 15 minutes), which limited the degree to which sessions could diverge from the standardized format over time. Within this controlled yet dynamic context, differences in participants’ situation ratings should predominantly reflect differences in construal rather than differences in contact. Beyond examining RQ1 across all sessions, we also tested whether co-occurrence patterns differed between two experimentally varied discussion conditions (affiliative vs. controversial).

The naturalistic study captured participants’ everyday experiences, during which both construal and contact varied freely. In addition to DIAMONDS and Big Five state ratings, participants reported three situation contact features at each assessment: (1) social partner (e.g., alone, with friends, with a romantic partner), (2) location (e.g., at home, in nature, in public spaces), and (3) activity (e.g., working, relaxing, socializing). Unlike the rater-comparison approaches used in prior naturalistic work, these cue variables operationalize contact through structured self-reports of situation features rather than through external perception ratings. Although still based on self-report, they provide a more neutral, less construal-laden account of the situations participants encountered, enabling systematic tests of whether person–situation associations vary as a function of situational context. This leads to the second research question:

**RQ2:** Are the within-person associations between Big Five states and situation perceptions, identified in RQ1, moderated by the situation variables reported in the naturalistic design?

Together, the two studies form a complementary approach: the laboratory study constrains contact variability, allowing observed associations to reflect construal primarily; the naturalistic study reintroduces contact variability and directly tests whether contact features modulate person–situation links. This approach does not fully disentangle construal and contact in a strict experimental sense, but it provides converging evidence on the roles each source plays in shaping person–situation dynamics.

## Study 1

Study 1 leverages a standardized laboratory paradigm to probe within-person associations between Big Five personality states and situation perceptions, holding situation contact largely constant. Across six weekly online group interactions, participants first engage in affiliative discussions, then in more contentious ones. This sequence mirrors a naturalistic feature of social exchange—conversations typically begin with more superficial, rapport-building topics before moving toward more serious or controversial ones (e.g., political debates). This structured setup minimizes variance in contact attributable to navigation mechanisms such as *situation selection*, *evocation*, or *modification*; Rauthmann et al., 2015b; Rauthmann & Sherman, 2016). Under these constraints, any systematic covariation between states and perceptions should primarily reflect situation construal. Based on prior work using the DIAMONDS taxonomy, we derived directional hypotheses for the expected co-occurrence patterns, as summarized in Table 1.^1^

**Table 1.** Hypotheses for Research Question 1: Relations between the Big Five states and DIAMONDS

States	D	I	A	M	O	N	De	S
Extraversion	-	-	-	+	+	-	-	+
Conscientiousness	+	+	-	-	+	-	-	​
Agreeableness	-	-	-	+	+	-	-	​
Neuroticism	​	+	+	+	-	+	+	​
Openness	+	+	-	+	+	-	-	+

*Note.* The hypotheses build on the findings by Horstmann et al. (2021). The correlations refer to the overall Level 1 co-occurrence between the Big Five dimensions and DIAMONDS, as assessed in the multilevel models. Notably, associations involving the Mating dimension are expected to be more prominent in the ESM data, as the experimental data included only same-sex groups. D = Duty; I = Intellect; A = Adversity; M = Mating; O = pOsitivity; N = Negativity; De = Deception; S = Sociality. + = positive association expected; - = negative association expected; empty cells show that no relation was expected.

## Methods

### Procedures

**Figure 2.**
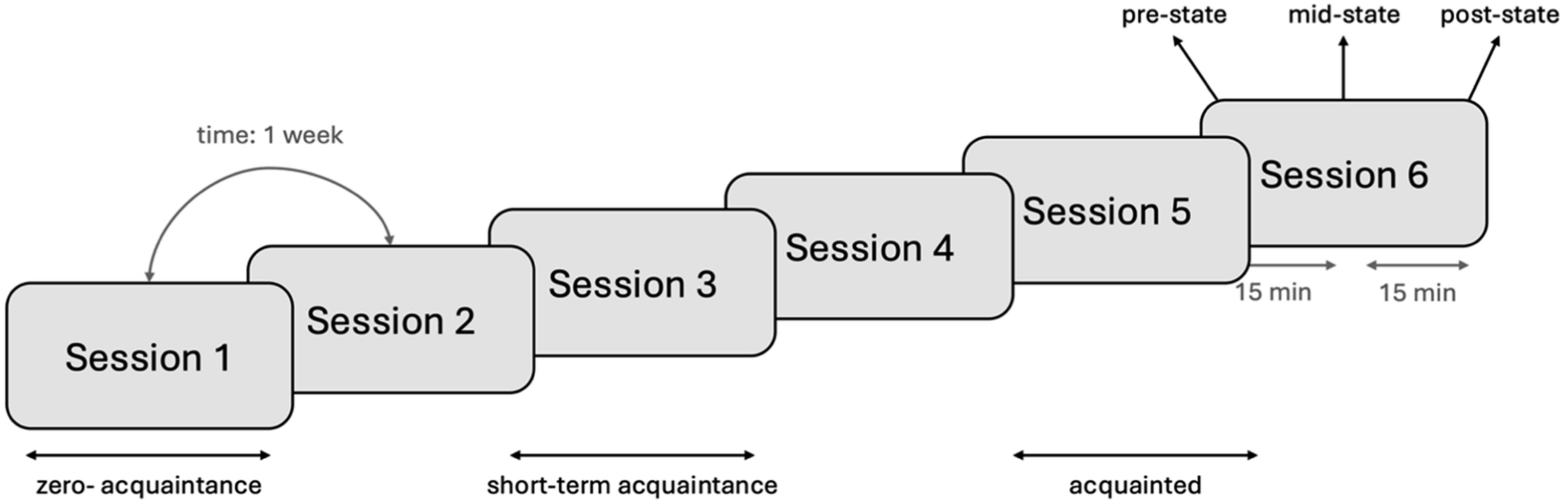
Experimental setup of Study 1. *Note.* Each group discussion session included two fifteen-minute segments, with the entire session, including state assessments, lasting approximately 1 hour

Before the laboratory onset, participants completed an extensive test battery, including trait assessments of the Big Five. Participants were then randomly assigned to groups based on availability, ensuring that group members were not previously acquainted. Over the 6 weeks, participants met weekly in groups of three to four (see Figure 2). Sessions were conducted via Zoom and facilitated by a trained research assistant to ensure adherence to the discussion protocol. The sessions were split into two conditions, which were designed to vary the interpersonal and evaluative demands of the situation while keeping the social format constant: affiliative sessions promoted positive social exchange (e.g., introductions, discussion of shared interests), whereas controversial sessions introduced morally complex dilemmas intended to heighten interpersonal tension and evaluative threat.

A manipulation check, published in Bauditz et al. (2025), confirmed significant changes across the study conditions. Specifically, Positivity decreased from *M* = 3.91 (*SD* = 1.28) in the first three meetings to *M* = 3.48 (*SD* = 1.34), *t* = 8.29, *p* < .001, *d* = 0.61, and Adversity increased from *M* = 1.25 (*SD* = 0.66) to *M* = 1.46 (*SD* = 0.91), *t* = −5.74, *p* < .001, *d* = −0.42. Mating and Sociality both declined significantly across sessions (*t* = 3.92, *p* < .001, *d* = 0.29; *t* = 3.85, *p* < .001, *d* = 0.28, respectively), whereas Duty, Intellect, Negativity, and Deception showed no significant differences after alpha correction for multiple testing.

Each meeting lasted approximately 1 hour and followed pre-defined discussion prompts. Before each session (pre-assessment), participants completed questionnaires assessing their momentary personality states and perceptions of the situations they had just experienced. After approximately 15 minutes of group discussion (mid-assessment), the interaction was paused, and participants again reported their perceptions of the situation. At the end of each group session (post-assessment), both personality states and situation perceptions were assessed once more.

### Participants

Participants were recruited via online platforms (including university department websites and a psychology mailing list) and via flyers distributed on campus. Eligible participants were 18–35 years old, currently enrolled at a German university, and proficient in German; prior acquaintance among group members was not permitted. Participants received €100 for completing the full study (seven one-hour laboratory sessions plus approximately 2.5 hours of online questionnaires, totaling 9.5 hours), with pro-rata compensation for those who withdrew early. A total of 385 participants provided trait data. State information was collected from the 220 participants included in the group experiment. As pre-registered, we removed data from participants who (i) had more than 25% missing responses, (ii) exhibited no variance across all surveys at any measurement time point, or (iii) attended fewer than four group sessions. We also excluded individual reports that contained more than 20% missing values. The final sample for the laboratory design included 186 participants (72.58% female, 26.34% male, and 1.1% identifying as other; mean age *M* = 24.40 years; *SD* = 3.83). These participants contributed to 3,094 assessment occasions (*M*_*assessment occasions*_ = 16.63, *SD*_*assessment occasions*_ = 1.85).

#### Measures

##### Big Five Traits

We assessed personality traits with the 60-item German version of the Big Five Inventory (BFI-2; Danner et al., 2019; Soto & John, 2017). Participants rated their agreement on a six-point scale, with endpoint labels ranging from “disagree strongly” to “agree strongly.” The internal consistencies of the different domain scale scores were estimated to range between ω = .82 for Agreeableness and ω = .88 for Neuroticism, Extraversion, and Conscientiousness.

##### Big Five States

We assessed personality states, with the Personality State Scale (Ringwald et al., 2022), a 10-item daily Big Five questionnaire with two items per domain. Participants were presented with a rating scale that had one adjective at each endpoint. They were asked to rate the extent to which the term described themselves at this moment on a seven-point rating scale. For example, “*hardworking* describes me extremely well” (1), “both describe me equally well” (4), “*lazy* describes me extremely well” (7). The estimated Level 1 internal consistency for the Personality State Scale in the laboratory data was between ω_weighted_ = .41 for Agreeableness and ω_weighted_ = .71 for Extraversion.

##### Situation Perceptions

To assess situation perception, the Situation Eight DIAMONDS ultra-short measure in its original German version (Rauthmann & Sherman, 2018b) was used. The DIAMONDS assess Duty (e.g., “work needs to be done”), Intellect (e.g., “deep thinking is required”), Adversity (e.g., “someone is being threatened”), Mating (e.g., “potential romantic partners can be attracted”), pOsitivity (e.g., “the situation is positive, playful”), Negativity (e.g., “the situation could be tainted by negative feelings”), Deception (e.g., “someone can be deceived”), and Sociality (e.g., “meaningful social interaction is possible or required”). Situation perceptions were rated on a six-point rating scale, with the endpoint designations “not at all” (1) and “totally” (6).

#### Analytical Strategy

Statistical analyses were conducted in R (v.4.3.3; R Core Team, 2022) with the packages *tidyverse* (v.2.0.0; Wickham et al., 2019), *lme4* (v.1.1.35.3; Bates et al., 2014), *lavaan* (v.0.6.16; Rosseel, 2012), and *bootnet* (v.1.5.1; Epskamp et al., 2018). Due to the nested structure of the data, we applied multilevel models (MLM; Hox, 2010; Nezlek, 2008) to estimate the relation between situation perceptions and the Big Five personality states (dependent variables). Before fitting the multilevel models, the Level 1 predictors (situation perceptions) were person-mean-centered, so that the regression coefficients reflect within-person effects. Person means of situation perceptions and personality traits were included as grand-mean-centered Level 2 predictors (Enders & Tofighi, 2007). To fit the MLMs, we adhered to a step-by-step procedure. Initially, unconditional random-intercept models (Model I: Null Model) were estimated separately for each Big Five state and for each DIAMONDS situation perception. These models included only a random intercept for individuals (Level 2) and were used to estimate intraclass correlations (ICCs) for both constructs independently. In all subsequent models, personality states served as the sole dependent variables. First, for each personality dimension separately, we entered the corresponding personality trait scores at Level 2 to control for the Big Five traits on Big Five states (Model II: Big Five Trait). Next, we included situation perceptions as a Level 1 and Level 2 predictor (Model III: Situation Perceptions Fixed). Lastly, random effects for the Level 1 effect of situation perceptions were added to allow for person-specific deviations (Barr et al., 2013; Model IV: Situation Perceptions Random). The group was included as an additional Level 3 nesting variable in all models.

Following our pre-registered analysis plan, we first tested the pre-specified models. This approach resulted in 160 distinct models. To select the best-fitting model, we used information criteria. As suggested by Vallejo et al. (2014), we estimated the Akaike’s Information Criterion (AIC) values to compare the models. For the pre-registered hypothesized associations, we applied tests with an alpha level of .05. For the small number of associations for which no directional hypothesis was pre-registered, we applied Bonferroni-Holm correction to control the family-wise error rate. All inferential tests reported in the manuscript are two-tailed.

Two exploratory analyses were conducted. First, we extended Model IV to include all eight situation perceptions as predictors of each Big Five dimension, examining the unique contribution of each perception while controlling for the others, thereby providing a more stringent test of their relative importance. Second, we tested all person–situation relations separately for the two experimental manipulation conditions (affiliative vs. controversial). As these condition-specific analyses were not part of the pre-registered hypotheses, Bonferroni-Holm correction was applied to control for the increased number of tests.

#### Deviations From Pre-Registration

The pre-registration included an additional focus on person–situation transactions as a second main research question; however, as the project evolved, it became clear that addressing both aspects within a single paper would compromise conceptual clarity and focus. Moreover, the pre-registration specified three assessment points for all measures. However, the pre-registration was written before the data were inspected, and inaccurately described the assessment schedule. In practice, the study protocol collected Big Five state assessments at two time points (pre- and post-session), whereas situation perceptions were assessed at all three time points (pre-, mid-, and post-session). This discrepancy reflects a documentation error in the pre-registration, not a post-hoc change to the study protocol.

## Results

### Descriptives

Table 2 presents descriptive statistics for the trait measures.

**Table 2.** Descriptive Statistics for Trait Measures in the Laboratory Design

Construct	*M*	*SD*	1	2	3	4	Correlation trait-aggregated state
1. Extraversion	4.06	0.77	-	​	​	​	.35***
2. Conscientiousness	4.20	0.78	.25***	-	​	​	.48***
3. Agreeableness	4.51	0.61	.23***	.22***	-	​	.46***
4. Neuroticism	3.26	0.78	−.34***	−.26***	−.22***	-	.52***
5. Openness	4.46	0.70	.35***	.08***	.27***	−.21***	.34***

*Note. r* trait-state_agg_ indicates the correlation between the trait and the corresponding aggregated state across all measurement occasions.

*p < .05. **p < .01. ***p < .001.

Table 3 presents the aggregated state correlations and the intraclass correlation coefficients (ICCs) for the Big Five states and DIAMONDS dimensions. ICCs for the Big Five state variables range from .29 (Extraversion) to .56 (Agreeableness), indicating substantial within-person variance. Regarding the situation perception dimensions, Adversity and Deception were the lowest rated. Mating perception was also notably lower. The within-person variance in Duty was particularly high. The within-person variability, as indicated by the ICCs, ranged from .23 (Adversity) to .53 (Mating). This is a similar range to the Big Five states. Taken together, the findings indicate substantial within-person variation that warrants closer examination of how personality states and situation perceptions co-occur.

**Table 3.** Descriptive Statistics for Aggregated and Non-Aggregated State Measures in Laboratory Study

	*M*	*SD*	1	2	3	4	5	6	7	8	9	10	11	12	Non-aggregated states ICC [95% CI]
Big Five states															
1. Extraversion	4.37	0.72													.29 [.24; .34]
2. Conscientiousness	4.49	0.76	.60***												.42 [.36; .48]
3. Agreeableness	4.03	0.82	.53***	.39***											.56 [.50; .61]
4. Neuroticism	3.64	0.8	−.63***	−.38***	−.53***										.38 [.32; .43]
5. Openness	4.14	0.79	.66***	.48***	.54***	−.44***									.44 [.37; .49]
Situation perception															
6. Duty	3.58	1.12	−.04	.08	−.15*	.21**	.09								.44 [.38; .50]
7. Intellect	3.72	0.99	.07	.11	.02	.03	.26***	.61***							.38 [.32; .43]
8. Adversity	1.34	0.42	−.18*	−.12	−.36***	.23**	−.17*	.22**	.21**						.23 [.19; .28]
9. Mating	1.53	0.94	−.01	.06	.02	.01	.13	.24***	.18*	.16*					.53 [.47; .58]
10. Positivity	3.69	0.8	.60***	.37***	.47***	−.48***	.46***	<.01	.20**	−.22**	.04				.32 [.27; .37]
11. Negativity	2.16	0.72	−.37***	−.18*	−.36***	.61***	−.22**	.30***	.24**	.51***	.22**	−.33***			.27 [.22; .31]
12. Deception	1.25	0.42	−.31***	−.22**	−.38***	.35***	−.24**	.21**	.11	.70***	.13	−.36***	.47***		.38 [.32; .43]
13. Sociality	4.43	0.88	.16*	.14	.21**	−.03	.23**	.20**	.41***	−.11	.16*	.43***	.03	−.20**	.26 [.21; .31]

*Note.* *p < .05, **p < .01, ***p < .001.

### Co-Occurrence of Big Five States and Situation Perceptions

To address Research Question 1, we conducted a series of multilevel linear models (Models I–IV). For the majority of Big Five state–situation perception pairs (28 of 40), the model including random slopes provided the best fit, indicating meaningful individual differences in person–situation contingencies even under controlled conditions. For the remaining 12 pairs—concentrated on Adversity, Mating, and Deception—the simpler fixed-effects model was preferable. This pattern likely reflects the low mean levels of perceived Adversity, Mating, and Deception in our controlled lab situations (see Table 3), which restricted between-person variability and thus provided little information for estimating random slopes. Table 4 presents the results based on the best-fitting models. Analyses of the relationships between Big Five personality states and situation perceptions provided mixed support for our hypotheses (20 out of 36). To complement these single-predictor models, we also estimated models in which all situation perceptions were entered simultaneously as predictors of each Big Five state, thereby controlling for shared variance among the DIAMONDS dimensions (see Table S4). Results were largely consistent, suggesting that the observed associations are not solely driven by overlap between situation perception dimensions.

**Table 4.** Unstandardized Fixed L1 Effects of Within-Person Centered Situation Perceptions on Big Five States in Laboratory Data

​	Extraversion			Conscientiousness			Agreeableness			Neuroticism			Openness		
	*b*	[CI]	*t*	*b*	[CI]	*t*	*b*	[CI]	*t*	*b*	[CI]	*t*	*b*	[CI]	*t*
D	−0.12***	[-0.18; −0.07]	−4.7	−0.01	[-0.06; 0.03]	−0.67	-0.10***	[-0.13; −0.07]	−6.1	0.19^†††^	[0.16; 0.23]	8.42	−0.11***	[-0.15; −0.07]	−5.3
I	0.06*	[0.02; 0.11]	2.57	0.07***	[0.03; 0.11]	3.83	-0.01	[-0.04; 0.02]	−0.61	0.04	[-0.01; 0.08]	1.76	0.04*	[0.00; 0.08]	2.14
A	−0.02^a^	[-0.08; 0.05]	−0.45	<.001^a^	[-0.05; 0.07]	0.01	-0.13***	[-0.19; −0.07]	−4.53	0.14^a^ ***	[0.08; 0.20]	4.43	−0.01^a^	[-0.07; 0.04]	−0.36
M	−0.05^a^	[-0.10; 0.01]	−1.93	−0.03^a^	[-0.05; 0.05]	−1.33	-0.02^a^	[-0.06; 0.01]	−1.25	0.08*	[0.02; 0.14]	2.5	0.03^a^	[-0.01; 0.07]	1.34
O	0.32***	[0.26; 0.37]	11.81	0.15***	[0.11; 0.20]	6.46	0.16***	[0.12; 0.20]	8.34	−0.32***	[-0.36; −0.27]	−12.67	0.25***	[0.21; 0.29]	12.73
N	−0.24***	[-0.28; −0.19]	−10.24	−0.05*	[-0.09; −0.01]	−2.22	-0.14***	[-0.18; −0.11]	−7.66	0.36***	[0.32; 0.40]	16.35	−0.14***	[-0.18; −0.10]	−6.56
De	−0.01^a^	[-0.10; 0.08]	−0.26	<.001^a^	[-0.07; 0.08]	0.05	-0.15^a^***	[-0.21; −0.09]	−4.78	0.15*	[0.04; 0.26]	2.57	0.01^a^	[-0.06; 0.09]	0.31
S	0.16***	[0.12; 0.20]	7.54	0.07^†††^	[0.04; 0.10]	3.95	0.06^†††^	[0.04; 0.09]	4.49	−0.11^†††^	[-0.15; −0.07]	−4.59	0.13***	[0.09; 0.16]	6.97

*Note.* Model selection was based on the lowest AIC value. Coefficients refer to Model IV, except for models with a subscript ^a^, for which Model III is reported, as the AIC indicated a better fit. Confidence intervals are two-tailed 95% CIs. *b* = estimate (unstandardized) effect; *t* = t-value. D = Duty, I = Intellect, A = Adversity, M = Mating, O = Positivity, N = Negativity, De = Deception, S = Sociality. *p < .05, **p < .01, ***p < .001. For the few associations without hypotheses, we used Bonferroni-Holm adjustments to the *p*-values. ^†††^*p* Holm-adj. < .001, for the associations with no hypotheses.

### Affiliative vs. Controversial Sessions

Analyses of within-person associations between situation perceptions and Big Five states across the two manipulation conditions (see Tables S1 and S2 for affiliative and controversial sessions) revealed a largely consistent pattern of effects. To quantify this consistency, we compared the standardized within-person regression coefficients obtained separately for each condition (Table S3). The Pearson correlation between the two 40-element effect-size profiles was *r* = .97 (Figure S1), confirming that the overall architecture of person–situation associations was highly rank-consistent across conditions. The largest descriptive shifts between conditions were observed for Extraversion–Negativity (β = −.25 in the affiliative vs. −.15 in the controversial condition) and Extraversion–Adversity (β = −.04 vs. .03). Though the Extraversion–Adversity association changed sign across conditions, both coefficients were non-significant and their 95% confidence intervals overlapped substantially.

## Discussion

The laboratory results allow a construal-focused interpretation. When situation contact was largely held constant, within-person associations between DIAMONDS perceptions and Big Five states were small to medium in magnitude (*b* = 0.01–0.36 on a 1–6 scale), consistent with the interpretation that construal processes alone produce modest but theoretically coherent perception–state links. The pattern of associations aligned with what would be expected given the objective features of the laboratory setting (i.e., online same-sex group discussions): Positivity covaried positively with Extraversion, Conscientiousness, Agreeableness, and Openness, whereas Negativity and Adversity covaried positively with Neuroticism and negatively with communal or agentic states. Sociality showed positive associations with Extraversion; Intellect was modestly associated with Extraversion, Conscientiousness, and Openness. Mating showed weak or inconsistent associations, as expected in a non-romantic, task-oriented context.

To further examine the role of situation contact within this constrained framework, we split the analyses by manipulation condition (affiliative vs. controversial). This step tested whether subtle variations in situation contact—introduced by differing discussion topics—would moderate person–situation associations even while the overall situational frame remained constant. The overall architecture of person–situation associations was highly consistent across conditions, suggesting that construal processes operate largely independently of the specific contact manipulation. The largest descriptive shift was found in the Extraversion–Adversity link. Together, the theoretically interpretable associations under controlled contact (e.g., weak Mating associations in same-sex groups) and the slight condition-specific shifts suggest that situation contact plays a role in shaping person–situation relations—even when its variability is minimal. These findings motivate the complementary analyses in Study 2, which reintroduce contact variability in a naturalistic setting to examine its contribution more directly.

## Study 2

Study 2 examined person–situation associations in a naturalistic ESM design, in which both situation contact and situation construal were allowed to vary. In addition to the Big Five states and situation perceptions, participants reported structured situation cues indicating who was present, where participants were, and what they were doing. These cue reports provide an approximation of situation contact, as they capture features of the situation participants encountered rather than their psychological interpretation of those situations. At the same time, because these cues were self-reported they should not be treated as fully objective indicators of contact. Study 2 therefore complements Study 1 in two ways. First, it tests whether the within-person associations between Big Five states and situation perceptions observed under relatively standardized contact conditions also emerge in everyday life, where situation contact varies freely. Second, it examined whether these associations are moderated by contact-relevant situation cues. Evidence for such moderations would indicate that person-situation associations are not solely a function of construal, but also depend on the broader situational context in which perceptions arise. Importantly, Study 2 does not disentangle contact and construal in a strict sense: naturalistic situation ratings necessarily reflect both the situations individuals encounter and how they interpret them. Rather, by examing whether perception-state associations vary across social partners, locations, and activities, Study 2 provides indirect evidence for the role of situation contact in shaping person-situation dynamics. 

## Methods

### Procedures

Study 2 was part of a broader longitudinal project investigating long-term personality development and relationship dynamics; the multi-wave design (five waves separated by approximately 5 months) and the target age range (18–40 years) were chosen to capture a developmentally active period for both personality and relationships. Each wave began with more extensive surveys assessing personality, including the Big Five traits. During every ESM phase, participants received text messages at five randomly distributed time points throughout the day: 8:05–9:50 AM, 11:05 AM–12:50 PM, 2:05–3:50 PM, 5:05–6:50 PM, and 8:05–9:50 PM. In these brief surveys, participants reported their current personality states and situation perceptions or reflected on their states and situations just before completing the survey. Each survey remained open for 1 hour to allow flexibility in response time.

### Participants

The sample comprised German-speaking residents from Germany, Austria, and Switzerland. Participants were recruited via online advertising on social networks (Facebook, Instagram, TikTok, Reddit) and mailing lists, as well as through flyers and posters. Compensation was a shopping voucher (€/CHF 10) per completed wave or lottery entries for prizes up to €/CHF 2,000; students could additionally opt for partial course credit. The recruited sample sizes for waves one to five were 1,731, 1,352, 1,229, 1,148, and 1,112 participants, respectively. After applying the same exclusion criteria to the data, as has been done for the laboratory study—except for point (iii) where we only included participants in a wave that had at least six assessment points in this wave—the final sample sizes across the five waves were 1,563, 1,251, 1,168, 1,071, and 1,045. The total sample size—number of individuals in the data set—was 1,596 (70.2% female, 27.3% male, 1.94% identifying as other, and 0.5% not providing any information; mean age, *M* = 28.67 years; *SD* = 5.73). These participants contributed to 72,211 assessment occasions (*M*_*assessment occasions*_ = 45.2 per person, *SD*_*assessment occasions*_ = 20.3). The average assessment points per wave were *M*_*assessment occasions/wave*_ = 11.8 (*SD*_*assessment occasions/wave*_ = 1.93).

#### Measures

##### Big Five Traits

To assess personality traits, we used the shorter 30-item German version (Rammstedt et al., 2020) of the Big Five Inventory (BFI-2; Soto & John, 2017). Study 2 used a five-point scale with endpoint labels ranging from “disagree strongly” to “agree strongly.” The internal consistencies of the different domain scale scores were estimated to range between ω = .69 for Agreeableness and ω = .82 for Neuroticism.

##### Big Five States

To assess personality states, we used the Five-Factor Model Personality States Inventory (Gander et al., 2025). It includes three items per Big Five domain, thus a total of 15 items per assessment. The items are rated on a 7-point bipolar scale, such as *shy* describes me extremely well (1), both describe me equally well (4), *sociable* describes me extremely well (7). The estimated Level 1 internal consistency for the Personality State Scale in the naturalistic data ranged from ω_weighted_ = .66 for Extraversion to ω_weighted_ = .83 for Neuroticism.

##### Situation Perceptions

To assess situation perceptions, the Situation Eight DIAMONDS ultra-short measure in its original German version (Rauthmann & Sherman, 2018b) was used. In addition to the eight dimensions as described in Study 1, the study also included an item to assess Typicality (“The situation is ordinary (typical for my everyday life)”) as the ninth situation perception variable (Parrigon et al., 2017; Roemer et al., 2021). Situation perceptions were rated on a seven-point rating scale, with the same endpoint designations “not at all” (1) and “totally” (7).

##### Situation Variables

To approximate the situational context participants find themselves in during the naturalistic design, three items were included to assess situation cues. These items focus on social partners (i.e., *alone*, *close relationships*, *work relationships*, and *other people*), the location (*private spaces*, *public*/*social spaces*, *outdoor spaces* (*nature*), and *other*), and the activity participants are engaged in (i.e., *relaxation*/*leisure*, *travel*, *responsibilities*/*work*, *social*/*interaction*, and *other*). The response options for these items vary in number: seven for social partner, ten for the location, and twelve for the activity. To simplify the analysis, these responses were clustered into three categories—social partner, location, and activity—for each variable, as pre-registered.

#### Analytical Strategy

The statistical approach for the naturalistic data followed the same multilevel modeling framework as described in Study 1. Due to the additional “Typicality” situation perception*,* this yielded a total of 180 models. To address research question two, we pre-registered to incorporate the three situation variables—social partner (“who”), location (“where”), and activity (“what”)—as moderators in the multilevel models. These variables were entered both as main effects and as interaction terms with the Level 1 situation perceptions in Model IV. This analysis generated 135 models (5 personality dimensions × 9 situation perceptions × 3 situation variables). As no hypotheses were made and to control for multiple testing within each moderation model, Bonferroni-Holm correction was applied across the k − 1 dummy-coded interaction terms of the respective situation variable.

#### Deviations From Pre-Registration

The research question originally formulated as conditional (“If differences emerge between settings, can these be attributed to situation variables in the ESM data?”) is now incorporated as a primary research question within the naturalistic dataset. Additionally, Typicality was not part of the pre-registered hypotheses; all analyses involving this dimension are exploratory. Finally, to ensure sufficient situational diversity, we included only participants with at least six valid assessments per wave and restricted analyses to the first 3 days.

## Results

### Descriptives

Table 5 presents descriptive statistics for the trait measures. Table 6 presents the correlations among aggregated Big Five states and DIAMONDS dimensions, as well as the ICCs for each variable. ICCs for the Big Five states ranged from .48 (Extraversion) to .58 (Agreeableness), indicating substantial within-person variability. Regarding situation perception dimensions, *Adversity* and *Deception* were the lowest-rated dimensions on average, whereas *Mating* perceptions exhibited the highest variability. The ICCs for situation perceptions ranged from .11 (*Duty* and *Sociality*) to .25 (*Deception*). Taken together, these findings indicate substantial within-person variation in both personality states and situation perceptions.

**Table 5.** Descriptive Statistics for Trait Measures in the Naturalistic Design

Construct	*M*	*SD*	1	2	3	4	*r*_trait-aggregated state_
1. Extraversion	3.01	0.74	-	​	​	​	.57***
2. Conscientiousness	3.43	0.72	.24***	-	​	​	.58***
3. Agreeableness	3.83	0.60	.13***	.22***	-	​	.51***
4. Neuroticism	2.90	0.78	−.38***	−.28***	−.25***	-	.63***
5. Openness	3.68	0.69	.20***	.02***	.10***	−.11***	.43***

*Note.* We report the average of all trait assessments at the start of each wave for the naturalistic data. *r* trait-state_agg_ indicates the correlation between the trait and the corresponding aggregated state across all measurement occasions. *p < .05, **p < .01, ***p < .001.

**Table 6.** Descriptive Statistics for Aggregated and Non-Aggregated State Measures in the Naturalistic Study

​	*M*	*SD*	1	2	3	4	5	6	7	8	9	10	11	12	13	Non-aggregated states ICC [95% CI]
Big Five states																
1. Extraversion	4.29	0.65	-	​	​	​	​	​	​	​	​	​	​	​	​	.48 [.46; .50]
2. Conscientiousness	4.70	0.80	.72***	​	​	​	​	​	​	​	​	​	​	​	​	.57 [.55; .58]
3. Agreeableness	5.23	0.74	.59***	.68***	-	​	​	​	​	​	​	​	​	​	​	.58 [.56; .59]
4. Neuroticism	3.50	0.85	−.63***	−.64***	−.67***	-	​	​	​	​	​	​	​	​	​	.53 [.52; .55]
5. Openness	4.25	0.66	.64***	.58***	.54***	−.48***	-	​	​	​	​	​	​	​	​	.52 [.50; .54]
Situation perception																
6. Duty	3.54	0.89	.05*	−.07**	−.06*	.21***	.02	-	​	​	​	​	​	​	​	.11 [.10; .12]
7. Intellect	2.93	0.82	.11***	.04	.02	.10***	.24***	.53***	-	​	​	​	​	​	​	.15 [.14; .16]
8. Adversity	1.35	0.47	−.15***	−.20***	−.33***	.31***	−.09***	.23***	.31***	-	​	​	​	​	​	.19 [.18; .20]
9. Mating	2.17	1.10	.17***	.17***	.19***	−.15***	.14***	.03	.08**	.16***	-	​	​	​	​	.23 [.22; .24]
10. Positivity	4.60	0.73	.53***	.57***	.61***	−.73***	.50***	−.19***	−.05*	−.25***	.20***	-	​	​	​	.18 [.17; .19]
11. Negativity	2.63	0.91	−.43***	−.47***	−.44***	.73***	−.31***	.39***	.31***	.50***	−.04	−.60***	-	​	​	.24 [.23; .25]
12. Deception	1.27	0.44	−.13***	−.20***	−.30***	.27***	−.08**	.25***	.32***	.82***	.13***	−.26***	.46***	-	​	.25 [.24; .26]
13. Sociality	4.02	0.87	.36***	.30***	.36***	−.22***	.24***	.17***	.16***	.11***	.43***	.32***	−.02	.06*	-	.11 [.10; .12]
14. Typicality	4.92	0.79	.06*	.13***	.15***	−.14***	.02	.09***	−.01	−.19***	−.03	.25***	−.16***	−.20***	.02	.17 [.16; .18]

*Note.* *p < .05, **p < .01, ***p < .001.

Additionally, we conducted descriptive analyses of the categorical situation variables. Table 7 summarizes the distribution of categorical situation cues, ICCs, and the number of distinct categories reported within each domain. Together, these distributions indicate that the dataset is dominated by everyday private and leisure contexts, with social and public situations occurring less frequently. This imbalance should be considered when interpreting the moderation effects of situation cues on the links between situation perceptions and Big Five states (Table 8).

**Table 7.** Descriptive Statistics for Categorical Situation Variables and Within-Person Variability

Situation variable	Category	*n*	Percentage %	ICC	Within-person distinct categories (*M* (*SD*), range)
Social partner	Alone	32,018	44.3	.17	3.62 (0.70), 1–4
	Close relations	19,814	27.4	.30	
	Work/study colleagues	10,849	15	.20	
	Other people	9,454	13.1	.18	
Location	Private spaces	45,964	63.6	.14	3.01 (0.84), 1–4
	Public/social spaces	21,476	29.7	.13	
	Outdoor settings	1,409	1.9	.21	
	Other places	3,362	4.7	.29	
Activity	Relaxation, leisure, or self-care	34,817	48.2	.07	4.02 (0.91), 1–5
	Responsibilities or work/study	21,343	29.6	.07	
	Social or interactive activities	9,858	13.6	.13	
	Travel	4,207	5.8	.26	
	Other activities	1,986	2.8	.25	

*Note.* Percentages are based on the total number of experience-sampling reports. Within-person values represent the average number of distinct categories each participant reported across measurement occasions, the within-person standard deviation (SD), and the range.

**Table 8.** Unstandardized Fixed L1 Effects of Within-Person Centered Situation Perceptions on Big Five States in the Naturalistic Data

	Extraversion			Conscientiousness			Agreeableness			Neuroticism			Openness		
	*b*	[CI]	*t*	*b*	[CI]	*t*	*b*	[CI]	*t*	*b*	[CI]	*t*	*b*	[CI]	*t*
D	0.02***	[0.02; 0.02]	10.18	0.02***	[0.01; 0.02]	8.89	−0.02***	[-0.02; −0.02]	−10.94	0.05†††	[0.05; 0.05]	24.49	−0.01**	[-0.01; 0.00]	−3.08
I	0.03***	[0.03; 0.04]	14.19	0.03***	[0.02; 0.03]	11.92	−0.01***	[-0.01; 0.00]	−3.27	0.04***	[0.03; 0.04]	14.95	0.02***	[0.02; 0.03]	10.38
A	−0.01***	[-0.02; −0.01]	−3.628	−0.03***	[-0.04; −0.02]	−7.7	−0.10***	[-0.10; −0.09]	−22.97	0.12***	[0.11; 0.13]	24.96	−0.04***	[-0.05; −0.04]	−11.75
M	0.02***	[0.02; 0.03]	10.35	0.01***	[0.01; 0.02]	4.64	0.04***	[0.03; 0.04]	16.69	−0.04***	[-0.05; −0.04]	−14.91	0.02***	[0.02; 0.02]	9.66
O	0.12***	[0.11; 0.12]	39.89	0.11***	[0.10; 0.11]	34.13	0.14***	[0.14; 0.15]	49.45	−0.21***	[-0.22; −0.20]	−62.28	0.13***	[0.12; 0.13]	45.4
N	−0.08***	[-0.09; −0.08]	−29.93	−0.09***	[-0.09; −0.08]	−29.28	−0.11***	[-0.12; −0.11]	−40.6	0.20***	[0.19; 0.21]	62.83	−0.10***	[-0.10; −0.09]	−35.31
De	−0.04***	[-0.05; −0.03]	−7.28	−0.05***	[-0.06; −0.04]	−9.66	−0.10***	[-0.11; −0.09]	−18.04	0.13***	[0.12; 0.14]	21.06	−0.06***	[-0.06; −0.05]	−10.98
S	0.07***	[0.06; 0.07]	37.44	0.05^†††^	[0.05; 0.05]	26.96	0.05^†††^	[0.04; 0.05]	27.8	−0.04^†††^	[-0.05; −0.04]	−21.00	0.03***	[0.03; 0.03]	19.5
T	0.01^†††^	[0.01; 0.02]	4.93	0.03^†††^	[0.02; 0.03]	9.81	0.01^†††^	[0.01; 0.02]	4.7	−0.02^†††^	[-0.02; −0.01]	−5.85	0.01^†††^	[0.01; 0.01]	3.91

*Note.* Model selection was based on the lowest AIC value. Coefficients refer to Model IV, which had the best model fit. Confidence intervals are two-tailed 95% CIs. *b* = estimate (unstandardized) effect; *t* = t-value. D = Duty, I = Intellect, A = Adversity, M = Mating, O = Positivity, N = Negativity, De = Deception, S = Sociality, T = Typicality.*p < .05, **p < .01, ***p < .001. For the few associations with no hypotheses, we used Bonferroni-Holm adjusted p-values to control the family-wise error rate.^†††^*p* Holm-adj. < .001, for the associations with no hypotheses.

### Co-Occurrence of Big Five States and Situation Perceptions

To address Research Question 1, we conducted a series of multilevel models (Models I–IV). Models that included random slopes for the associations between Big Five states and situation perceptions consistently showed the best fit, suggesting substantial individual differences in these relationships. Analyses of the relationships between Big Five personality states and situation perceptions mostly supported our hypotheses (32 out of 36), with some exceptions in which the relations went in the opposite direction. Next to the main analysis, we conducted additional multilevel models that controlled for all situation perceptions. The results remained largely consistent with the main analyses (see Table S5).

### The Objective Situation

As a first step toward addressing Research Question 2, we estimated separate multilevel models predicting DIAMONDS dimensions from each categorical situation variable (social partner, location, and activity). Results are presented in Table 9. We then introduced the three situation variables as Level 1 categorical predictors in our multilevel models (Model IV). For each situation variable, we included both main effects and interaction terms with DIAMONDS states. Notably, we initially attempted “maximal” models including random slopes for situation cues and the cue × perception interactions (Barr et al., 2013). However, many models failed to converge or were singular, reflecting sparse within-person variation at certain cue levels (e.g., some participants rarely reported specific locations/social partners). Following recommendations to adopt parsimonious structures when maximal models are not identifiable (e.g., Matuschek et al., 2017), we only retained random intercepts for persons and random slopes for the Level 1 situation perception predictor. Thus, the random slope structure was intentionally simplified to ensure model stability and interpretability.

**Table 9.** Zero-Order Associations between the Situation Variables and Situation Perceptions

​	Duty			Intellect			Adversity			Mating			Positivity			Negativity			Deception			Sociality			Typicality		
	*b*	CI	*t*	*b*	CI	*t*	*b*	CI	*t*	*b*	CI	*t*	*b*	CI	*t*	*b*	CI	*t*	*b*	CI	*t*	*b*	CI	*t*	*b*	CI	*t*
Model I: Social partner																											
RC: Alone	3.57***	[3.52, 3.61]	153.71	2.79***	[2.74, 2.83]	131.74	1.28***	[1.26, 1.31]	105.22	1.37***	[1.33, 1.42]	61.66	4.31***	[4.27, 4.34]	233.14	2.74***	[2.69, 2.78]	117.08	1.25***	[1.23, 1.27]	110.7	2.33***	[2.29, 2.36]	131.4	5.11***	[5.07, 5.15]	248.27
Close relations	−0.83***	[-0.86, −0.79]	−42.34	−0.21***	[-0.24, −0.18]	−12.74	0.11***	[0.09, 0.13]	12.49	2.65***	[2.62, 2.68]	174.84	0.78***	[0.76, 0.81]	57.22	−0.38***	[-0.41, −0.35]	−25.3	0.01·	[-0.00, 0.03]	1.73	3.07***	[3.04, 3.10]	195.5	−0.45***	[-0.48, −0.42]	−28.96
Work/study colleagues	2.15***	[2.10, 2.19]	88.6	1.50***	[1.46, 1.53]	73.86	0.22***	[0.19, 0.24]	19.94	0.16***	[0.12, 0.20]	8.56	−0.01	[-0.04, 0.02]	−0.64	0.22***	[0.18, 0.25]	11.68	0.11***	[0.10, 0.13]	13.12	3.35***	[3.31, 3.39]	171.4	0.39***	[0.35, 0.42]	19.9
Other people	−0.60***	[-0.65, −0.56]	−26.19	−0.05*	[-0.08, −0.01]	−2.39	0.06***	[0.04, 0.08]	5.58	0.33***	[0.30, 0.37]	18.61	0.56***	[0.52, 0.59]	34.56	−0.25***	[-0.29, −0.22]	−14.32	0.02·	[-0.00, 0.03]	1.94	2.76***	[2.72, 2.80]	148.8	−0.62***	[-0.65, −0.58]	−33.59
Model II: Location																											
RC: Private spaces	3.19***	[3.15, 3.24]	145.39	2.69***	[2.65, 2.73]	131.99	1.32***	[1.30, 1.35]	112.39	2.27***	[2.21, 2.32]	83.25	4.62***	[4.59, 4.66]	251.21	2.57***	[2.53, 2.62]	112.23	1.25***	[1.23, 1.27]	113.76	3.49***	[3.45, 3.53]	167.13	5.02***	[4.98, 5.06]	254.54
Public/social spaces	1.23***	[1.20, 1.27]	67.95	0.79***	[0.76, 0.82]	53.51	0.09***	[0.07, 0.10]	11.4	−0.37***	[-0.40, −0.34]	−23	−0.20***	[-0.22, −0.17]	−16.12	0.22***	[0.20, 0.25]	16.85	0.07***	[0.05, 0.08]	10.8	1.47***	[1.43, 1.50]	82.97	−0.03*	[-0.05, −0.00]	−1.97
Outdoor (nature)	−0.85***	[-0.96, −0.74]	−14.71	−0.06	[-0.15, 0.03]	−1.32	−0.04·	[-0.09, 0.01]	−1.69	0.13**	[0.03, 0.23]	2.58	1.00***	[0.93, 1.08]	25.77	−0.50***	[-0.58, −0.42]	−12	−0.04*	[-0.08, −0.00]	−2.06	1.29***	[1.18, 1.40]	22.89	−0.97***	[-1.06, −0.89]	−22.2
Other	−0.19***	[-0.26, −0.11]	−4.75	−0.03	[-0.09, 0.03]	−0.89	0.02	[-0.01, 0.05]	1.17	0.17***	[0.10, 0.23]	4.78	0.35***	[0.30, 0.40]	13.21	−0.08**	[-0.13, −0.02]	−2.77	−0.01	[-0.04, 0.02]	−0.67	1.48***	[1.40, 1.55]	38.78	−1.06***	[-1.12, −1.00]	−35.67
Model III: Activity																											
RC: Relaxation/leisure/self-care	2.60***	[2.57, 2.64]	131.7	2.33***	[2.29, 2.37]	117.08	1.27***	[1.25, 1.30]	105.83	2.30***	[2.25, 2.36]	83.54	4.84***	[4.80, 4.88]	256.21	2.37***	[2.33, 2.42]	102.16	1.23***	[1.21, 1.25]	110.3	3.64***	[3.59, 3.68]	163.79	4.91***	[4.87, 4.95]	243.47
Travel	−0.06	[-0.14, 0.02]	−1.38	−0.05	[-0.13, 0.02]	−1.42	0.05*	[0.01, 0.09]	2.37	−0.02	[-0.10, 0.07]	−0.42	−0.47***	[-0.54, −0.41]	−14.42	0.41***	[0.34, 0.48]	11.66	<.0001	[-0.03, 0.04]	0.21	0.53***	[0.43, 0.62]	10.68	−0.59***	[-0.67, −0.52]	−15.91
Responsibilities/work	2.94***	[2.91, 2.97]	188.47	1.68***	[1.65, 1.70]	118.81	0.13***	[0.11, 0.15]	16.52	−0.57***	[-0.60, −0.53]	−34.53	−0.72***	[-0.74, −0.70]	−58.02	0.63***	[0.61, 0.66]	47.32	0.07***	[0.06, 0.08]	11.12	0.53***	[0.49, 0.57]	28.36	0.45***	[0.42, 0.47]	31.6
Social interaction	0.15***	[0.11, 0.19]	7.38	0.55***	[0.51, 0.59]	29.55	0.23***	[0.21, 0.25]	22.2	0.24***	[0.20, 0.29]	11.25	0.01	[-0.02, 0.05]	0.84	0.26***	[0.22, 0.29]	14.6	0.10***	[0.09, 0.12]	12.29	1.52***	[1.47, 1.57]	61.66	−0.42***	[-0.46, −0.38]	−22.55
Other	0.70***	[0.64, 0.76]	23.7	0.33***	[0.27, 0.38]	12.18	0.06***	[0.03, 0.08]	3.68	−0.04	[-0.10, 0.02]	−1.33	−0.29***	[-0.34, −0.25]	−12.46	0.37***	[0.32, 0.42]	14.47	<.0001	[-0.02, 0.03]	0.25	0.15***	[0.08, 0.22]	4.34	−0.45***	[-0.50, −0.40]	−16.87

*Note.* Confidence intervals are two-tailed 95% CIs. *b* = estimate (unstandardized) effect; *t* = t-value. *p < .05, **p < .01, ***p < .001, two-tailed. D = Duty, I = Intellect, A = Adversity, M = Mating, O = Positivity, N = Negativity, De = Deception, S = Sociality, T = Typicality.

The situation variables were dummy-coded, with reference categories set as: *alone* (for social partner), *private spaces* (for location), and *relaxation* (for activity). Including these interactions increased the amount of explained variance in both random intercepts and slopes, with notable differences across Big Five dimensions and situation perceptions (see Table S7). Across all models, the inclusion of the social partner dummies explained the most additional variance, followed by location, with activity contributing the least. This pattern highlights the central role of social relations in shaping the connection between perceived situations and the expression of momentary personality states. Importantly, the explained variance in slopes was consistently higher than that in intercepts. Models for Conscientiousness and Extraversion exhibited the strongest increases in explained variance when situation cues were added, with up to 11.1% and 10.9% additional explained slope variance, respectively. Models for Agreeableness and Neuroticism showed consistent but smaller improvements (each up to ∼10.4%), whereas Openness models showed the weakest effects (maximum ≈ 6.5%). For situation perceptions, the strongest increases in explained slope variance were for the Typicality model, followed by the Duty and Intellect models.

Our results show systematic moderating effects of the situation variables on the within-person relationships between Big Five states and situation perceptions. Due to the large number of tested models (5 × 9 × 3 = 135), the complete set of interaction coefficients is reported in the Supplement (see Table S6). Of the 450 interaction contrasts tested across these models, 158 (35.1%) were significant after Bonferroni-Holm correction. Critically, the moderating influence of situation cues was not uniform across situation perceptions. The situation perceptions of Typicality, Duty, and Intellect showed the densest patterns of significant interaction coefficients across personality dimensions, indicating that the strength and direction of the association between these situation perceptions and personality states depended considerably on the situation cues present. By contrast, Positivity and Negativity exhibited weaker and more stable slopes across cues, suggesting that these situation perceptions relate to personality states in a relatively uniform manner across social, spatial, and activity contexts.

**Figure 3.**
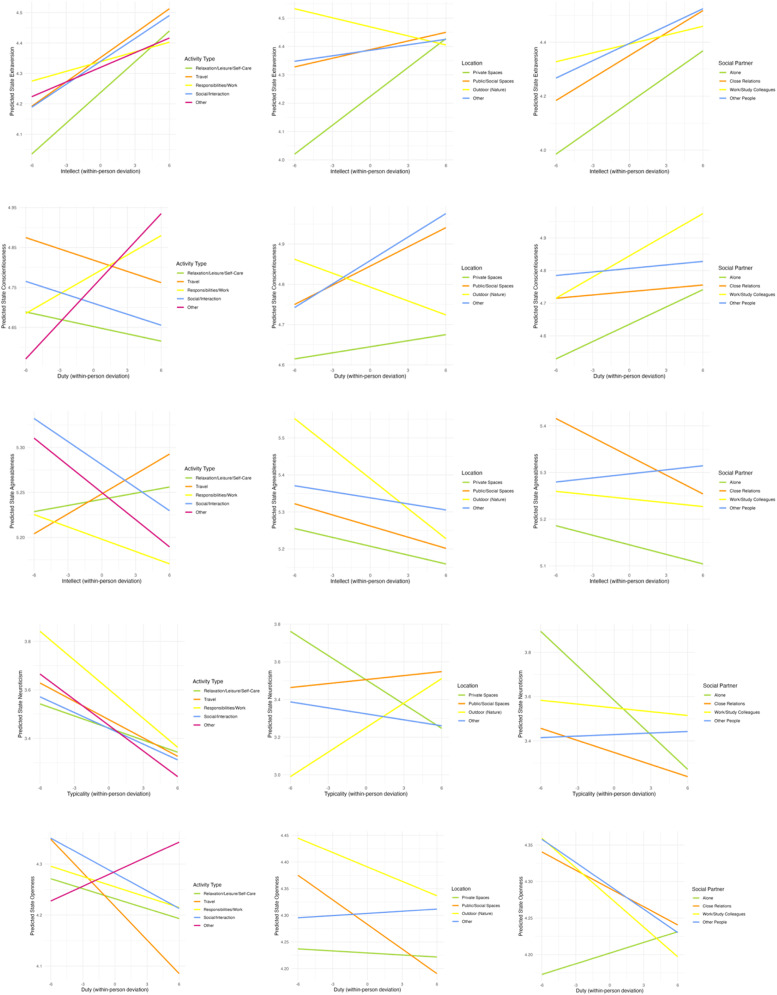
Examples of situation variables as moderators of the relations between Big Five states and situation perceptions *Note.* Plots illustrate selected cross-level interactions between DIAMONDS situation perceptions (*x*-axes) and Big Five personality states (*y*-axes) as moderated by categorical situation variables (colors)

Exemplary interactions for each Big Five dimension are presented in Figure 3. Several of these interactions were disordinal, indicating not only differences in strength but also reversals in the direction of associations. For instance, the relationship between Extraversion states and perceived Intellect was stronger when others were present and attenuated when participants were alone, suggesting that intellectual engagement is more readily perceived in social company. The figure also illustrates the interaction between Conscientiousness states and perceived Duty moderated by activity type: during relaxation, higher perceived Duty was associated with *lower* Conscientiousness states, whereas during responsibilities and work, the association became positive—a crossover suggesting that duty-related cues carry opposing meanings depending on activity context. For Neuroticism, the association with perceived Typicality reversed as a function of location: in private spaces, higher Neuroticism was negatively related to Typicality, whereas this association became positive in public and outdoor settings. For Agreeableness, the relationship with perceived Typicality was moderated by social partner, showing the highest explained slope variance in this cue category. Similarly, for Openness, the Typicality × social partner interaction demonstrated the strongest moderation among all cue-perception combinations for this trait. Taken together, these examples highlight the cue-sensitive nature of state–situation perception links and demonstrate the value of modeling interactions to capture how personality expression dynamically adjusts to specific situational features.

### Discussion

Study 2 extended the laboratory findings to everyday life, where both contact and construal varied freely. The central finding was that situation cues—social partner, location, and activity—moderated the within-person associations between Big Five states and situation perceptions. Though statistically significant, many of these associations were small to medium in magnitude (*b* = 0.01–0.21 on a 1–7 scale). The situation cue moderations primarily affected slopes rather than intercepts, indicating that cues shaped when and under which circumstances perception–state links were stronger or weaker, rather than shifting stable between-person differences. The moderation was not uniform across DIAMONDS dimensions: personaity state associations with Typicality, Duty, and Intellect showed the strongest cue-dependence, whereas relations including Positivity and Negativity remained comparatively stable across contexts. Among the situation cues, social partner exerted the strongest moderating influence, followed by location, with activity contributing the least—underscoring the centrality of the immediate social context for momentary personality expression.

These findings bear directly on the contact–construal distinction. If associations between personality states and situation perceptions were driven purely by construal—that is, by how individuals subjectively interpret situations independently of objective features—these associations should be largely uniform across situation cues. Instead, the associations shifted systematically depending on who was present, where participants were, and what they were doing. This suggests that objective situation features shape not only which perceptions arise but also how those perceptions relate to personality states. In other words, part of the perception–state co-occurrences observed in naturalistic data reflect situation contact layered on top of construal—consistent with the theoretical distinction between contact and construal outlined in the introduction (e.g., Murray, 1938; Rauthmann et al., 2015b).

## Comparison of Study 1 and Study 2

**Figure 4.**
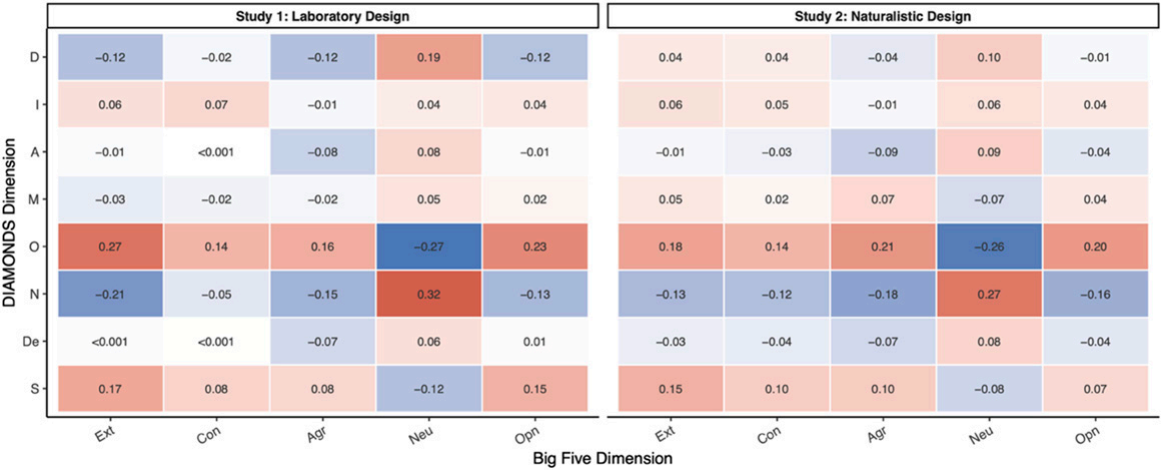
Standardized effect sizes across DIAMONDS dimensions and Big Five personality states in the laboratory and naturalistic studies. *Note.* Cells display standardized regression coefficients. D = Duty, I = Intellect, A = Adversity, M = Mating, O = Positivity, N = Negativity, De = Deception, S = Sociality. Color intensity reflects effect magnitude; blue = positive associations, red = negative associations

To directly compare effect sizes across the two study designs, we correlated the standardized regression coefficients (presented in Tables S7 and S8) for all 40 DIAMONDS × Big Five combinations common to both studies. Overall, effect sizes were substantially correlated (*r* = .89), indicating broad cross-study replication. Moreover, mean absolute effect magnitude was virtually identical across studies (*M|β|* = .095 vs. .089), indicating that the greater number of significant associations in the naturalistic study primarily reflects its considerably larger sample rather than systematically larger effects. Crucially, however, convergence was not uniform across DIAMONDS dimensions. For most dimensions—Intellect, Adversity, Positivity, Negativity, and Sociality—the direction and magnitude of associations were nearly identical across laboratory and naturalistic contexts. In contrast, the Duty and Mating dimensions showed pronounced divergence, with several associations reversing sign across studies. Thus, despite the overall comparability of effect sizes across studies, the divergence observed for specific dimensions is theoretically meaningful rather than incidental and can be interpreted in light of the specific situation contact of the laboratory design. Figure 4 provides a visual overview of standardized effect sizes across both studies.

## General Discussion

This study investigated how situation contact and construal influence personality state expressions. The conceptual distinction between contact and construal is well established—following from frameworks that distinguish subjective from objective situational features (e.g., Murray, 1938; Rauthmann et al., 2015b). Within a unified perception framework (DIAMONDS) and across two complementary study designs (laboratory vs naturalistic), the present work provides one of the first direct empirical demonstrations of what changes when contact is constrained versus free to vary: across both designs, the overall pattern of standardized Big Five state–situation perception effect sizes was largely consistent in shape and magnitude, supporting the view that situation construal is the primary driver of person–situation co-occurrences. At the same time, situation contact acted as a meaningful moderator. Notably, it produced several disordinal interactions in the naturalistic data in which the direction—not merely the strength—of associations shifted across social partners, locations, or activities. These findings carry important implications for understanding momentary personality expression.

### Person–Situation Co-Occurrences

The primary aim of this research was to examine within-person co-occurrences between personality states and situation perceptions across two complementary designs: one that experimentally constrains situation contact, and one that allows it to vary naturally and records the specific situation cues encountered. Our findings demonstrate substantial and meaningful associations between Big Five states and situation perceptions in both laboratory and naturalistic designs. Table 10 provides an overview of the hypotheses supported across both studies.

**Table 10.** Hypothesized and Observed Relationships Between Big Five States and Situation Perceptions

Big Five dimension	​	D	I	A	M	O	N	De	S	T
Extraversion										
​	Hypothesized	-	-	-	+	+	-	-	+	/
​	Laboratory	-	+*	​	​	+	-	​	+	​
​	Naturalistic	+*	+*	-	+	+	-	-	+	+
Conscientiousness	​	​	​	​	​	​	​	​	​	​
​	Hypothesized	+	+	-	-	+	-	-	/	/
​	Laboratory	​	+	​	​	+	-	​	+	​
​	Naturalistic	+	+	-	+*	+	-	-	+	+
Agreeableness		​	​	​	​	​	​	​	​	​
​	Hypothesized	-	-	-	+	+	-	-	/	/
​	Laboratory	-	​	-	​	+	-	-	+	​
​	Naturalistic	-	-	-	+	+	-	-	+	+
Neuroticism		​	​	​	​	​	​	​	​	​
​	Hypothesized	/	+	+	-	-	+	+	/	/
​	Laboratory	+	​	+	+*	-	+	+	-	​
​	Naturalistic	+	+	+	-	-	+	+	-	-
Openness		​	​	​	​	​	​	​	​	​
​	Hypothesized	+	+	-	+	+	-	-	+	/
​	Laboratory	-*	+	​	​	+	-	​	+	​
​	Naturalistic	-*	+	-	+	+	-	-	+	+

*Note.* + = positive relationship; - = negative relationship; / = no hypothesized relationship; * indicates a significant relationship in the opposite direction than hypothesized. No *Typicality* associations were estimated in the experimental data, as this situation perception was not assessed. D = Duty, I = Intellect, A = Adversity, M = Mating, O = Positivity, N = Negativity, De = Deception, S = Sociality, T = Typicality.

In the laboratory paradigm, 20 of our 36 hypothesized associations received empirical support. The associations that emerged were concentrated in dimensions readily evoked by the structured social interaction of the lab—particularly Positivity, Negativity, and Sociality—whereas dimensions requiring more varied situation contact, such as Mating, Adversity, and Deception, showed fewer significant associations, consistent with the constrained situational range of the design. Different laboratory paradigms—such as those incorporating mixed-sex groups, adversarial or competitive tasks, or stranger-interaction formats with more affectively loaded stimuli—would likely generate greater variability in these dimensions and may enable detection of a broader set of associations. In other words, which associations emerge in a laboratory setting is itself informative about the type of situation contact the design affords.

In the naturalistic design, 32 of 36 hypothesized associations reached significance. This higher confirmation rate should not be interpreted as evidence for systematically stronger effects in everyday life: the cross-study comparison of standardized effect sizes showed that, for most DIAMONDS dimensions, standardized coeffiecients were comparable in magnitude across designs. Thus, the higher number of significant associations in the naturalistic design is best attributed to the greater statistical power of the larger sample. More noteworthy, four associations contradicted the hypothesized directions, which had been derived from previous naturalistic findings (see Table 10). Our results showed that all associations that contradicted our hypotheses were, at least to some extent, moderated by situation cue variables—social partners, location, or activity—that participants reported. For instance, people’s higher Extraversion states were positively associated with their perceptions of Intellect and Duty, contradicting our hypothesized negative relationships. The positive association with perceptions of Intellect suggests that intellectually stimulating situations can be perceived as engaging and are thereby accompanied by higher extraverted states. However, this association was moderated by participants’ reported location, such that the effect was weaker in outdoor settings compared to private spaces, indicating that the association depends upon the situation contact a person is experiencing. Similarly, the positive association between Extraversion and Duty perceptions was moderated by the type of activity. Specifically, work-related contexts were associated with stronger positive associations between Duty and Extraversion than the reference category (relaxation). In contrast, social interaction contexts reversed the pattern: here, the link between Duty perceptions and Extraversion states was not only weaker compared to the relaxation category but also showed a negative relationship. This reversal is theoretically interpretable through a goal-conflict lens: social situations typically afford extraverted behavior because their inherent goal is connection and shared experience, whereas Duty construals entail the goal of task completion. When Duty is construed within a social interaction context, the situation cues and the construal impose conflicting goals—connecting versus completing—requiring individuals to downregulate Extraversion to meet task demands. In work contexts, by contrast, cues and construal converge on the same goal, allowing Duty and Extraversion to align. This pattern suggests that situation contact (the cues encountered) may functionally precede construal by shaping which behavioral tendencies a given construal can afford. These disordinal interactions reveal that how situation perceptions and personality expressions relate can depend fundamentally on the situation encountered. The reversal of the Duty–Extraversion association across activities illustrates this point: the same state–perception link can switch sign depending on the situational context, meaning that an aggregate estimate across all activities would obscure—or even cancel out—a relationship that is reliable within specific contexts.

### The “Objective” Situation

Although the standardized effect sizes were broadly comparable across designs, the few dimensions that diverged—most notably Duty and Mating—did so in ways consistent with the specific situational affordances of each setting. This cross-study divergence provides an initial indication that situation contact shapes which person–situation associations emerge. The Duty and Mating dimensions are particularly instructive: in the laboratory, participants were placed in same-sex discussion groups with a pre-assigned topic, meaning both the social composition and the task demands were externally imposed rather than self-selected. In everyday life, by contrast, duty-relevant situations are at least partly chosen, and social interactions are mostly not constrained to same-sex partners, allowing for a broader and more personally relevant range of contact. The divergence for precisely these two dimensions suggests that their associations with personality states are especially sensitive to whether situation contact is externally constrained or freely navigated—and that averaging across contact conditions without accounting for this distinction risks obscuring theoretically meaningful variation.

To obtain a more finely differentiated understanding of the person–situation relations in the laboratory design, we compared patterns across manipulation conditions. Splits by manipulation condition (affiliative vs. controversial discussions) indicated that most associations replicated, with only modest shifts in strength. This consistency is conceivable given that the broader interaction frame—same partners, online format, and task structure—remained constant across sessions.

In the naturalistic design, situation contact could be differentiated more granularly—by indicators of social partners, locations, and activities. Of the 450 interaction contrasts tested across these models, 158 (35.1%) indicated that situation variables meaningfully moderated a substantial portion of person–situation associations. In other words, although many relationships remained robust across situation variables, others shifted—and in some cases reversed—when taking into account who participants were with (social partner), where they were (location), or what they were doing (activity). These shifts were most pronounced for personality state relations with the situation perceptions of Typicality, Duty, and Intellect, and less so for relations with more affective dimensions such as Positivity and Negativity. This suggests a domain-specificity account: evaluative and quickly processed dimensions such as Positivity and Negativity (Rauthmann, 2016) carry relevance across virtually all objective situations and thus yield more consistent person–situation associations, whereas cognitively loaded dimensions such as Duty and Intellect are most activated in specific situational contexts (e.g., task-oriented or professional settings), producing associations that vary more markedly across cues. Together, these findings underscore that situation contact does not uniformly moderate all person–situation relations; its influence is strongest for dimensions inherently tied to specific situational demands. This is consistent with Matz and Harari (2021) who showed that Big Five states vary systematically across everyday locations, with public and social spaces associated with higher Extraversion and lower Neuroticism. Experience-sampling data that lack explicit information about the objective situation may therefore offer only a partial picture of person–situation relations, as the underlying variability in situation contact remains unaccounted for.

### Individual Differences in Person–Situation Relations

A notable finding across both studies is that person–situation associations were not uniform: individuals differed in the strength and direction of the links between situation perceptions and Big Five states. In the laboratory data, models including random slopes provided the best fit for the majority of associations (28 of 40), indicating detectable individual differences in person–situation relations even under controlled conditions. The 12 associations for which fixed-effects models were preferable were for situation perceptions of Adversity, Mating, and Deception—dimensions for which the structured, same-sex video-conference format constitutes a relatively strong situation (Meyer et al., 2014; Mischel, 1977) with limited trait relevance (Lievens et al., 2018), leaving little room for between-person variability to emerge. In the naturalistic data, random slopes were consistently preferred across all 45 associations, a pattern consistent with ESM research demonstrating individual differences in situation characteristic–state contingencies (Kuper et al., 2022; Mischel & Shoda, 1995).

Crucially, the present results go beyond documenting that such individual differences exist. The situation cue analyses in Study 2 suggest that a meaningful portion of the interindividual variability in contingencies can be traced to specific features of the situation context—who was present, where participants were, and what they were doing. People differed not only in how they responded to particular situational features—consistent with the if–then logic of cognitive–affective processing accounts (Fleeson & Jayawickreme, 2015, 2025; Mischel & Shoda, 1995)—but also in which situations they encountered in the first place, likely reflecting personality-driven self-selection into situations (Rauthmann et al., 2015b). This introduces a confound between contact and construal at the between-person level that the present design cannot fully disentangle.

### Limitations and Future Directions

The present research has several limitations that should be acknowledged. Most importantly, the situation cues assessed in the naturalistic ESM design—social partner, location, and activity—were not fully objective, as they relied on participants’ self-reports. Although these structured cue reports represent a more direct operationalization of situation contact than the perception-based rater comparisons used in prior work, they inevitably reflect individual differences in how situations are categorized—for instance, one participant may label “working from home” as a private and solitary setting, whereas another may construe it as part of a social or professional context. Additionally, these cues were recoded into broad categories, which may obscure finer distinctions. Future research should address this by incorporating sensor-based or observer-rated assessments of situation contexts, which would more directly capture situation contact without relying on participants’ potentially construal-influenced descriptions. A related limitation concerns the degree to which situation contact was truly held constant in the laboratory study. Although the session protocol was carefully designed to standardize the interactional context—through fixed discussion prompts, moderator oversight, and regular questionnaire interruptions—live group interactions are inherently dynamic, and full contact equivalence is unattainable. Group dynamics inevitably vary: dominant participants can steer conversations in unintended directions, interpersonal friction or rapport can emerge unexpectedly, and the pace and depth of discussion can differ. The degree to which we could truly hold situation contact constant should therefore be understood as an approximation rather than an achieved standard. Thus, the two designs each entail inherent trade-offs: the laboratory setting provided control at the cost of ecological variation in situation contact, whereas the naturalistic design captured real-world variation but introduced potential confounds due to unobserved situational heterogeneity. Future research could address this by combining both approaches in hybrid paradigms that experimentally manipulate situation cues within naturalistic contexts, enabling stronger causal inference about when and why specific person–situation associations emerge.

Another limitation concerns measurement: the laboratory and naturalistic studies employed different instruments and different numbers of assessments for the Big Five states. A related concern is that the Personality State Scale (Ringwald et al., 2022) was developed for general daily-life sampling, and some item content may have a limited operating range in a constrained video-conference group format with pre-defined topics, where opportunities for, for example, physical activity or task organization are restricted. Future laboratory studies should adopt or adapt state items whose content is fully within the behavioral range afforded by the experimental setting. Moreover, despite the reduced perceptions of Mating in the laboratory design, same-sex group compositions do not preclude mating-relevant dynamics; future work could vary group gender composition to examine whether this alters Mating-related person–situation associations. Lastly, the group interactions were conducted online. Although this approach offered logistical advantages and represents a common modern form of interacting, online settings may differ in important ways from face-to-face encounters—for instance, nonverbal cues are less salient, and the sense of co-presence may be diminished. Future studies should compare person–situation dynamics across in-person and online interaction formats to assess the generalizability of the present findings.

## Conclusion

Understanding how personality unfolds in everyday life requires simultaneously considering both the person and the situation. Whereas the conceptual distinction between situation contact and situation construal has long been recognized (Murray, 1938), empirical work that directly examines the consequences of separating these two sources within a unified perceptual framework has remained scarce. The present research addresses this gap, providing one of the first systematic, multi-design empirical examinations of how constraining versus freeing situation contact changes the nature of observed person–situation associations.

Three findings stand out as having direct empirical value beyond what existing theory already anticipates. First, a cross-study comparison of standardized effect sizes showed that the overall pattern of person–situation associations was broadly consistent across the controlled and naturalistic designs, pointing to a substantial role for construal in shaping these associations. This consistency is noteworthy given that the two designs differ fundamentally in the situation contact they afford—yet it was not uniform. For dimensions whose expression depends on specific situational affordances, particularly Duty and Mating, effect sizes diverged descriptively—consistent with the view that the structured same-sex discussion format constrained the situational affordances on which these dimensions depend, while leaving conditions for affective and evaluative dimensions such as Positivity, Negativity, and Sociality largely intact. Second, moderation analyses using the naturalistic data showed that situation contact does more than attenuate associations; it can qualitatively reshape them. Several person–situation links reversed direction across social partners, locations, or activities, demonstrating that aggregating across situation contexts may yield misleading estimates of the associations between personality states and situation perceptions. Contact, then, is not mere noise to be controlled away but a boundary condition that specifies which person–situation associations emerge in a given context. Third, individual differences in person–situation associations can be partly explained by who was present, where participants were, and what they were doing—identifying a concrete set of situation cues that account for some of the variability in why person–situation relations differ across individuals.

Together, these findings carry direct implications for how we measure, analyze, and interpret person–situation relations. Experience-sampling studies that do not record situation cue information may conflate construal-driven and contact-driven variance, potentially yielding estimates that are difficult to replicate across samples and settings with different situational compositions. More broadly, the present work suggests that knowing *which* situation a person enters may not merely be a covariate to control for—it may also help explain the observed associations between personality states and perceptions of situations.

## Supplemental Material

Supplemental Material - Construal and Contact in Person–Situation Relations: Evidence from Controlled and Naturalistic Designs Supplemental Material for Construal and Contact in Person–Situation Relations: Evidence from Controlled and Naturalistic Designs by Sophie C. Bauditz, Fabian Gander, Alexander Grob, Alex Christoph Traut, Maximiliane Uhlich, Ursula Hess, Matthias Ziegler in European Journal of Personality.

## Note

1. All hypotheses, data preparation steps, and analytic procedures were pre-registered (https://osf.io/cwrus/). The data needed to reproduce the results will be made publicly available on the OSF following publication of other manuscripts based on the same dataset. The study designs were approved by the ethics committee of the Deutsche Gesellschaft für Psychologie (approval no. MZ 012017_amd_052015_2) and Institutional Review Board of the Department of Psychology at the University of Basel, Switzerland (approval no. 003-16-1).
