# Supplementary material for: Construal and Contact in Person–Situation Relations: Evidence from Controlled and Naturalistic Designs
Source: Eur J Pers. 2026 May 22;40(5):1309–32. doi: 10.1177/08902070261449352 (PMC13485120; doi:10.1177/08902070261449352)
Supplement: Supplementary file 1 [file sj-pdf-1-erp-10.1177_08902070261449352.pdf]

Supplementary Findings

Table S1

Unstandardized Fixed L1 Effects of Within-Person Centered Situation Perceptions on Big Five States in ***Affiliative*** *Group Situations*

	Extraversion			Conscientiousness			Agreeableness			Neuroticism			Openness		
	b	[CI]	*t*	*b*	[CI]	*t*	*b*	[CI]	*t*	*b*	[CI]	*t*	*b*	[CI]	*t*
D	-0.16***	[-0.21; -0.10]	-4.11	-0.04	[-0.08; 0.00]	-1.3	-0.13***	[-0.17; -0.10]	-5.65	0.2***	[0.15; 0.25]	6.29	-0.12***	[-0.15; -0.07]	-4.23
I	0.03[a]	[-0.02; 0.09]	1.15	0.05	[0.02; 0.10]	1.97	<0.001	[-0.04; 0.04]	0.16	0.05[a]	[-0.00; 0.10]	1.86	0.04	[0.00; 0.10]	1.37
A	-0.07[a]	[-0.18; 0.03]	-1.34	<0.001[a]	[-0.09; 0.08]	-0.03	-0.15**[a]	[-0.22; -0.07]	-3.94	0.16**[a]	[0.06; 0.26]	3.03	-0.03[a]	[-0.12; 0.05]	-0.76
M	-0.06[a]	[-0.13; 0.01]	-1.63	-0.02[a]	[-0.07; 0.04]	-0.64	-0.02[a]	[-0.07; 0.03]	-0.69	0.11**[a]	[0.05; 0.18]	3.34	0.04[a]	[-0.02; 0.09]	1.2
O	0.36***	[0.30; 0.41]	9.4	0.16***	[0.11; 0.21]	5.2	0.17***	[0.13; 0.22]	6.28	-0.33***	[-0.38; -0.27]	-9.9	0.27***	[0.23; 0.32]	9.89
N	-0.28***[a]	[-0.33; -0.23]	-10.26	-0.07	[-0.10; -0.02]	-2.4	-0.14***	[-0.17; -0.10]	-5.87	0.37***[a]	[0.33; 0.42]	15.5	-0.16***	[-0.20; -0.11]	-6.32
De	-0.04[a]	[-0.17; 0.09]	-0.61	-0.04[a]	[-0.14; 0.06]	-0.73	-0.15**[a]	[-0.24; -0.06]	-3.25	0.13[a]	[0.01; 0.25]	2.07	<0.001[a]	[-0.11; 0.11]	0.03
S	0.16***	[0.12; 0.21]	6.34	0.07*	[0.03; 0.10]	2.87	0.07**[a]	[0.04; 0.10]	4.11	-0.12***	[-0.17; -0.08]	-4.17	0.14***[a]	[0.10; 0.18]	7.38

Note. Model selection was based on the lowest AIC value. Coefficients refer to Model IV, except for models with subscript a, where Model III is reported as the AIC indicated better fit. D = Duty, I = Intellect, A = Adversity, M = Mating, O = Positivity, N = Negativity, De = Deception, S = Sociality. Confidence intervals are two-tailed 95% CIs. b = estimate (unstandardized) effect; t = t-value. *p < 0.05 **, p < 0.01, ***p < 0.001. Significance stars reflect Bonferroni-Holm-adjusted p-values.

Table S2

Unstandardized Fixed L1 Effects of Within-Person Centered Situation Perceptions on Big Five States in ***Controversial*** *Group Situations*

	Extraversion			Conscientiousness			Agreeableness			Neuroticism			Openness		
	b	[95% CI]	*t*	*b*	[95% CI]	*t*	*b*	[95% CI]	*t*	*b*	[95% CI]	*t*	*b*	[95% CI]	*t*
D	-0.15***	[-0.20; -0.09]	-4.01	-0.05	[-0.09; 0.00]	-1.47	-0.11***[a]	[-0.15; -0.07]	-5.48	0.23***	[0.19; 0.29]	7.13	-0.14***	[-0.19; -0.10]	-4.52
I	0.08*	[0.03; 0.14]	2.59	0.07*	[0.02; 0.11]	2.87	-0.03[a]	[-0.06; 0.01]	-1.37	0.02[a]	[-0.03; 0.07]	0.22	0.04	[-0.01; 0.08]	1.42
A	0.04[a]	[-0.05; 0.13]	1.08	0.03[a]	[-0.05; 0.10]	0.77	-0.12*	[-0.17; -0.05]	-2.93	0.16*	[0.06; 0.22]	2.94	0.02[a]	[-0.06; 0.09]	0.44
M	-0.03[a]	[-0.11; 0.06]	-0.65	-0.02[a]	[-0.09; 0.05]	-0.63	-0.01	[-0.04; 0.07]	-0.4	0.02	[-0.07; 0.08]	0.62	0.01[a]	[-0.06; 0.08]	0.2
O	0.33***	[0.26; 0.38]	8.86	0.16***[a]	[0.11; 0.22]	6.23	0.19***	[0.15; 0.23]	7.4	-0.32***	[-0.37; -0.26]	-9.31	0.22***	[0.16; 0.26]	8.23
N	-0.2***	[-0.24; -0.12]	-5.04	-0.04	[-0.08; 0.03]	-1.05	-0.15***	[-0.20; -0.11]	-5.69	0.34***	[0.29; 0.39]	10.37	-0.12***[a]	[-0.17; -0.07]	-4.57
De	-0.01[a]	[-0.14; 0.13]	-0.11	0.02a	[-0.09; 0.14]	0.42	-0.2*	[-0.24; -0.05]	-2.85	0.13[a]	[0.00; 0.25]	1.99	0.01[a]	[-0.10; 0.13]	0.2
S	0.15***	[0.10; 0.19]	4.65	0.07*	[0.03; 0.11]	2.8	0.06**[a]	[0.03; 0.09]	3.5	-0.06	[-0.11; -0.02]	-2.21	0.12***	[0.08; 0.16]	4.52

Note. Model selection was based on the lowest AIC value. Coefficients refer to Model IV, except for models with subscript a, where Model III is reported as the AIC indicated better fit. D = Duty, I = Intellect, A = Adversity, M = Mating, O = Positivity, N = Negativity, De = Deception, S = Sociality. Confidence intervals are two-tailed 95% CIs. *b* = estimate (unstandardized) effect; *t* = t-value. *p < 0.05 **, p < 0.01, ***p < 0.001. Significance stars reflect Bonferroni-Holm-adjusted p-values.

Table S3

Standardized Within-Person Regression Coefficients (β) for Big Five State–DIAMONDS Associations, Separately for Affiliative (Sessions 1–3) and Controversial (Sessions 4–6) Conditions in Study 1 (Profile Correlation r = .97)

Personality	DIAMONDS	Affiliative β [95% CI]	Controversial β [95% CI]	Δβ
Extraversion	Duty	−0.158 [−0.232, −0.083]***	−0.127 [−0.189, −0.065]***	−0.030
	Intellect	0.026 [−0.034, 0.085]	0.076 [0.018, 0.133]*	−0.050
	Adversity	−0.036 [−0.088, 0.017]	0.034 [−0.028, 0.097]	−0.070
	Mating	−0.041 [−0.095, 0.012]	−0.011 [−0.065, 0.044]	−0.031
	Positivity	0.307 [0.244, 0.371]***	0.247 [0.192, 0.302]***	0.060
	Negativity	−0.248 [−0.297, −0.200]***	−0.153 [−0.213, −0.094]***	−0.095
	Deception	−0.025 [−0.082, 0.031]	−0.004 [−0.062, 0.054]	−0.021
	Sociality	0.185 [0.128, 0.242]***	0.147 [0.086, 0.208]***	0.038
Conscientiousness	Duty	−0.037 [−0.093, 0.019]	−0.042 [−0.098, 0.013]	0.005
	Intellect	0.056 [0.000, 0.112]	0.072 [0.023, 0.122]**	−0.017
	Adversity	−0.000 [−0.045, 0.045]	0.033 [−0.022, 0.087]	−0.033
	Mating	−0.014 [−0.057, 0.028]	−0.012 [−0.055, 0.030]	−0.002
	Positivity	0.148 [0.093, 0.204]***	0.135 [0.081, 0.189]***	0.014
	Negativity	−0.065 [−0.119, −0.012]*	−0.030 [−0.085, 0.025]	−0.035
	Deception	−0.034 [−0.087, 0.020]	0.005 [−0.050, 0.061]	−0.039
	Sociality	0.082 [0.026, 0.137]**	0.076 [0.024, 0.128]**	0.006
Agreeableness	Duty	−0.141 [−0.190, −0.092]***	−0.101 [−0.143, −0.059]***	−0.040
	Intellect	0.004 [−0.045, 0.053]	−0.027 [−0.072, 0.018]	0.031
	Adversity	−0.082 [−0.125, −0.040]**	−0.080 [−0.134, −0.026]**	−0.003
	Mating	−0.022 [−0.068, 0.023]	−0.010 [−0.058, 0.038]	−0.012
	Positivity	0.162 [0.110, 0.213]***	0.168 [0.123, 0.213]***	−0.006
	Negativity	−0.143 [−0.190, −0.095]***	−0.136 [−0.183, −0.090]***	−0.006
	Deception	−0.069 [−0.112, −0.026]**	−0.088 [−0.148, −0.027]**	0.019

	Sociality	0.083 [0.035, 0.130]**	0.075 [0.034, 0.117]**	0.008
Neuroticism	Duty	0.188 [0.130, 0.245]***	0.198 [0.144, 0.253]***	−0.011
	Intellect	0.051 [−0.006, 0.109]	0.017 [−0.040, 0.073]	0.035
	Adversity	0.084 [0.030, 0.138]**	0.091 [0.031, 0.151]**	−0.007
	Mating	0.077 [0.023, 0.130]*	0.018 [−0.039, 0.074]	0.059
	Positivity	−0.272 [−0.327, −0.218]***	−0.249 [−0.301, −0.197]***	−0.023
	Negativity	0.318 [0.271, 0.364]***	0.276 [0.224, 0.328]***	0.041
	Deception	0.063 [0.006, 0.120]*	0.071 [0.005, 0.136]*	−0.008
	Sociality	−0.132 [−0.193, −0.070]***	−0.068 [−0.127, −0.009]*	−0.064
Openness	Duty	−0.125 [−0.184, −0.067]***	−0.121 [−0.174, −0.068]***	−0.004
	Intellect	0.041 [−0.018, 0.099]	0.039 [−0.015, 0.094]	0.001
	Adversity	−0.019 [−0.066, 0.027]	0.011 [−0.048, 0.070]	−0.031
	Mating	0.018 [−0.034, 0.069]	−0.000 [−0.049, 0.049]	0.018
	Positivity	0.249 [0.199, 0.300]***	0.180 [0.137, 0.223]***	0.069
	Negativity	−0.156 [−0.204, −0.108]***	−0.105 [−0.156, −0.054]***	−0.051
	Deception	−0.006 [−0.055, 0.043]	0.001 [−0.053, 0.055]	−0.007
	Sociality	0.167 [0.115, 0.219]***	0.134 [0.077, 0.191]***	0.032

Note. β = standardised within-person regression coefficient; CI = confidence interval; Δβ = difference (Affiliative − Controversial). *$p < .05$, **$p < .01$, ***$p < .001$ (two-tailed). Profile correlation of the 40 standardised coefficients across conditions: $r = .97$.

Figure S1

Scatter Plot of Standardized Within-Person Regression Coefficients (β) for Big Five State–DIAMONDS Associations in the Affiliative (Sessions 1–3) Versus Controversial (Sessions 4–6) Conditions of Study 1

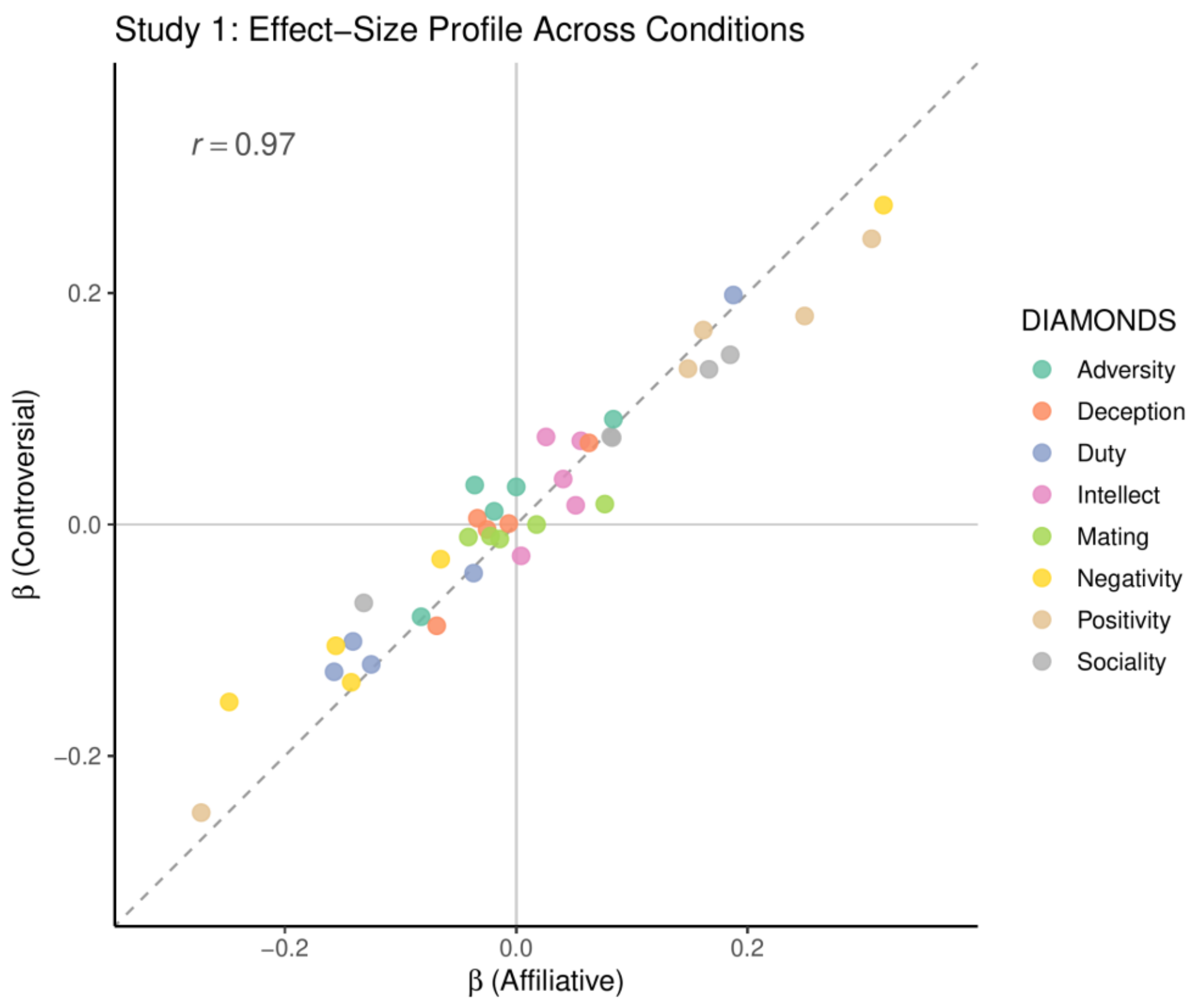

Table S4

Unstandardized Fixed L1 Effects of Within-Person Centered Situation Perceptions on Big Five States in the ***Laboratory Design Controlling for All Situation Perceptions***

	Extraversion			Conscientiousness			Agreeableness			Neuroticism			Openness		
	b	[CI]	*t*	*b*	[CI]	*t*	*b*	[CI]	*t*	*b*	[CI]	*t*	*b*	[CI]	*t*
D	-0.063*	[-0.112; -0.014]	-2.52	-0.004	[-0.049; 0.041]	-0.18	-0.051**	[-0.084; -0.018]	-3.02	0.081***	[0.040; 0.122]	3.87	-0.068**	[-0.108; -0.028]	-3.31
I	0.058*	[0.010; 0.106]	2.38	0.051**	[0.015; 0.088]	2.77	0.017	[-0.015; 0.049]	1.06	-0.014	[-0.052; 0.024]	-0.70	0.043*	[0.004; 0.082]	2.14
A	0.079	[-0.004; 0.163]	1.86	0.059	[-0.006; 0.124]	1.78	-0.028	[-0.085; 0.029]	-0.96	-0.024	[-0.095; 0.046]	-0.68	0.067	[-0.004; 0.137]	1.86
M	-0.056*	[-0.109; -0.004]	-2.10	-0.058*	[-0.105; -0.012]	-2.45	-0.023	[-0.065; 0.019]	-1.08	0.061**	[0.016; 0.105]	2.67	0.008	[-0.039; 0.056]	0.35
O	0.229***	[0.176; 0.282]	8.52	0.154***	[0.105; 0.203]	6.17	0.099***	[0.061; 0.138]	5.07	-0.186***	[-0.230; -0.142]	-8.23	0.186***	[0.145; 0.227]	8.83
N	-0.166***	[-0.213; -0.119]	-6.86	-0.016	[-0.060; 0.028]	-0.71	-0.083***	[-0.120; -0.045]	-4.31	0.261***	[0.217; 0.306]	11.55	-0.065**	[-0.113; -0.018]	-2.69
De	0.019	[-0.084; 0.121]	0.36	-0.009	[-0.105; 0.086]	-0.19	-0.092*	[-0.166; -0.017]	-2.20	-0.010	[-0.105; 0.086]	-0.20	-0.014	[-0.099; 0.072]	-0.32
S	0.099***	[0.061; 0.137]	5.10	0.033	[-0.003; 0.070]	1.80	0.042**	[0.015; 0.069]	3.01	-0.059***	[-0.093; -0.026]	-3.53	0.080***	[0.047; 0.113]	4.73

Note. Model selection was based on the lowest AIC value. Coefficients refer to Model IV (with random slope and intercept). D = Duty, I = Intellect, A = Adversity, M = Mating, O = Positivity, N = Negativity, De = Deception, S = Sociality.Confidence intervals are two-tailed 95% CIs. *b* = estimate (unstandardized) effect; *t* = t-value. *p < 0.05 **, p < 0.01, ***p < 0.001.

Table S5

Unstandardized Fixed L1 Effects of Within-Person Centered Situation Perceptions on Big Five States in the ***Naturalistic Design Controlling for All Situation Perceptions***

	Extraversion			Conscientiousness			Agreeableness			Neuroticism			Openness		
	b	[CI]	*t*	*b*	[CI]	*t*	*b*	[CI]	*t*	*b*	[CI]	*t*	*b*	[CI]	*t*
D	0.03***	[0.03; 0.04]	18.73	0.03***	[0.03; 0.03]	15.95	0.01***	[0.01; 0.01]	6.49	< 0.001	[-<0.001; 0.01]	1.28	0.01***	[0.01; 0.01]	5.93
I	0.02***	[0.02; 0.02]	9.68	0.02***	[0.02; 0.03]	10.5	0.01***	[0.01; 0.01]	5.74	<-0.001*	[-0.01; <0.001]	-1.97	0.04***	[0.03; 0.04]	17.83
A	0.02***	[0.01; 0.03]	5.82	0.01*	[<0.001; 0.01]	2.29	-0.03***	[-0.04; -0.03]	-9.45	< 0.001	[<-0.001; 0.01]	1.01	0.1*	[<0.001; 0.01]	2.13
M	-0.01*	[-0.01; <-0.001]	-2.44	-0.01***	[-0.02; -0.01]	-5.17	0.01***	[0.01; 0.02]	5.75	-0.01***	[-0.01; -0.01]	-4.05	< 0.001	[<-0.001; 0.01]	0.74
O	0.09***	[0.08; 0.09]	30.47	0.07***	[0.07; 0.08]	24.55	0.09***	[0.08; 0.09]	32.78	-0.12***	[-0.13; -0.11]	38.14	0.10***	[0.09; 0.10]	34.99
N	-0.06***	[-0.06; -0.05]	-21.15	-0.06***	[-0.07; -0.06]	-22.37	-0.06***	[-0.07; -0.05]	-22.94	0.13***	[0.12; 0.14]	42.1	-0.06***	[-0.06; -0.05]	-22.03
De	-0.01**	[-0.02; -0.01]	-3.09	-0.02***	[-0.03; -0.01]	-3.88	-0.03***	[-0.04; -0.02]	-6.9	0.02***	[0.02; 0.03]	5.13	-0.01***	[-0.02; <-0.001]	-2.98
S	0.04***	[0.04; 0.05]	26.57	0.03***	[0.03; 0.03]	17.06	0.02***	[0.02; 0.03]	16.16	-0.01***	[-0.02; -0.01]	-7.96	0.01***	[<0.001; 0.01]	3.92
T	< 0.001	[<-0.001; <0.001]	-0.05	0.01***	[0.01; 0.02]	4.37	<-0.001	[-0.01; <0.001]	-0.71	< 0.001	[<-0.001; 0.01]	0.55	-0.01*	[-0.01; <-0.001]	-2.33

Note. Model selection was based on the lowest AIC value. Coefficients refer to Model IV (with random slope and intercept). D = Duty, I = Intellect, A = Adversity, M = Mating, O = Positivity, N = Negativity, De = Deception, S = Sociality, T = Typicality. Confidence intervals are two-tailed 95% CIs. *b* = estimate (unstandardized) effect; *t* = t-value. *p < 0.05 **, p < 0.01, ***p < 0.001.

Table S6

Unstandardized Fixed Effects of Big Five Personality States on Situation Perceptions with Level 1 Interactions

Personality State	Situation Level	Duty			Intellect			Adversity			Mating			Positivity			Negativity			Deception			Sociality			Typicality		
Extraversion		*b*	CI	*t*	*b*	CI	*t*	*b*	CI	*t*	*b*	CI	*t*	*b*	CI	*t*	*b*	CI	*t*	*b*	CI	*t*	*b*	CI	*t*	*b*	CI	*t*
Social Partner (Reference group: Alone)	SP	0.03***	[0.027, 0.036]	13.95	0.03***	[0.027, 0.037]	11.73	-0.01	[-0.023, -0.001]	-2.19	0.01	[-0.001, 0.016]	1.80	0.10***	[0.097, 0.111]	29.09	-0.07***	[-0.078, -0.065]	-22.12	-0.04***	[-0.050, -0.023]	-5.31	0.05***	[0.045, 0.055]	19.20	0.03***	[0.028, 0.041]	10.82
	SP x Close Relations	-0.02***	[-0.029, -0.016]	-6.95	<0.001	[-0.012, 0.004]	-1.04	-0.03***	[-0.043, -0.014]	-3.79	0.01	[-0.004, 0.014]	1.02	0.02**	[0.006, 0.024]	3.34	-0.02***	[-0.028, -0.011]	-4.67	-0.02	[-0.042, -0.003]	-2.32	0.03***	[0.022, 0.037]	7.72	-0.03***	[-0.037, -0.021]	-7.09
	SP x Work/Study Colleagues	-0.03***	[-0.033, -0.017]	-5.90	-0.02***	[-0.030, -0.012]	-4.42	<0.001	[-0.019, 0.017]	-0.09	0.01	[-0.007, 0.018]	0.82	0.02*	[0.003, 0.027]	2.45	-0.01*	[-0.021, 0.000]	-1.96	<0.001	[-0.020, 0.024]	0.19	0.02***	[0.014, 0.035]	4.61	-0.02***	[-0.034, -0.013]	-4.34
	SP x Other People	-0.03***	[-0.036, -0.022]	-7.66	-0.01*	[-0.020, -0.001]	-2.25	-0.01	[-0.028, 0.009]	-0.98	0.02*	[0.004, 0.027]	2.58	0.02***	[0.013, 0.033]	4.55	-0.02**	[-0.026, -0.007]	-3.30	-0.02	[-0.037, 0.009]	-1.18	0.03***	[0.018, 0.035]	6.30	-0.04***	[-0.049, -0.031]	-8.54
Location (Reference group: Private Spaces)	SP	0.02***	[0.014, 0.022]	8.50	0.03***	[0.029, 0.039]	13.53	-0.02***	[-0.026, -0.008]	-3.67	0.03***	[0.021, 0.030]	10.32	0.12***	[0.112, 0.124]	37.27	-0.08***	[-0.089, -0.077]	-28.02	-0.04***	[-0.055, -0.031]	-7.11	0.06***	[0.055, 0.063]	29.92	0.02***	[0.015, 0.027]	7.33
	SP x Public/Social Spaces	-0.02***	[-0.021, -0.010]	-5.84	-0.02***	[-0.030, -0.017]	-7.18	-0.01	[-0.018, 0.009]	-0.68	-0.01	[-0.012, 0.001]	-1.65	0.01	[-0.001, 0.014]	1.62	<0.001	[-0.011, 0.004]	-1.00	<0.001	[-0.017, 0.016]	-0.06	<0.001	[-0.006, 0.005]	-0.21	-0.01**	[-0.019, -0.005]	-3.39
	SP x Outdoor	-0.03*	[-0.049, -0.005]	-2.45	-0.05***	[-0.068, -0.021]	-3.71	-0.01	[-0.058, 0.040]	-0.36	<0.001	[-0.016, 0.019]	0.18	0.01	[-0.022, 0.034]	0.42	-0.01	[-0.038, 0.019]	-0.65	0.01	[-0.057, 0.079]	0.31	<0.001	[-0.012, 0.021]	0.50	-0.05***	[-0.071, -0.027]	-4.35

	SP x Other	0.01	[-0.006, 0.019]	1.05	-0.03***	[-0.043, -0.012]	-3.50	<0.001	[-0.026, 0.032]	0.19	0.01	[-0.005, 0.021]	1.21	<0.001	[-0.017, 0.014]	-0.22	<0.001	[-0.019, 0.012]	-0.42	0.01	[-0.029, 0.052]	0.57	<0.001	[-0.008, 0.017]	0.70	<0.001	[-0.013, 0.014]	0.03
	SP	0.01**	[0.003, 0.013]	3.35	0.03***	[0.028, 0.040]	11.11	-0.02*	[-0.027, -0.005]	-2.89	0.03***	[0.025, 0.035]	11.48	0.14***	[0.130, 0.144]	40.14	-0.10***	[-0.101, -0.088]	-28.64	-0.04***	[-0.055, -0.028]	-5.98	0.07***	[0.067, 0.076]	33.69	0.01**	[0.004, 0.016]	3.11
	SP x Travel	-0.01	[-0.024, 0.011]	-0.68	-0.01	[-0.029, 0.015]	-0.63	-0.03	[-0.069, 0.006]	-1.66	-0.012**	[-0.021, 0.011]	-0.57	-0.02	[-0.035, 0.005]	-1.44	0.01	[-0.011, 0.028]	0.88	-0.02	[-0.071, 0.031]	-0.78	-0.02**	[-0.039, -0.008]	-2.96	0.02*	[0.003, 0.038]	2.31
Activity (Rerefence Group: Relaxation)	SP x Responsibilities /Work	0.01	[0.001, 0.014]	2.11	-0.02***	[-0.030, -0.016]	-6.17	0.01	[-0.002, 0.026]	1.62	-0.01**	[-0.019, -0.005]	-3.22	-0.01**	[-0.023, -0.006]	-3.21	0.01**	[0.004, 0.020]	3.08	<0.001	[-0.015, 0.021]	0.32	-0.02***	[-0.029, -0.018]	-8.53	0.01***	[0.006, 0.022]	3.52
	SP x Social/Interaction	-0.02***	[-0.026, -0.009]	-4.13	-0.01	[-0.018, 0.001]	-1.71	-0.03**	[-0.042, -0.010]	-3.21	-0.01	[-0.017, -0.001]	-2.24	<0.001	[-0.008, 0.012]	0.32	-0.01**	[-0.024, -0.005]	-2.93	-0.02	[-0.042, 0.000]	-1.95	0.01*	[0.001, 0.016]	2.18	-0.01*	[-0.022, -0.004]	-2.69
	SP x Other	0.02**	[0.007, 0.029]	3.25	-0.02*	[-0.031, -0.004]	-2.53	-0.02	[-0.050, 0.001]	-1.85	-0.01	[-0.016, 0.007]	-0.83	-0.03***	[-0.044, -0.017]	-4.41	0.02**	[0.010, 0.036]	3.47	0.02	[-0.011, 0.058]	1.34	-0.02**	[-0.026, -0.007]	-3.33	-0.01	[-0.017, 0.007]	-0.84
Conscientiousness																												
	SP	0.02***	[0.01, 0.02]	7.27	0.02***	[0.02, 0.03]	7.88	-0.03***	[-0.05, -0.02]	-5.99	<0.001	[-0.01, 0.00]	-1.01	0.12***	[0.12, 0.13]	32.84	-0.09***	[-0.10, -0.09]	-26.96	-0.05***	[-0.07, -0.04]	-7.31	0.04***	[0.04, 0.05]	15.38	0.04***	[0.04, 0.05]	13.18
Social Partner (Reference group: Alone)	SP x Close Relations	-0.01***	[-0.02, -0.01]	-4.25	<0.001	[-0.01, 0.01]	-0.35	-0.02*	[-0.04, -0.01]	-2.78	0.01*	[0.01, 0.02]	2.93	-0.02***	[-0.03, -0.01]	-5.05	0.01	[-0.00, 0.01]	1.55	-0.02	[-0.04, -0.00]	-2.16	0.01	[0.00, 0.02]	2.08	-0.03***	[-0.04, -0.02]	-7.37
	SP x Work/Study Colleagues	<0.001	[-0.01, 0.01	0.90	-0.01	[-0.02, 0.00	-1.56	0.01	[-0.01,	1.48	0.01	[-0.00, 0.02	1.32	-0.02**	[-0.03, -	-3.13	0.01	[-0.00, 0.02	1.42	<0.001	[-0.02,	0.39	0.01	[0.00, 0.02	2.19	-0.02***	[-0.03, -	-3.74

			]			]			0.03]			]			0.01]			]			0.03]			]			0.01]	
	SP x Other People	-0.01***	[-0.02, -0.01]	-3.60	-0.01*	[-0.02, -0.00]	-2.77	<0.001	[-0.02, 0.02]	-0.24	0.01	[0.00, 0.03]	2.07	-0.04***	[-0.05, -0.03]	-7.32	0.02**	[0.01, 0.03]	3.06	-0.02	[-0.04, 0.01]	-1.39	-0.01*	[-0.02, -0.00]	-2.45	-0.03***	[-0.04, -0.02]	-6.76
	SP	0.01	[0.00, 0.01]	2.23	0.02***	[0.02, 0.03]	7.99	-0.04***	[-0.05, -0.03]	-8.40	0.02***	[0.01, 0.02]	6.40	0.12***	[0.11, 0.13]	36.22	-0.09***	[-0.10, -0.09]	-30.19	-0.06***	[-0.07, -0.05]	-9.67	0.04***	[0.03, 0.04]	19.27	0.03***	[0.03, 0.04]	10.61
Location (Reference group: Private Spaces)	SP x Public/Social Spaces	0.01***	[0.01, 0.02]	3.97	<0.001	[-0.01, 0.00]	-1.24	0.01	[-0.01, 0.02]	0.86	-0.01***	[-0.02, -0.01]	-3.80	-0.03***	[-0.04, -0.02]	-6.90	0.01**	[0.01, 0.02]	3.20	0.01	[-0.01, 0.02]	0.68	<0.001	[-0.01, 0.00]	-1.51	<0.001	[-0.01, 0.01]	-0.22
	SP x Outdoor	-0.02	[-0.04, 0.01]	-1.45	-0.03*	[-0.06, -0.01]	-2.58	0.01	[-0.04, 0.06]	0.22	<0.001	[-0.02, 0.02]	-0.20	-0.03	[-0.06, 0.00]	-1.78	0.02	[-0.01, 0.05]	1.23	<0.001	[-0.07, 0.07]	-0.10	-0.02	[-0.03, 0.00]	-1.95	-0.04***	[-0.07, -0.02]	-3.70
	SP x Other	0.01	[0.00, 0.03]	2.22	-0.01	[-0.02, 0.01]	-0.94	0.03	[-0.00, 0.06]	1.69	<0.001	[-0.02, 0.01]	-0.58	-0.03***	[-0.05, -0.01]	-3.53	0.02*	[0.01, 0.04]	2.69	<0.001	[-0.04, 0.04]	0.16	-0.01	[-0.02, 0.01]	-0.79	-0.01	[-0.03, 0.00]	-1.58
	SP	-0.01	[-0.01, -0.00]	-2.27	0.02***	[0.02, 0.03]	6.93	-0.04***	[-0.05, -0.03]	-7.26	0.02***	[0.01, 0.02]	7.18	0.13***	[0.12, 0.13]	35.44	-0.1***	[-0.11, -0.09]	-28.88	-0.06***	[-0.07, -0.04]	-8.30	0.05***	[0.04, 0.05]	22.19	0.02***	[0.02, 0.03]	7.03
Activity (Rerefence Group: Relaxation)	SP x Travel	<0.001	[-0.02, 0.01]	-0.36	-0.01	[-0.03, 0.01]	-0.65	-0.01	[-0.05, 0.03]	-0.36	<0.001	[-0.02, 0.01]	-0.36	-0.02*	[-0.04, -0.00]	-2.27	<0.001	[-0.02, 0.02]	0.01	-0.06	[-0.11, -0.00]	-2.06	-0.02*	[-0.04, -0.01]	-2.64	0.01	[-0.01, 0.03]	1.10
	SP x Responsibilities/Work	0.02***	[0.01, 0.03]	6.24	-0.01**	[-0.02, -0.01]	-3.35	0.02*	[0.01, 0.04]	2.57	-0.01***	[-0.02, -0.01]	-3.62	<0.001	[-0.01, 0.01]	0.90	<0.001	[-0.01, 0.01]	0.85	0.01	[-0.01, 0.03]	0.96	<0.001	[-0.01, 0.00]	-0.80	0.01***	[0.01, 0.02]	3.59
	SP x Social/Interaction	<0.001	[-0.01, 0.01	-0.69	<0.001	[-0.01, 0.01	-0.54	<0.001	[-0.02,	-0.44	-0.01	[-0.01, 0.00	-1.83	-0.02**	[-0.03, -	-3.37	0.01	[0.00, 0.02	2.06	-0.01	[-0.03,	-0.64	0.01	[-0.00, 0.01	1.37	-0.01*	[-0.02, -	-2.83

			]			]			0.01]			]			0.01]			]			0.01]			]			0.00]	
	SP x Other	0.04***	[0.03, 0.05]	6.26	-0.01	[-0.02, 0.01]	-0.90	<0.001	[-0.03, 0.03]	0.08	-0.01	[-0.02, 0.00]	-1.23	-0.03***	[-0.04, -0.02]	-4.11	0.03***	[0.01, 0.04]	3.73	0.04	[0.00, 0.07]	1.95	-0.01	[-0.02, -0.00]	-2.12	-0.01	[-0.03, 0.00]	-1.94
Agreeableness																												
Social Partner (Reference group: Alone)	SP	-0.01**	[-0.01, -0.00]	-2.74	-0.01*	[-0.01, -0.00]	-2.61	-0.1***	[-0.11, -0.09]	-17.00	0.01*	[0.00, 0.02]	2.05	0.13***	[0.12, 0.14]	37.05	-0.1***	[-0.10, -0.09]	-30.50	-0.09***	[-0.10, -0.07]	-12.71	0.02***	[0.02, 0.03]	9.88	0.04***	[0.03, 0.04]	12.98
	SP x Close Relations	-0.02***	[-0.03, -0.02]	-8.05	-0.01	[-0.01, 0.00]	-1.75	-0.03***	[-0.04, -0.02]	-4.15	0.02***	[0.01, 0.03]	5.17	0.02***	[0.01, 0.03]	5.51	-0.03***	[-0.04, -0.02]	-7.89	-0.03**	[-0.05, -0.01]	-3.47	0.04***	[0.03, 0.04]	9.93	-0.03***	[-0.04, -0.02]	-8.03
	SP x Work/Study Colleagues	-0.01**	[-0.02, -0.00]	-2.90	<0.001	[-0.01, 0.01]	0.91	0.01	[-0.01, 0.03]	1.30	0.02*	[0.00, 0.03]	2.52	0.02**	[0.01, 0.03]	2.90	-0.01	[-0.02, 0.00]	-1.52	-0.01	[-0.03, 0.01]	-0.76	0.03***	[0.02, 0.04]	5.37	-0.03***	[-0.04, -0.03]	-6.79
	SP x Other People	-0.02***	[-0.03, -0.01]	-5.02	0.01	[0.00, 0.02]	2.20	0.02	[-0.00, 0.03]	1.81	0.02***	[0.01, 0.03]	3.79	<0.001	[-0.01, 0.01]	0.36	-0.01**	[-0.02, -0.01]	-2.97	-0.02	[-0.04, 0.00]	-1.85	0.03***	[0.03, 0.04]	8.23	-0.05***	[-0.06, -0.04]	-10.64
Location (Reference group: Private Spaces)	SP	-0.02***	[-0.02, -0.02]	-9.95	-0.01**	[-0.01, -0.00]	-3.27	-0.1***	[-0.11, -0.09]	-20.41	0.04***	[0.03, 0.04]	15.61	0.14***	[0.14, 0.15]	46.28	-0.11***	[-0.12, -0.11]	-37.57	-0.1***	[-0.11, -0.09]	-15.78	0.05***	[0.04, 0.05]	24.79	0.03***	[0.02, 0.03]	9.28
	SP x Public/Social Spaces	<0.001	[-0.01, 0.00]	-1.15	<0.001	[-0.01, 0.00]	-0.65	<0.001	[-0.01, 0.02]	0.62	<0.001	[-0.01, 0.00]	-0.99	<0.001	[-0.01, 0.01]	-0.51	<0.001	[-0.01, 0.01]	-0.37	<0.001	[-0.02, 0.01]	-0.15	<0.001	[-0.01, 0.00]	-1.00	-0.03***	[-0.03, -0.02]	-7.44
	SP x Outdoor	-0.01	[-0.03, 0.01]	-1.06	-0.02	[-0.04, 0.00]	-1.67	-0.05	[-0.10, -0.00]	-2.15	<0.001	[-0.02, 0.01]	-0.23	0.02	[-0.01, 0.04]	1.42	-0.04**	[-0.07, -0.01]	-3.07	-0.06	[-0.13, 0.00]	-1.95	<0.001	[-0.01, 0.02]	0.23	-0.04***	[-0.06, -0.02]	-3.54

	SP x Other	<0.001	[-0.01, 0.01]	0.46	<0.001	[-0.01, 0.02]	0.34	0.01	[-0.02, 0.03]	0.39	-0.01	[-0.02, 0.00]	-1.30	-0.03***	[-0.04, -0.01]	-4.00	0.01	[-0.00, 0.03]	1.88	<0.001	[-0.04, 0.04]	-0.05	0.01	[-0.01, 0.02]	1.13	-0.03***	[-0.04, -0.02]	-4.70
	SP	-0.02***	[-0.02, -0.01]	-7.89	<0.001	[-0.00, 0.01]	0.78	-0.1***	[-0.11, -0.09]	-17.18	0.04***	[0.04, 0.05]	15.69	0.15***	[0.14, 0.15]	43.87	-0.11***	[-0.12, -0.11]	-34.02	-0.1***	[-0.11, -0.08]	-14.04	0.05***	[0.05, 0.06]	26.36	0.01***	[0.01, 0.02]	4.05
	SP x Travel	-0.01	[-0.03, 0.01]	-0.93	0.01	[-0.01, 0.03]	0.49	<0.001	[-0.04, 0.03]	-0.17	-0.01	[-0.02, 0.01]	-1.21	-0.01	[-0.02, 0.01]	-0.56	-0.02	[-0.04, 0.00]	-2.00	-0.01	[-0.06, 0.04]	-0.43	-0.03***	[-0.04, -0.01]	-3.61	0.01	[-0.01, 0.02]	0.73
Activity (Rerefence Group: Relaxation)	SP x Responsibilities /Work	0.01*	[0.00, 0.01]	2.34	-0.01	[-0.01, 0.00]	-1.93	0.01	[-0.00, 0.03]	1.72	-0.01**	[-0.02, -0.00]	-3.04	<0.001	[-0.01, 0.01]	0.08	<0.001	[-0.01, 0.01]	0.08	<0.001	[-0.02, 0.02]	0.10	-0.02***	[-0.02, -0.01]	-6.50	0.01***	[0.01, 0.02]	3.81
	SP x Social/Interaction	-0.02***	[-0.02, -0.01]	-3.70	-0.01	[-0.02, -0.00]	-2.27	-0.02	[-0.03, 0.00]	-2.02	-0.01**	[-0.02, -0.00]	-3.09	<0.001	[-0.01, 0.01]	0.52	-0.02***	[-0.03, -0.01]	-3.79	-0.02	[-0.04, 0.00]	-1.56	<0.001	[-0.00, 0.01]	0.78	<0.001	[-0.01, 0.01]	-0.02
	SP x Other	0.01*	[0.00, 0.02]	2.61	-0.01	[-0.03, 0.00]	-1.87	<0.001	[-0.02, 0.03]	0.23	<0.001	[-0.01, 0.01]	-0.38	-0.03***	[-0.04, -0.02]	-4.77	0.02***	[0.01, 0.04]	4.03	0.03	[-0.00, 0.07]	1.90	-0.01	[-0.01, 0.00]	-1.17	-0.01	[-0.02, 0.01]	-1.18
Neuroticism																														
	SP	0.04***	[0.04, 0.05]	15.57	0.04***	[0.04, 0.05]	13.10	0.12***	[0.10, 0.13]	17.58	<0.001	[-0.01, 0.01]	-0.96	-0.21***	[-0.21, -0.20]	-50.08	0.19***	[0.18, 0.20]	50.99	0.12***	[0.10, 0.13]	14.60	-0.01*	[-0.01, -0.00]	-2.13	-0.05***	[-0.06, -0.04]	-13.69
Social Partner (Reference group: Alone)	SP x Close Relations	0.02***	[0.02, 0.03]	6.34	<0.001	[-0.01, 0.01]	0.55	0.03***	[0.02, 0.05]	4.01	-0.03***	[-0.04, -0.01]	-4.70	-0.01*	[-0.02, -0.00]	-2.72	0.03***	[0.02, 0.04]	6.11	0.04***	[0.02, 0.07]	3.80	-0.06***	[-0.07, -0.05]	-12.67	0.03***	[0.02, 0.04]	7.12
	SP x Work/Study Colleagues	0.01	[-0.00, 0.02	1.28	-0.02***	[-0.03, -	-4.00	-0.02*	[-0.04, -	-2.29	-0.01	[-0.02, 0.01	-1.27	0.01*	[0.00, 0.03	1.99	-0.02*	[-0.03, -	-2.75	-0.02	[-0.04,	-1.40	-0.04***	[-0.06, -	-7.02	0.05***	[0.03, 0.06	7.29

			]			0.01]			0.00]			]			]			0.01]			0.01]			0.03]			]	
	SP x Other People	0.01	[0.00, 0.02]	2.14	-0.02***	[-0.03, -0.01]	-4.27	-0.01	[-0.03, 0.02]	-0.54	-0.03***	[-0.04, -0.01]	-3.84	0.02*	[0.00, 0.03]	2.62	<0.001	[-0.01, 0.01]	0.71	0.03	[0.00, 0.06]	2.25	-0.05***	[-0.06, -0.04]	-9.73	0.05***	[0.04, 0.07]	9.96
	SP	0.05***	[0.05, 0.06]	21.55	0.04***	[0.03, 0.04]	13.26	0.12***	[0.11, 0.13]	21.32	-0.04***	[-0.04, -0.03]	-12.09	-0.21***	[-0.22, -0.21]	-57.92	0.2***	[0.20, 0.21]	58.25	0.13***	[0.12, 0.15]	18.85	-0.04***	[-0.05, -0.04]	-19.33	-0.04***	[-0.05, -0.04]	-12.48
Location (Reference group: Private Spaces)	SP x Public/Social Spaces	<0.001	[-0.01, 0.00]	-0.55	<0.001	[-0.01, 0.01]	-0.51	<0.001	[-0.02, 0.01]	-0.09	-0.01*	[-0.02, -0.00]	-2.74	0.01*	[0.00, 0.02]	2.53	-0.01*	[-0.02, -0.00]	-2.59	-0.02	[-0.04, 0.00]	-1.94	<0.001	[-0.01, 0.01]	0.04	0.05***	[0.04, 0.06]	11.98
	SP x Outdoor	<0.001	[-0.02, 0.03]	0.22	-0.01	[-0.04, 0.02]	-0.77	0.05	[-0.01, 0.10]	1.66	<0.001	[-0.02, 0.03]	0.47	-0.02	[-0.05, 0.01]	-1.16	0.04*	[0.01, 0.07]	2.39	0.07	[-0.01, 0.15]	1.67	-0.01	[-0.03, 0.01]	-1.07	0.09***	[0.06, 0.11]	6.56
	SP x Other	0.01	[-0.01, 0.02]	0.98	0.01	[-0.01, 0.02]	0.62	<0.001	[-0.04, 0.03]	-0.12	-0.01	[-0.02, 0.01]	-1.01	0.02*	[0.01, 0.04]	2.63	0.01	[-0.01, 0.03]	1.28	-0.01	[-0.06, 0.03]	-0.56	0.01	[0.00, 0.03]	1.99	0.03***	[0.02, 0.05]	4.01
	SP	0.05***	[0.05, 0.06]	18.66	0.03***	[0.02, 0.03]	7.07	0.12***	[0.11, 0.13]	18.14	-0.04***	[-0.05, -0.04]	-13.61	-0.22***	[-0.23, -0.21]	-54.51	0.21***	[0.20, 0.21]	54.11	0.14***	[0.12, 0.15]	17.06	-0.05***	[-0.06, -0.05]	-21.59	-0.02***	[-0.02, -0.01]	-4.54
Activity (Rerefence Group: Relaxation)	SP x Travel	0.01	[-0.01, 0.03]	1.14	0.01	[-0.01, 0.04]	0.93	0.02	[-0.02, 0.06]	0.92	<0.001	[-0.02, 0.01]	-0.47	0.01	[-0.02, 0.03]	0.60	<0.001	[-0.02, 0.03]	0.29	0.01	[-0.05, 0.07]	0.24	0.02*	[0.00, 0.04]	2.39	-0.01	[-0.03, 0.01]	-0.82
	SP x Responsibilities/Work	-0.01***	[-0.02, -0.01]	-3.58	<0.001	[-0.01, 0.01]	0.68	-0.02	[-0.04, -0.00]	-2.44	0.01*	[0.00, 0.02]	2.75	0.01	[-0.00, 0.02]	1.45	-0.02***	[-0.03, -0.01]	-4.31	-0.03*	[-0.05, -0.01]	-2.61	0.02***	[0.01, 0.03]	5.92	-0.02***	[-0.03, -0.01]	-4.97
	SP x Social/Interaction	0.01	[0.00, 0.02	2.20	0.01	[0.00, 0.02	2.01	0.01	[-0.01,	0.69	0.02***	[0.01, 0.03	3.75	0.01	[0.00, 0.02	2.13	<0.001	[-0.01, 0.01	-0.92	<0.001	[-0.03,	-0.32	-0.01	[-0.01, 0.00	-1.26	-0.01	[-0.02, 0.01	-0.91

			]			]			0.03]			]			]			]			0.02]			]			]	
	SP x Other	-0.01	[-0.03, -0.00]	-2.08	0.01	[-0.01, 0.02]	1.06	<0.001	[-0.03, 0.03]	0.25	<0.001	[-0.01, 0.02]	0.52	0.03***	[0.02, 0.05]	4.30	-0.03**	[-0.04, -0.01]	-3.44	-0.03	[-0.07, 0.01]	-1.63	0.03***	[0.01, 0.04]	4.38	-0.02*	[-0.03, -0.00]	-2.57
Openness																												
Social Partner (Reference group: Alone)	SP	0.01*	[0.00, 0.01]	2.32	0.02***	[0.02, 0.03]	9.01	-0.04***	[-0.05, -0.03]	-7.16	0.01	[0.00, 0.02]	1.91	0.13***	[0.12, 0.13]	36.86	-0.09***	[-0.09, -0.08]	-27.96	-0.05***	[-0.06, -0.04]	-7.87	0.02***	[0.01, 0.03]	8.23	0.03***	[0.02, 0.04]	9.51
	SP x Close Relations	-0.01***	[-0.02, -0.01]	-4.28	<0.001	[-0.00, 0.01]	0.90	-0.03***	[-0.04, -0.01]	-3.93	0.01	[-0.00, 0.02]	1.69	<0.001	[-0.01, 0.01]	-0.53	-0.01*	[-0.02, -0.00]	-2.80	-0.02	[-0.03, 0.00]	-1.62	0.02***	[0.02, 0.03]	6.78	-0.02***	[-0.03, -0.01]	-5.41
	SP x Work/Study Colleagues	-0.02***	[-0.03, -0.01]	-4.55	<0.001	[-0.01, 0.01]	-0.14	0.01	[-0.01, 0.03]	1.00	0.01	[-0.00, 0.02]	1.68	0.02**	[0.01, 0.03]	3.31	<0.001	[-0.01, 0.01]	-0.75	<0.001	[-0.02, 0.03]	0.41	0.03***	[0.01, 0.04]	4.97	-0.03***	[-0.04, -0.02]	-5.72
	SP x Other People	-0.02***	[-0.02, -0.01]	-4.32	<0.001	[-0.01, 0.01]	0.62	-0.01	[-0.02, 0.01]	-0.63	0.01*	[0.00, 0.03]	2.55	-0.01	[-0.01, 0.00]	-1.25	<0.001	[-0.01, 0.01]	-0.31	-0.01	[-0.03, 0.01]	-0.74	0.03***	[0.02, 0.04]	6.81	-0.04***	[-0.04, -0.03]	-8.15
Location (Reference group: Private Spaces)	SP	<0.001	[-0.01, 0.00]	-0.66	0.03***	[0.02, 0.03]	11.46	-0.05***	[-0.06, -0.04]	-10.91	0.02***	[0.01, 0.02]	7.31	0.13***	[0.12, 0.13]	41.09	-0.09***	[-0.10, -0.09]	-32.70	-0.06***	[-0.07, -0.05]	-10.45	0.03***	[0.03, 0.03]	16.31	0.02***	[0.02, 0.03]	8.10
	SP x Public/Social Spaces	-0.01***	[-0.02, -0.01]	-5.61	-0.02***	[-0.02, -0.01]	-5.68	0.01	[-0.01, 0.02]	1.06	0.01**	[0.00, 0.02]	2.93	0.01*	[0.00, 0.02]	2.71	<0.001	[-0.01, 0.01]	0.26	0.01	[-0.00, 0.03]	1.82	<0.001	[-0.00, 0.01]	0.89	-0.03***	[-0.03, -0.02]	-8.03
	SP x Outdoor	-0.01	[-0.03, 0.01]	-0.74	-0.01	[-0.04, 0.01]	-1.13	0.02	[-0.03, 0.06]	0.76	0.01	[-0.01, 0.03]	0.89	-0.01	[-0.03, 0.02]	-0.55	<0.001	[-0.02, 0.03]	0.23	0.02	[-0.05, 0.08]	0.46	<0.001	[-0.01, 0.02]	0.08	-0.03**	[-0.05, -0.01]	-3.15
	SP x Other	<0.00	[-	0.4	-0.01	[-	-	0.01	[-	0.9	0.01	[-	1.1	-0.01	[-	-	0.02	[0.0	1.9	<0.00	[-	-	0.01	[-	0.9	-	[-	-

		1	0.01, 0.01]	4		0.02, 0.01]	1.00		0.01, 0.04]	7		0.01, 0.02]	7		0.03, 0.00]	1.98		0, 0.03]	9	1	0.04, 0.04]	0.05		0.01, 0.02]	3	0.02*	0.03, -0.00]	2.56
	SP	-0.01*	[-0.01, -0.00]	-2.78	0.03***	[0.03, 0.04]	11.89	-0.04***	[-0.05, -0.03]	-8.11	0.02***	[0.02, 0.03]	8.99	0.13***	[0.13, 0.14]	40.50	-0.1***	[-0.10, -0.09]	-30.44	-0.05***	[-0.07, -0.04]	-8.40	0.04***	[0.03, 0.04]	18.56	0.01***	[0.01, 0.02]	4.03
	SP x Travel	-0.02	[-0.03, 0.00]	-1.82	-0.01	[-0.03, 0.01]	-0.61	-0.01	[-0.05, 0.02]	-0.64	-0.01	[-0.03, 0.00]	-1.42	-0.01	[-0.03, 0.01]	-1.10	0.01	[-0.01, 0.03]	0.71	-0.02	[-0.07, 0.03]	-0.68	-0.01	[-0.03, 0.00]	-1.78	0.01	[-0.01, 0.02]	0.79
Activity (Rerefence Group: Relaxation)	SP x Responsibilities /Work	<0.001	[-0.01, 0.01]	-0.07	-0.02***	[-0.02, -0.01]	-4.33	0.01	[-0.01, 0.02]	1.03	<0.001	[-0.01, 0.00]	-1.41	0.01	[0.00, 0.02]	2.13	<0.001	[-0.00, 0.01]	1.08	<0.001	[-0.02, 0.02]	0.11	-0.02***	[-0.03, -0.01]	-7.37	<0.001	[-0.00, 0.01]	0.94
	SP x Social/Interaction	<0.001	[-0.01, 0.00]	-1.22	-0.02**	[-0.03, -0.01]	-3.42	-0.02*	[-0.04, -0.00]	-2.54	<0.001	[-0.01, 0.00]	-1.02	-0.01	[-0.02, -0.00]	-2.27	-0.01	[-0.01, 0.00]	-1.13	-0.01	[-0.03, 0.01]	-0.78	0.01	[-0.00, 0.01]	1.76	<0.001	[-0.01, 0.01]	-0.89
	SP x Other	0.02*	[0.01, 0.03]	3.03	<0.001	[-0.01, 0.01]	-0.29	-0.01	[-0.03, 0.02]	-0.61	-0.01	[-0.02, 0.00]	-1.30	-0.01	[-0.02, 0.00]	-1.61	0.01	[-0.00, 0.02]	1.87	0.01	[-0.02, 0.04]	0.59	-0.01	[-0.02, 0.00]	-1.48	-0.01	[-0.02, 0.00]	-1.90

Note. Coefficients are based on Model IV, which includes random slopes and intercepts, and incorporates Level 1 interaction terms with the situation variables: Social Partner, Location, and Activity. For Social Partner, the reference category is Alone, with comparisons to Close Relations, Work/Study Colleagues, and Other People. For Location, the reference category is Private Spaces (e.g., home, friend's place), with comparisons to Public/Social Spaces (e.g., restaurants, events), Outdoor Spaces (e.g., nature), and Other. For Activity, the reference category is Relaxation/Leisure/Self-Care, with comparisons to Travel, Responsibilities/Work, Social/Interaction, and Other. *p*-values for interaction terms were adjusted using the Bonferroni-Holm method within each regression model — that is, across the $k - 1$ dummy-coded interaction terms of the respective situational variable (3 for Social Partner, 3 for Location, 4 for Activity). SP = Situation Perception; *b* = estimate (unstandardized) effect; *t* = t-value. Confidence intervals are two-tailed 95% CIs. *p < 0.05 **, p < 0.01, ***p < 0.001.

Table S7

Proportion of Variance in Random Effects Explained by Adding Situational Level-1 Interactions (Compared to Model IV)

Situation Perception	Situation Variable	Extraversion		Conscientiousness		Agreeableness		Neuroticism		Openness	
		Slope	Intercept	Slope	Intercept	Slope	Intercept	Slope	Intercept	Slope	Intercept
Duty	People Present	0.0870	0.0266	0.0709	0.0207	0.0680	0.0256	0.0572	0.0205	0.0257	0.0081
	Location	0.0521	0.0183	0.0772	0.0209	0.0271	0.0078	0.0335	0.0047	0.0185	0.0049
	Activity	0.0389	0.0067	0.0459	0.0085	0.0120	0.0006	0.0056	0.0018	0.0098	0.0006
Intellect	People Present	0.0857	0.0272	0.0562	0.0190	0.0823	0.0247	0.0686	0.0183	0.0291	0.0097
	Location	0.0611	0.0182	0.0701	0.0184	0.0208	0.0069	0.0093	0.0037	0.0141	0.0040
	Activity	0.0143	0.0028	0.0234	0.0020	0.0069	-0.0016	0.0008	0.0022	0.0186	-0.0006
Adversity	People Present	0.0977	0.0284	0.0759	0.0191	0.0595	0.0278	0.0646	0.0199	0.0222	0.0088
	Location	0.0585	0.0222	0.0600	0.0203	0.0068	0.0078	0.0126	0.0027	0.0100	0.0057
	Activity	0.0426	0.0073	0.0267	0.0026	0.0049	-0.0007	0.0019	<0.0001	0.0097	<0.0001
Mating	People Present	0.0495	0.0249	0.0744	0.0185	0.0228	0.0140	0.0368	0.0102	0.0150	0.0044
	Location	0.0798	0.0229	0.1114	0.0204	0.0100	0.0078	0.0169	0.0027	0.0289	0.0059
	Activity	0.0388	0.0093	0.0435	0.0044	0.0052	-0.0004	0.0233	0.0010	0.0032	0.0003
Positivty	People Present	0.0676	0.0166	0.0642	0.0064	0.0188	0.0107	0.0157	<0.0001	0.0102	0.0005
	Location	0.0628	0.0166	0.0759	0.0125	0.0061	0.0037	0.0038	<0.0001	0.0077	0.0023
	Activity	0.0816	0.0174	0.0819	0.0079	0.0091	0.0015	0.0028	0.0007	0.0185	0.0041
Negativity	People Present	0.0574	0.0233	0.0562	0.0127	0.0307	0.0190	0.0328	0.0127	0.0133	0.0042
	Location	0.0459	0.0208	0.0655	0.0183	0.0087	0.0065	0.0101	0.0049	0.0050	0.0049
	Activity	0.0581	0.0158	0.0395	0.0076	0.0022	0.0016	0.0018	0.0012	0.0100	0.0028
Deception	People Present	0.0721	0.0272	0.0616	0.0180	0.0352	0.0234	0.0533	0.0151	0.0222	0.0078

	Location	0.0218	0.0224	0.0524	0.0205	0.0065	0.0076	0.0075	0.0028	0.0025	0.0058
	Activity	0.0071	0.0069	0.0086	0.0022	0.0021	-0.0012	0.0186	0.0003	0.0000	-0.0002
	People Present	0.0022	0.0021	0.0049	0.0014	0.0156	0.0037	0.0396	0.0056	0.0093	-0.0009
Sociality	Location	0.0061	0.0078	0.0160	0.0075	0.0015	0.0011	0.0136	0.0009	0.0010	0.0019
	Activity	0.0286	0.0057	0.0393	0.0017	0.0111	-0.0002	0.0367	0.0004	0.0056	0.0000
	People Present	0.1092	0.0277	0.0693	0.0181	0.1040	0.0245	0.1039	0.0172	0.0650	0.0078
Typicality	Location	0.0713	0.0213	0.0794	0.0201	0.0407	0.0079	0.0746	0.0039	0.0358	0.0058
	Activity	0.0209	0.0072	0.0230	0.0030	0.0129	0.0000	0.0295	0.0011	0.0037	<0.0001

Table S8

Standardized Fixed L1 Effects of Within-Person Centered Situation Perceptions on Big Five States in ***Laboratory*** *Data*

	Extraversion			Conscientiousness			Agreeableness			Neuroticism			Openness		
	β	[CI]	*t*	*β*	[CI]	*t*	*β*	[CI]	*t*	*β*	[CI]	*t*	*β*	[CI]	*t*
D	-0.12***	[-0.17; -0.07]	-4.70	-0.02	[-0.06; 0.03]	-0.67	-0.12***[a]	[-0.15; -0.09]	-8.23	0.19†††	[0.14; 0.23]	8.42	-0.12***	[-0.16; -0.07]	-5.30
I	0.06*	[0.01; 0.11]	2.57	0.07***	[0.04; 0.11]	3.83	-0.01	[-0.05; 0.02]	-0.61	0.04	[-0.00; 0.08]	1.76	0.04*	[0.00; 0.09]	2.14
A	-0.01[a]	[-0.05; 0.03]	-0.45	<0.001[a]	[-0.03; 0.03]	0.01	-0.08***	[-0.12; -0.05]	-4.53	0.08[a] ***	[0.04; 0.11]	4.42	-0.01[a]	[-0.04; 0.03]	-0.36
M	-0.03[a]	[-0.07; 0.00]	-1.93	-0.02[a]	[-0.05; 0.01]	-1.33	-0.02[a]	[-0.04; 0.01]	-1.25	0.05*	[0.01; 0.10]	2.50	0.02[a]	[-0.01; 0.05]	1.34
O	0.27***	[0.23; 0.32]	11.81	0.14***	[0.10; 0.19]	6.46	0.16***	[0.12; 0.20]	8.34	-0.27***	[-0.32; -0.23]	-12.67	0.23***	[0.20; 0.27]	12.73
N	-0.21***	[-0.25; -0.17]	-10.24	-0.05*	[-0.09; -0.01]	-2.22	-0.15***	[-0.18; -0.11]	-7.66	0.32***	[0.28; 0.35]	16.35	-0.13***	[-0.17; -0.09]	-6.56
De	<-0.001[a]	[-0.04; 0.03]	-0.26	<0.001[a]	[-0.03; 0.03]	0.05	-0.07[a]***	[-0.10; -0.04]	-4.77	0.06*	[0.01; 0.11]	2.57	0.01 [a]	[-0.03; 0.04]	0.31
S	0.17***	[0.13; 0.22]	7.54	0.08†††	[0.04; 0.12]	3.95	0.08†††	[0.04; 0.11]	4.49	-0.12†††	[-0.17; -0.07]	-4.59	0.15***	[0.11; 0.19]	6.97

Note. Model selection was based on the lowest AIC value. Coefficients refer to Model IV, except for models with subscript [a], where Model III is reported as the AIC indicated better fit. Confidence intervals are two-tailed 95% CIs. *β* = estimate (standardized) effect; *t* = t-value. D = Duty, I = Intellect, A = Adversity, M = Mating, O = Positivity, N = Negativity, De = Deception, S = Sociality. *p < 0.05, **p < 0.01, ***p < 0.001. For the few associations with no hypotheses, we used Bonferroni-Holm adjusted the p-values to control the family-wise error rate.[†††]p holm-adj. < 0.001, for the associations with no hypotheses.

Table S9

Standardized Fixed L1 Effects of Within-Person Centered Situation Perceptions on Big Five States in the ***Naturalistic*** *Data*

	Extraversion			Conscientiousness			Agreeableness			Neuroticism			Openness		
	β	[CI]	*t*	*β*	[CI]	*t*	*β*	[CI]	*t*	*β*	[CI]	*t*	*β*	CI]	*t*
D	0.04***	[0.03; 0.05]	10.18	0.04***	[0.03; 0.05]	8.88	-0.04***	[-0.05; -0.03]	-10.94	0.10†††	[0.09; 0.10]	24.48	-0.01**	[-0.02; -0.00]	-3.08
I	0.06***	[0.05; 0.07]	14.19	0.05***	[0.04; 0.05]	11.92	-0.01**	[-0.02; -0.01]	-3.27	0.06***	[0.05; 0.07]	14.95	0.04***	[0.04; 0.05]	10.38
A	-0.01***	[-0.02; -0.01]	-3.63	-0.03***	[-0.03; -0.02]	-7.70	-0.09***	[-0.09; -0.08]	-22.96	0.09***	[0.08; 0.10]	24.96	-0.04***	[-0.05; -0.04]	-11.75
M	0.05***	[0.04; 0.05]	10.35	0.02***	[0.01; 0.02]	4.64	0.07***	[0.06; 0.08]	16.69	-0.07***	[-0.08; -0.06]	-14.91	0.04***	[0.03; 0.05]	9.66
O	0.18***	[0.17; 0.19]	39.89	0.14***	[0.14; 0.15]	34.13	0.21***	[0.20; 0.22]	49.45	-0.26***	[-0.27; -0.25]	-62.28	0.20***	[0.19; 0.21]	45.39
N	-0.13***	[-0.14; -0.12]	-29.93	-0.12***	[-0.13; -0.12]	-29.28	-0.18***	[-0.19; -0.17]	-40.60	0.27***	[0.26; 0.27]	62.83	-0.16***	[-0.17; -0.15]	-35.31
De	-0.03***	[-0.04; -0.02]	-7.28	-0.04***	[-0.04; -0.03]	-9.66	-0.07***	[-0.08; -0.07]	-18.04	0.08***	[0.07; 0.09]	21.06	-0.04***	[-0.05; -0.04]	-10.98
S	0.15***	[0.14; 0.16]	37.44	0.10†††	[0.09; 0.10]	26.96	0.10†††	[0.09; 0.11]	27.80	-0.08†††	[-0.08; -0.07]	-21.00	0.07***	[0.06; 0.08]	19.50
T	0.01†††	[0.01; 0.02]	4.93	0.03†††	[0.02; 0.03]	9.81	0.01†††	[0.01; 0.02]	4.7	-0.02†††	[-0.02; -0.01]	-5.85	0.01†††	[0.01; 0.01]	3.91

Note. Model selection was based on the lowest AIC value. Coefficients refer to Model IV, which had the best model fit. Confidence intervals are two-tailed 95% CIs. *β* = estimate (standardized) effect; *t* = t-value. D = Duty, I = Intellect, A = Adversity, M = Mating, O = Positivity, N = Negativity, De = Deception, S = Sociality, T = Typicality.*p < 0.05, **p < 0.01, ***p < 0.001. For the few associations with no hypotheses, we used Bonferroni-Holm adjusted the p-values to control the family-wise error rate.†††p holm-adj. < 0.001, for the associations with no hypotheses.

Figure S2. Extraversion and Adversity by Situation Variable

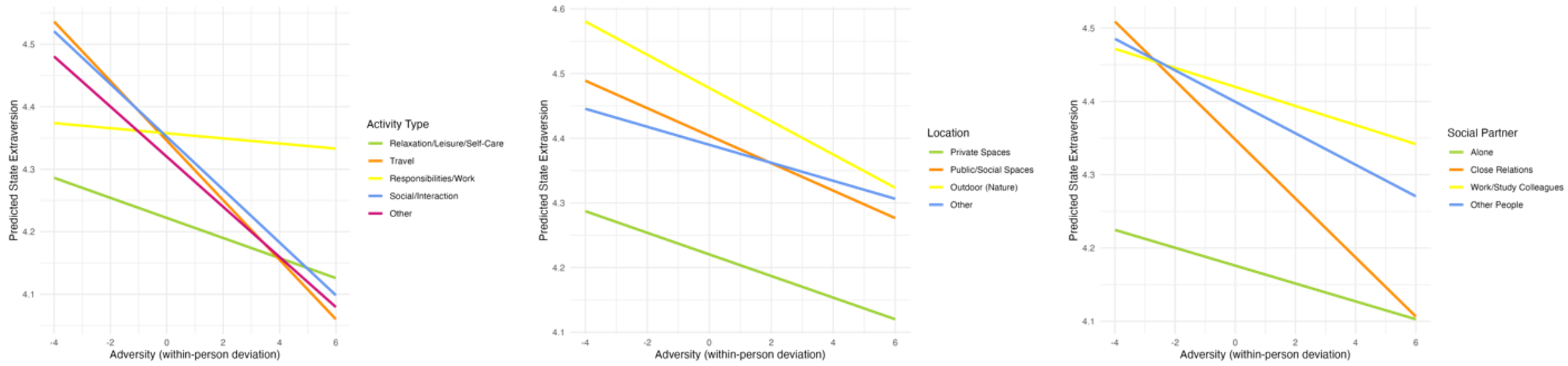

Figure S3. Extraversion and Deception by Situation Variable

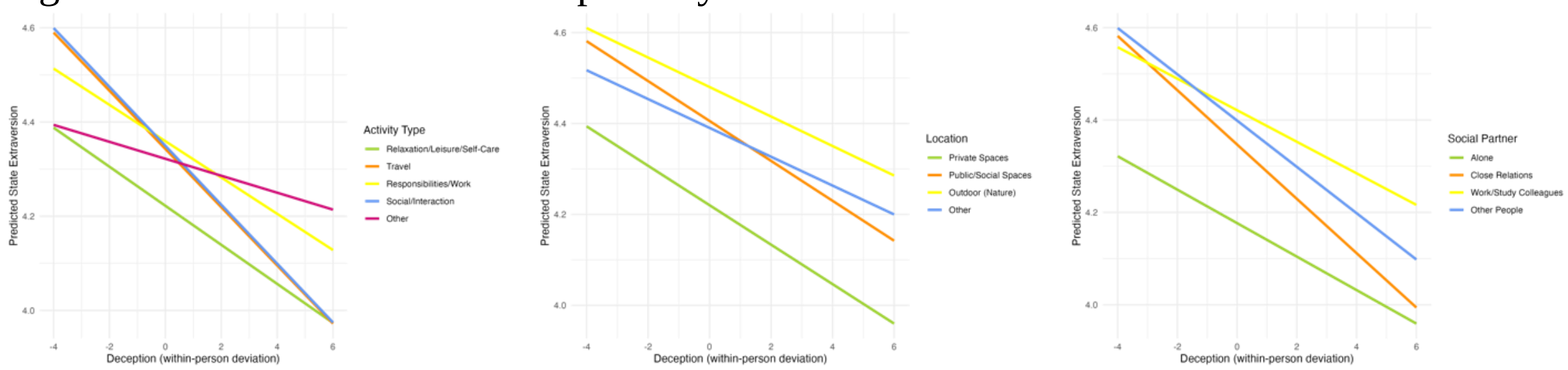

Figure S4. Extraversion and Duty by Situation Variable

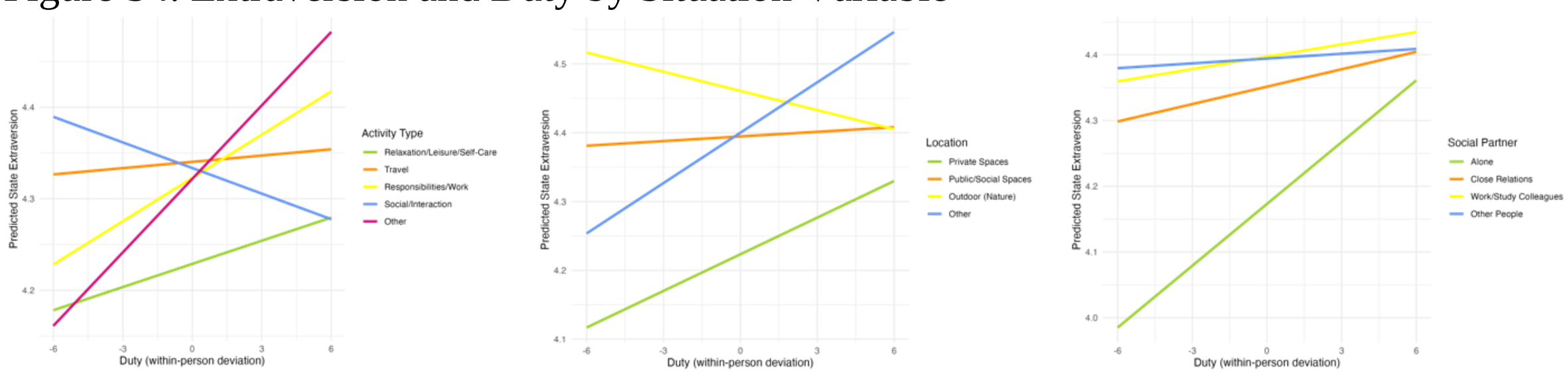

Figure S5. Extraversion and Intellect by Situation Variable

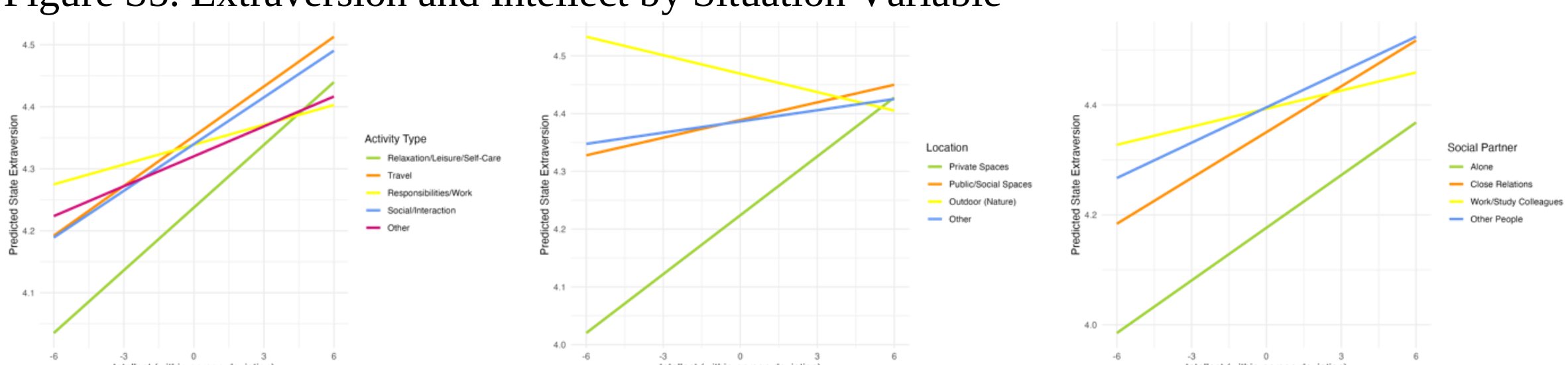

Figure S6. Extraversion and Mating by Situation Variable

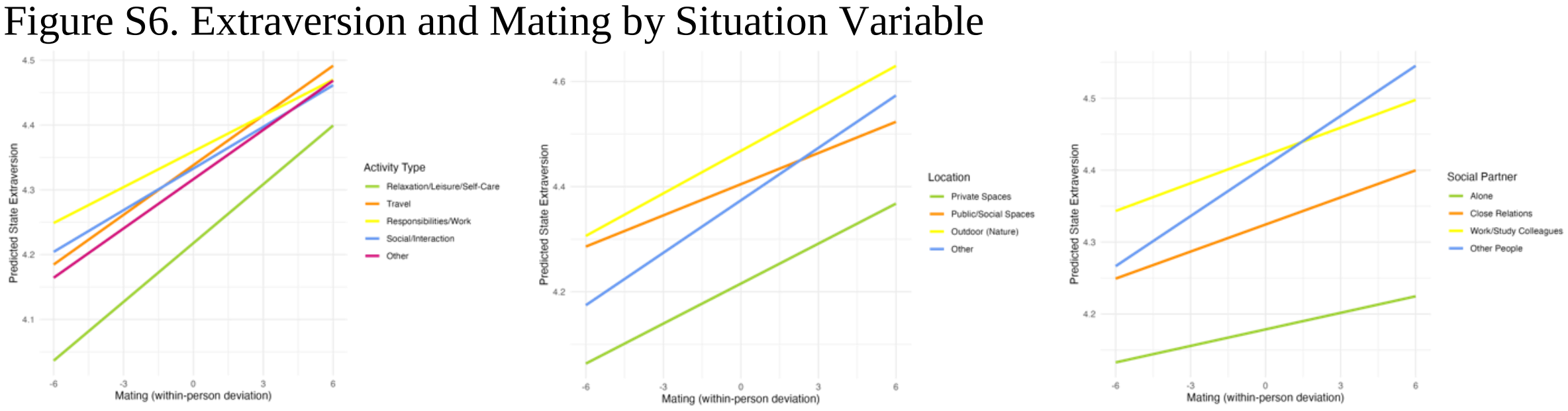

Figure S7. Extraversion and Negativity by Situation Variable

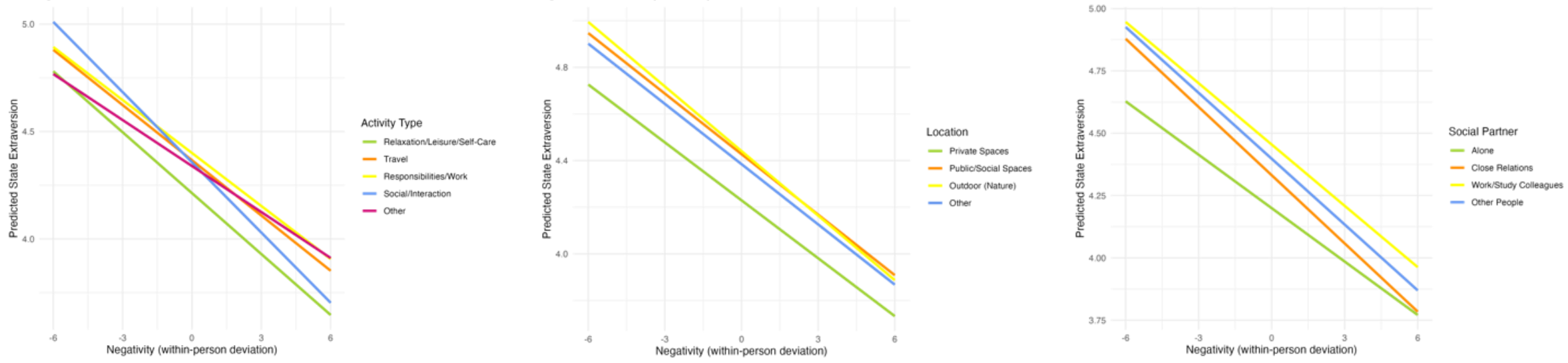

Figure S8. Extraversion and Positivity by Situation Variable

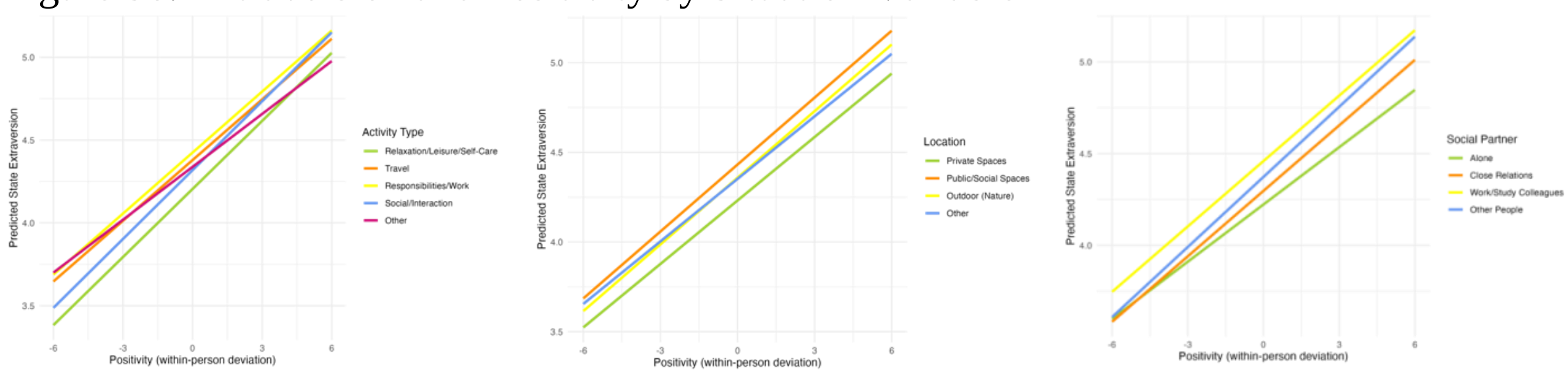

Figure S9. Extraversion and Sociality by Situation Variable

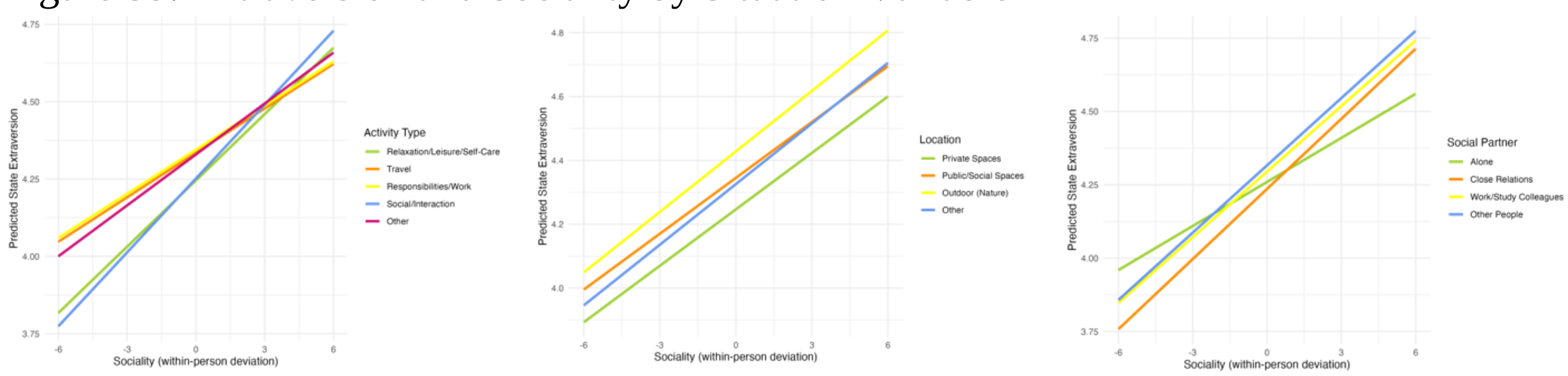

Figure S10. Extraversion and Typicality by Situation Variable

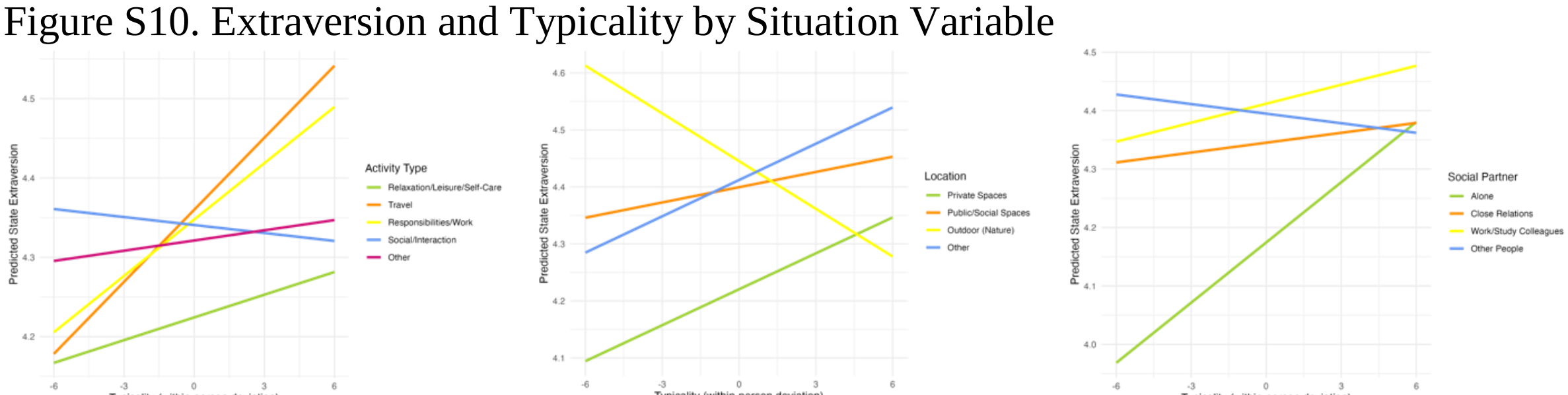

Figure S11. Conscientiousness and Adversity by Situation Variable

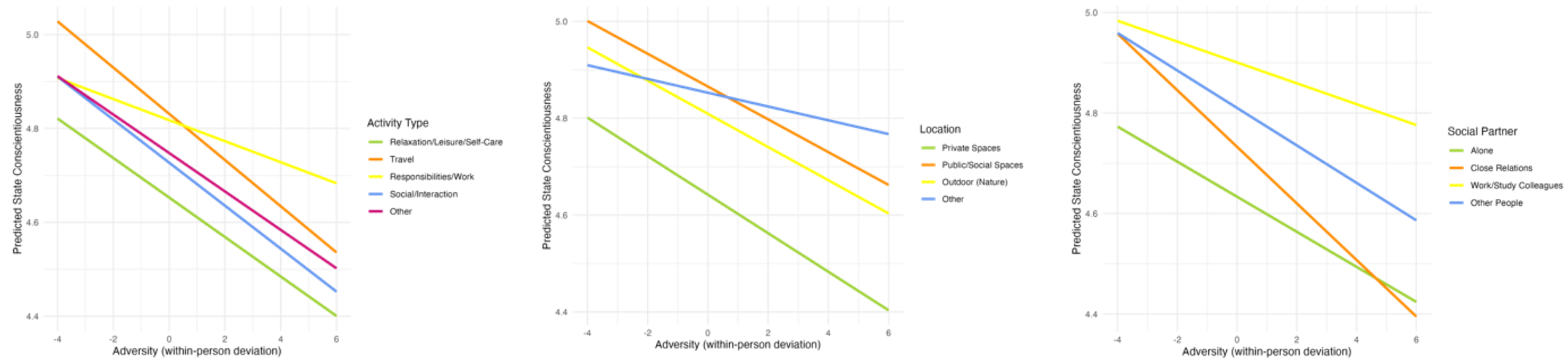

Figure S12. Conscientiousness and Deception by Situation Variable

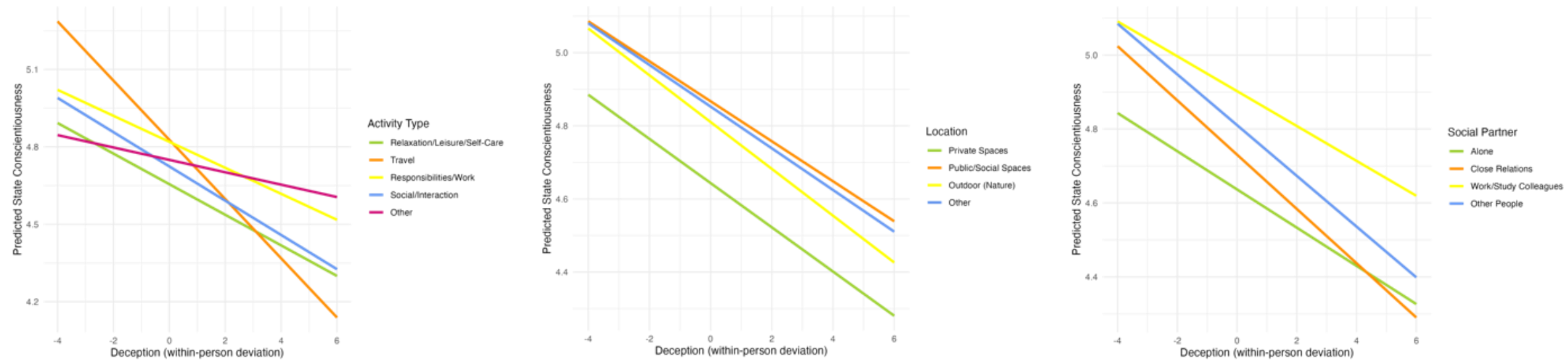

Figure S13. Conscientiousness and Duty by Situation Variable

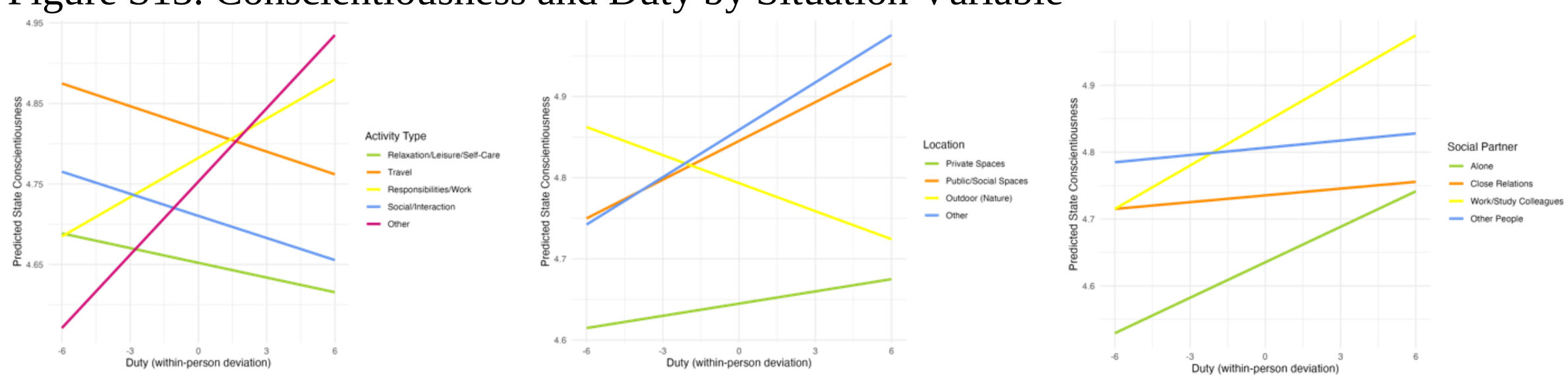

Figure S14. Conscientiousness and Intellect by Situation Variable

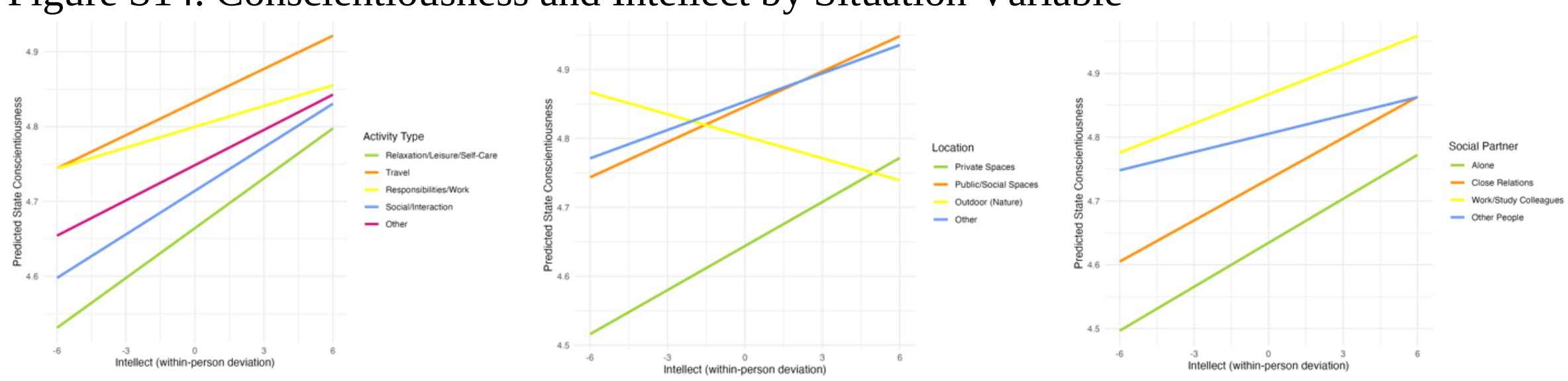

Figure S15. Conscientiousness and Mating by Situation Variable

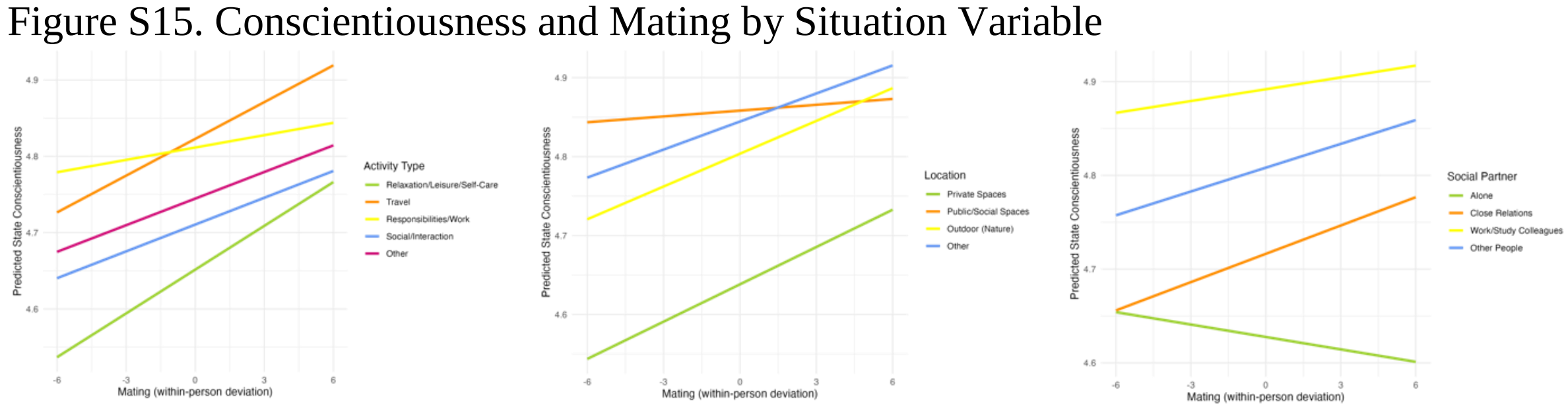

Figure S16. Conscientiousness and Negativity by Situation Variable

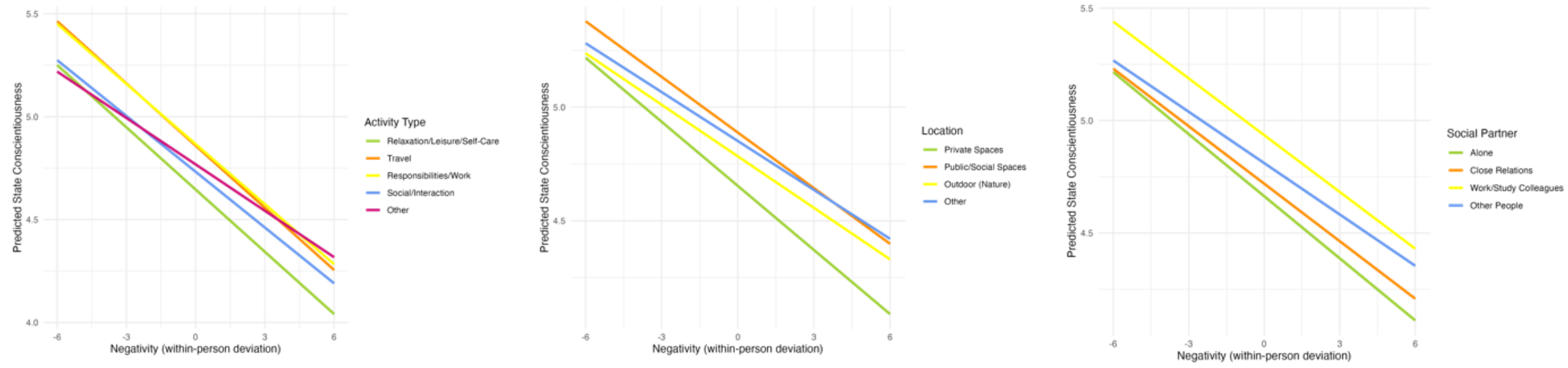

Figure S17. Conscientiousness and Positivity by Situation Variable

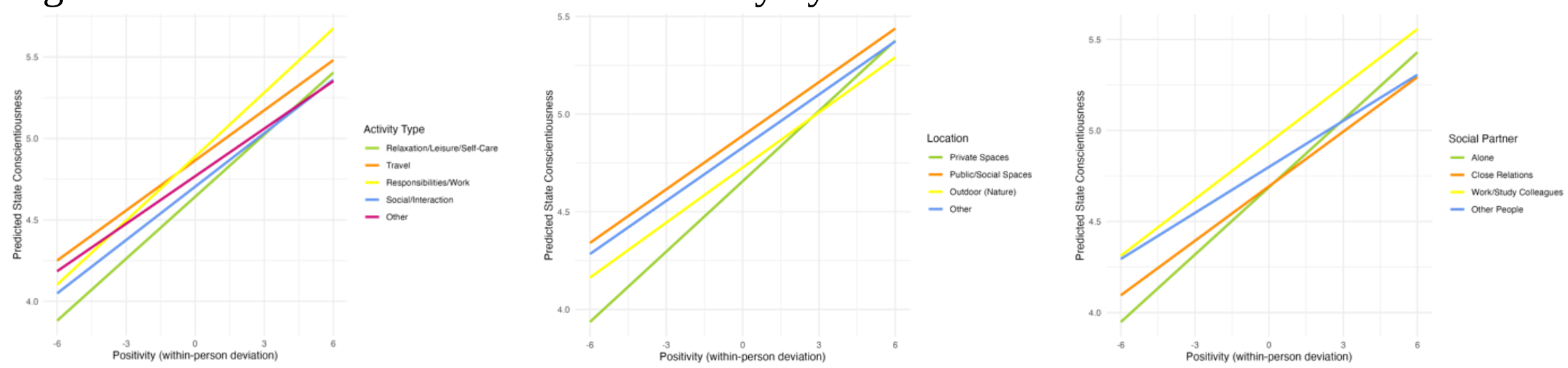

Figure S18. Conscientiousness and Sociality by Situation Variable

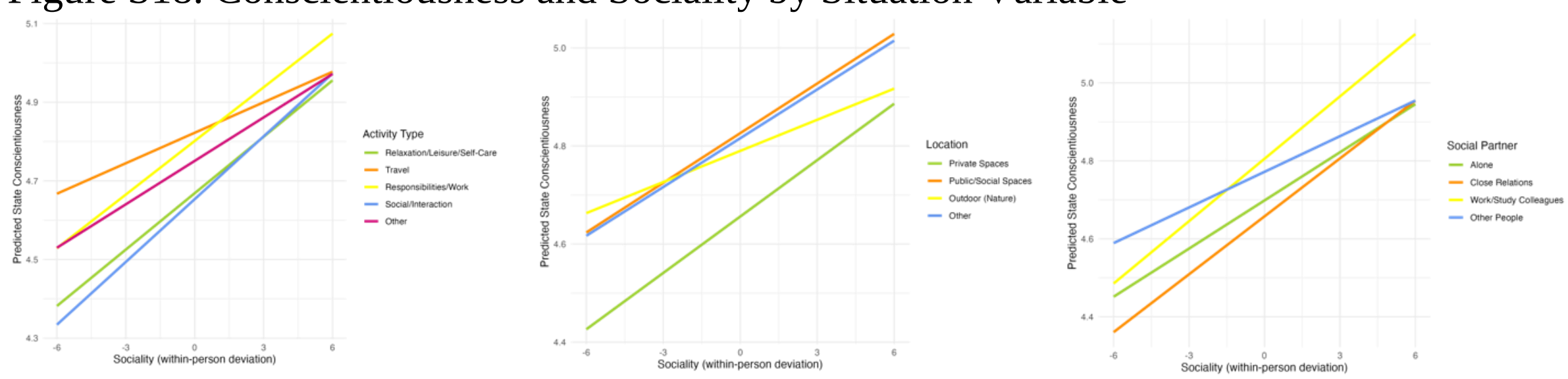

Figure S19. Conscientiousness and Typicality by Situation Variable

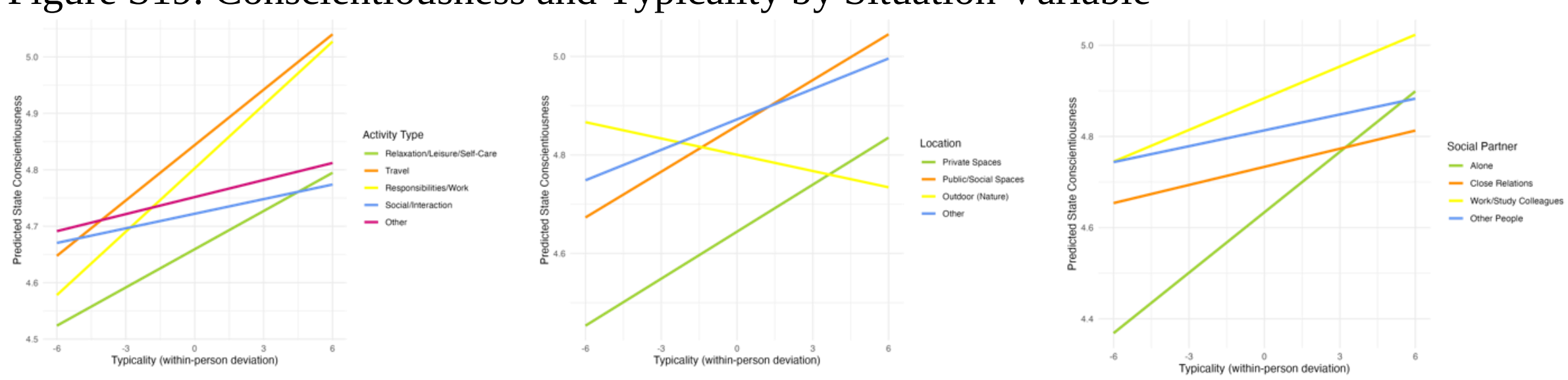

Figure S20. Agreeableness and Adversity by Situation Variable

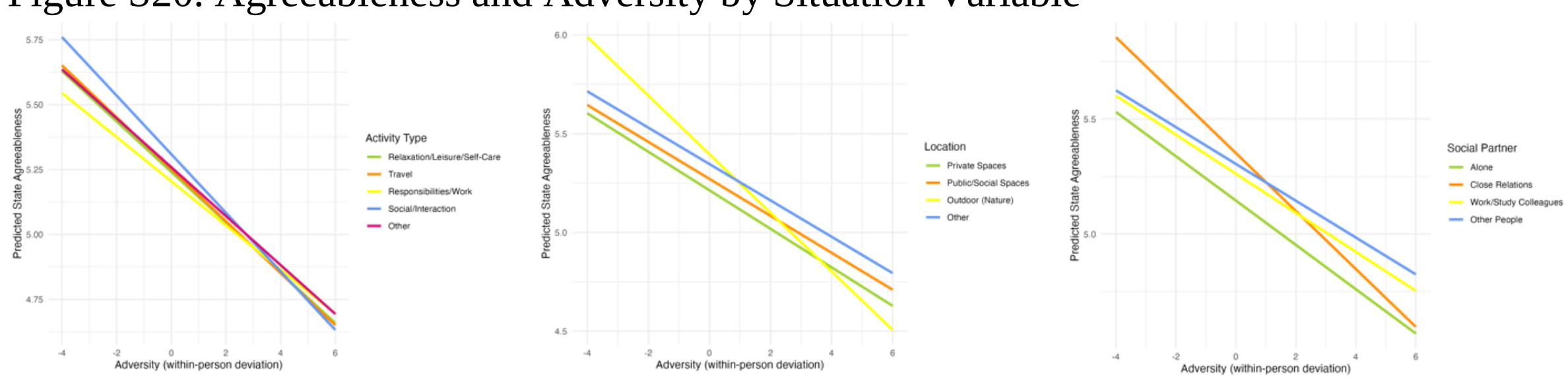

Figure S21. Agreeableness and Deception by Situation Variable

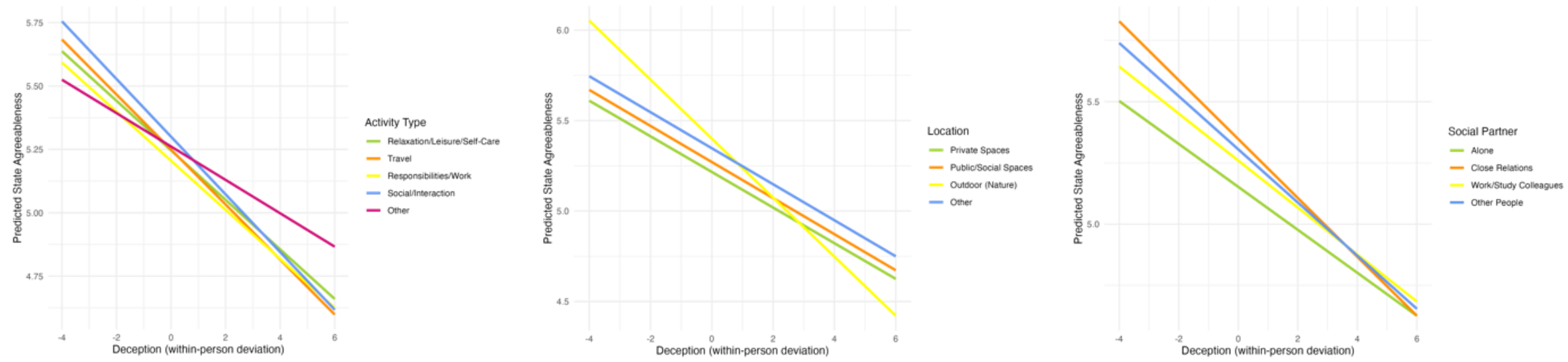

Figure S22. Agreeableness and Duty by Situation Variable

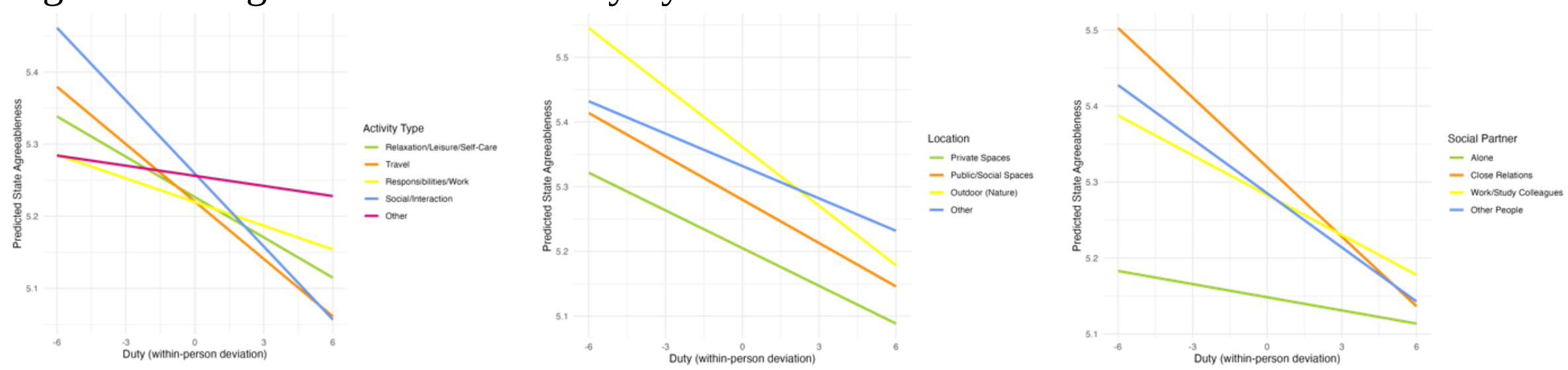

Figure S23. Agreeableness and Intellect by Situation Variable

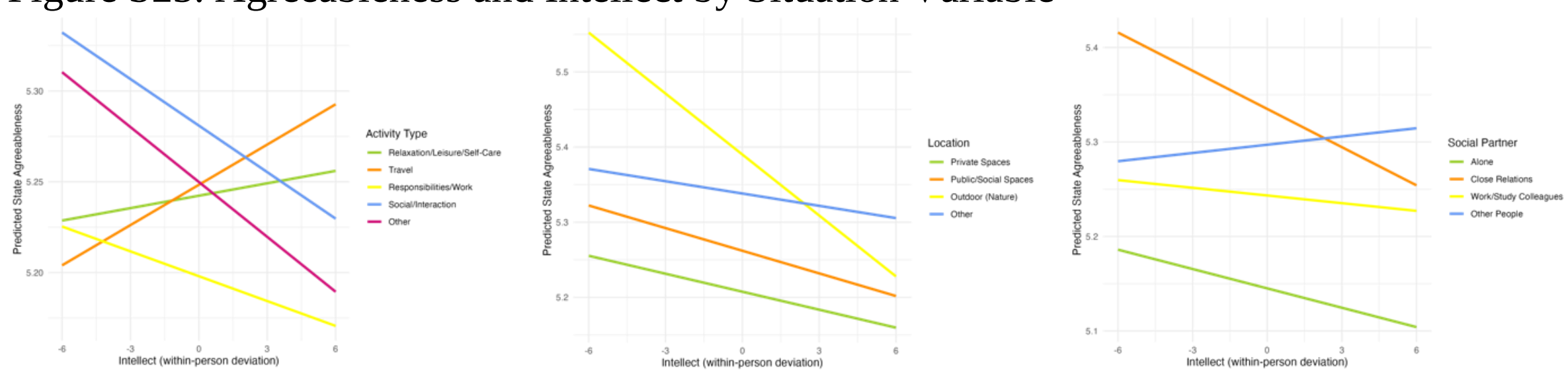

Figure S24. Agreeableness and Mating by Situation Variable

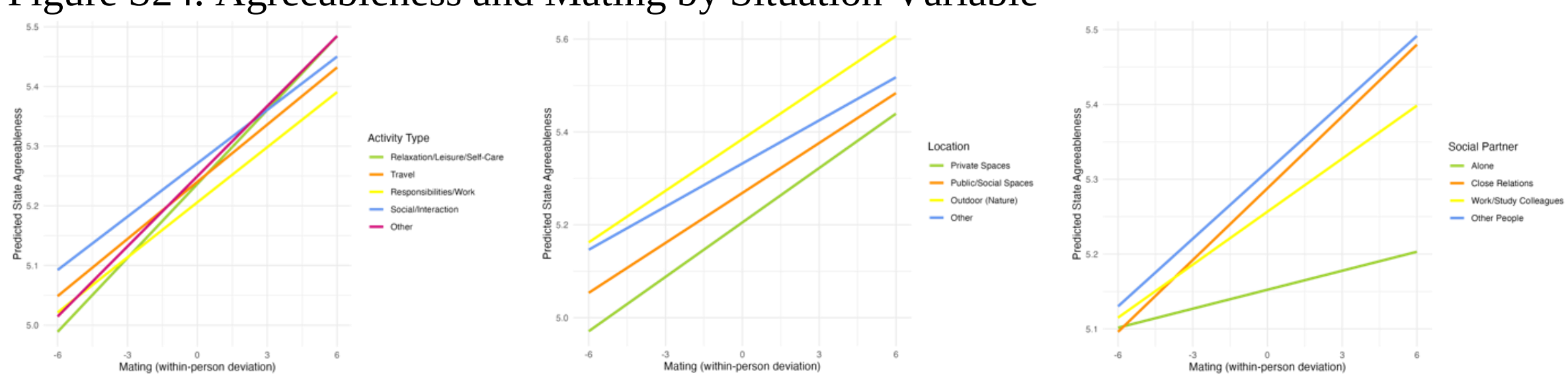

Figure S25. Agreeableness and Negativity by Situation Variable

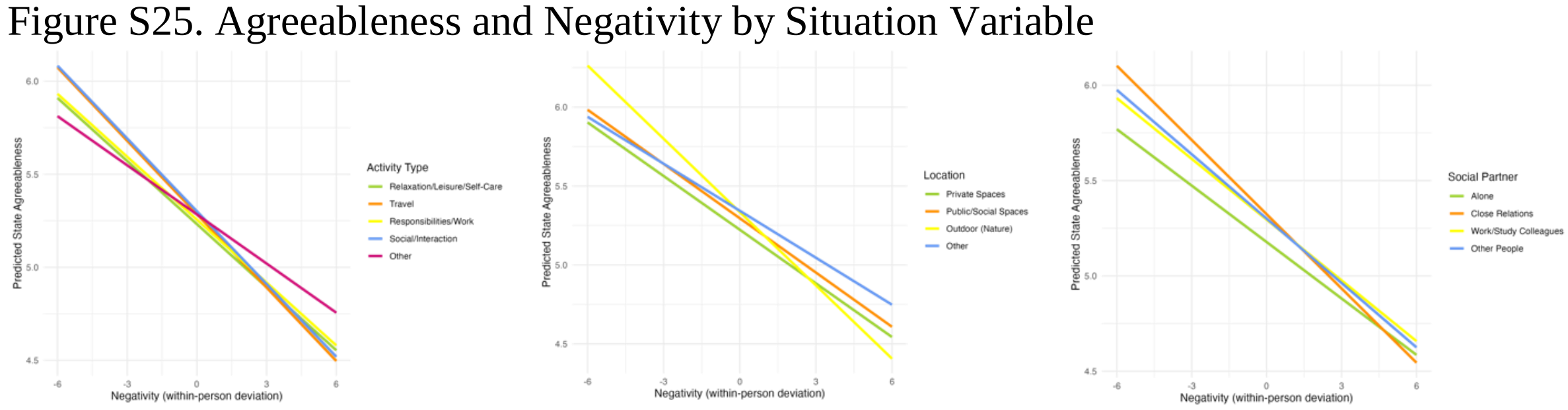

Figure S26. Agreeableness and Positivity by Situation Variable

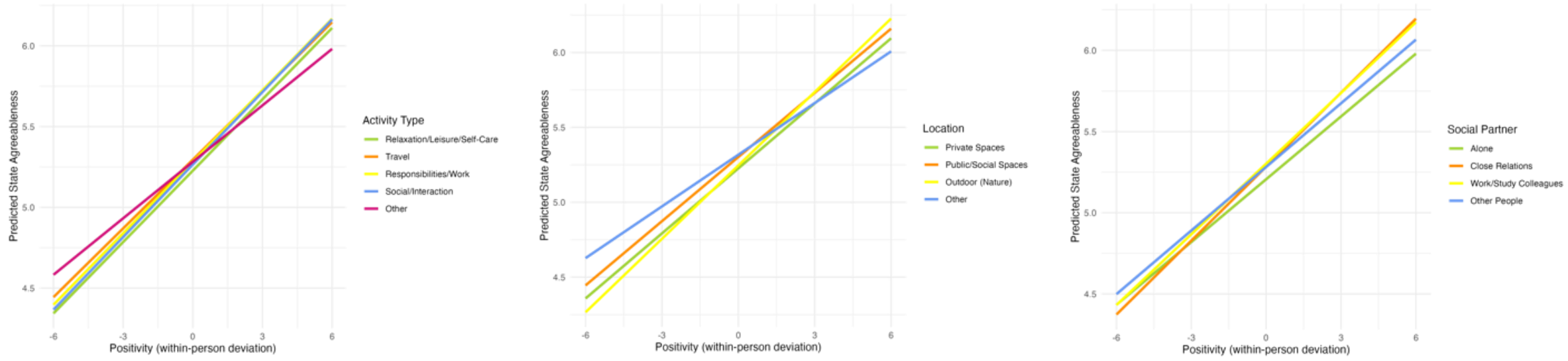

Figure S27. Agreeableness and Sociality by Situation Variable

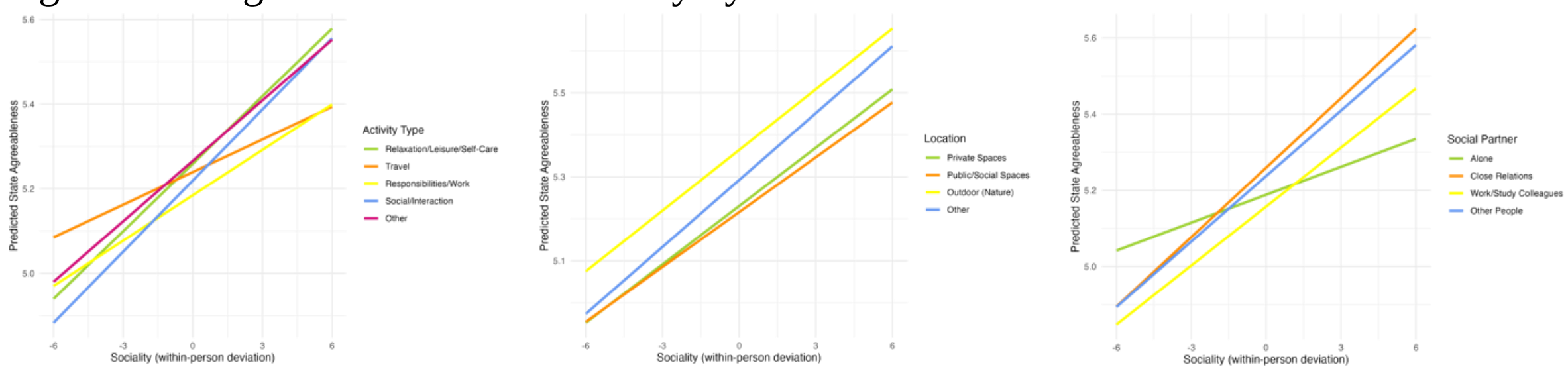

Figure S28. Agreeableness and Typicality by Situation Variable

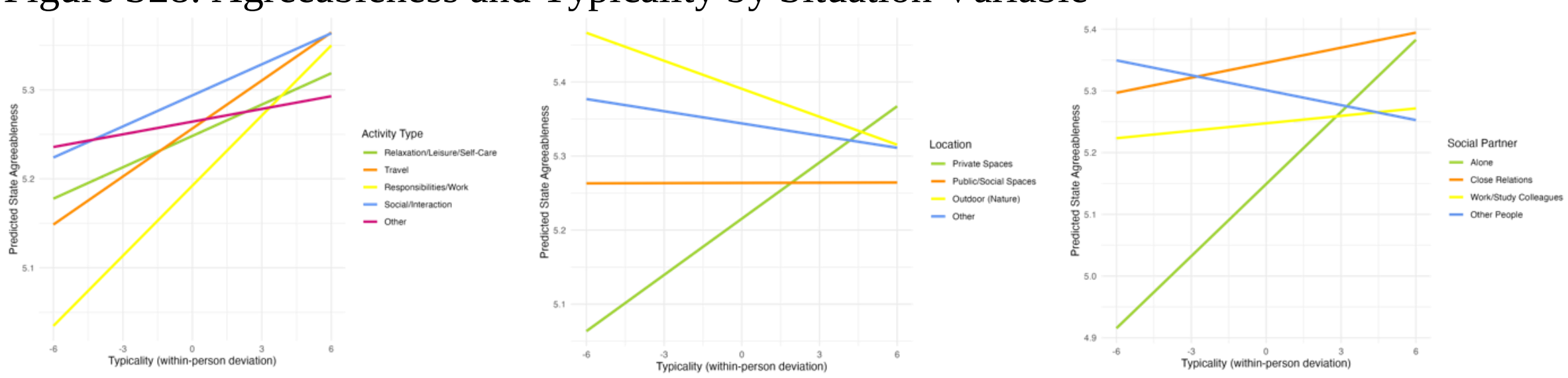

Figure S29. Neuroticism and Adversity by Situation Variable

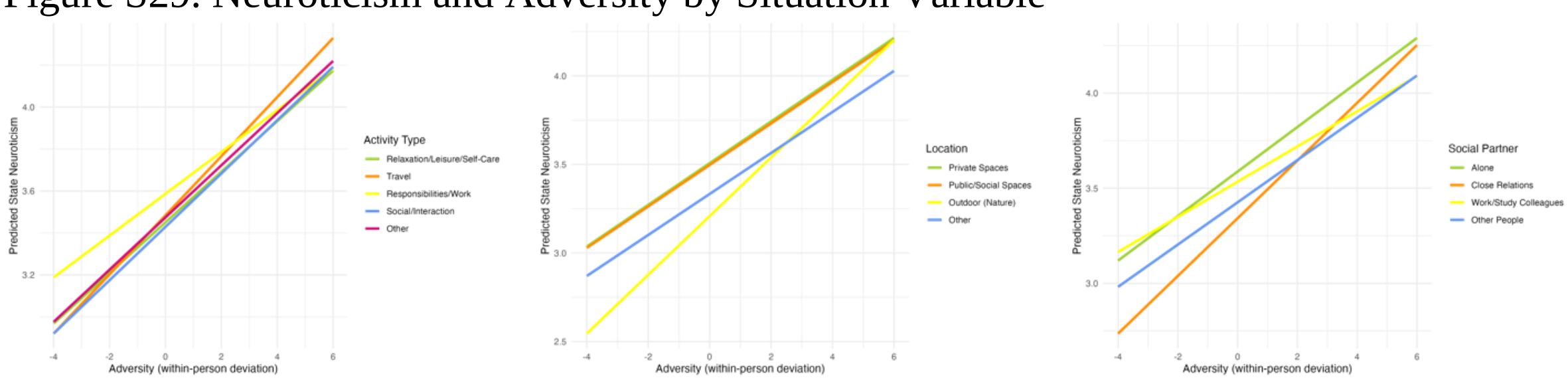

Figure S30. Neuroticism and Deception by Situation Variable

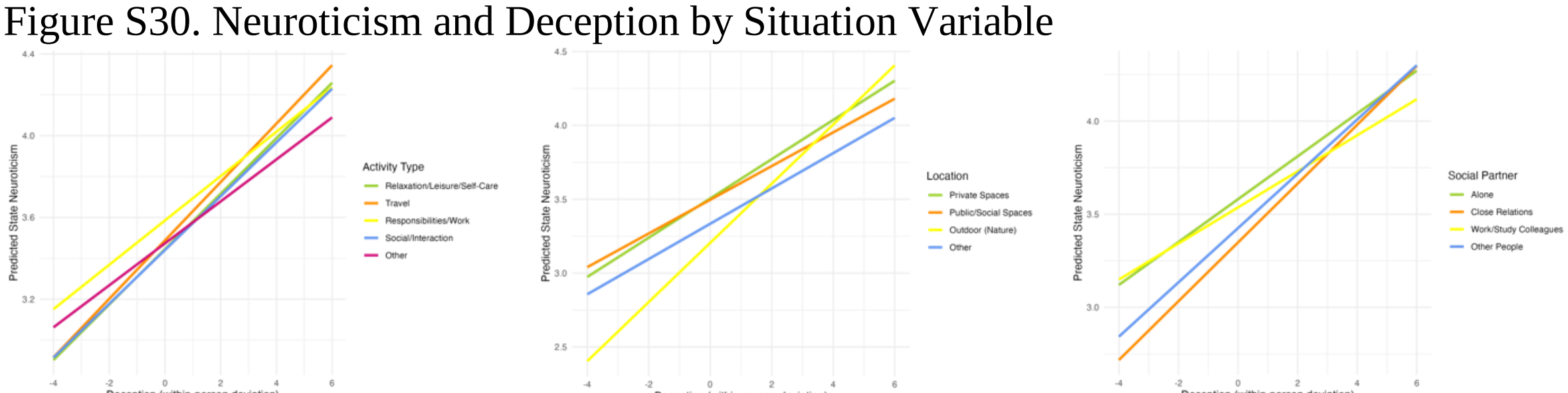

Figure S31. Neuroticism and Duty by Situation Variable

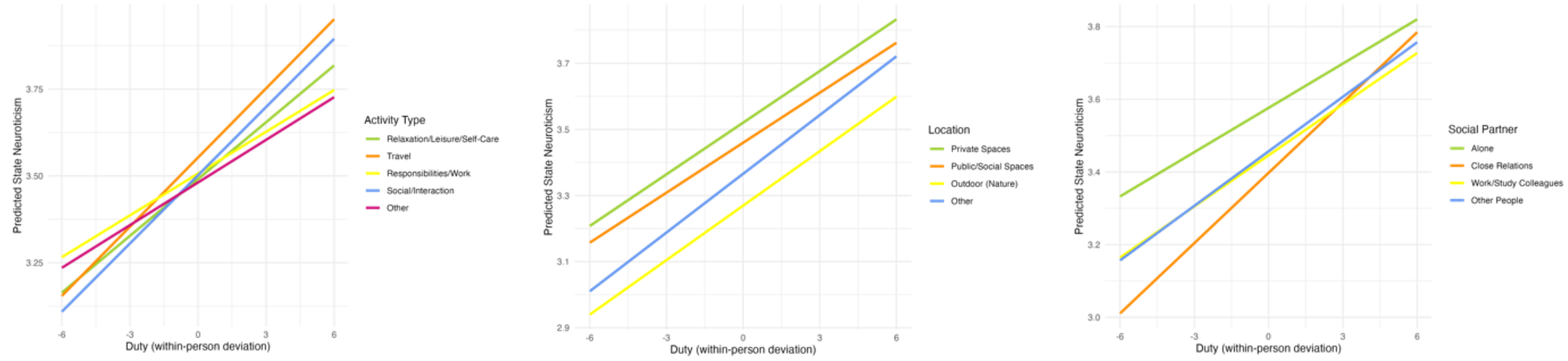

Figure S32. Neuroticism and Intellect by Situation Variable

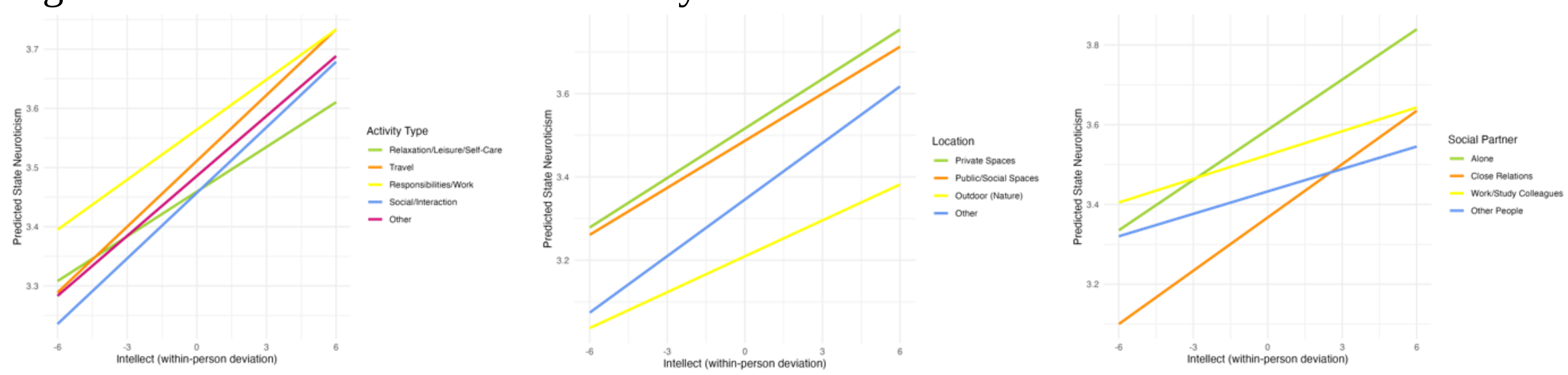

Figure S33. Neuroticism and Mating by Situation Variable

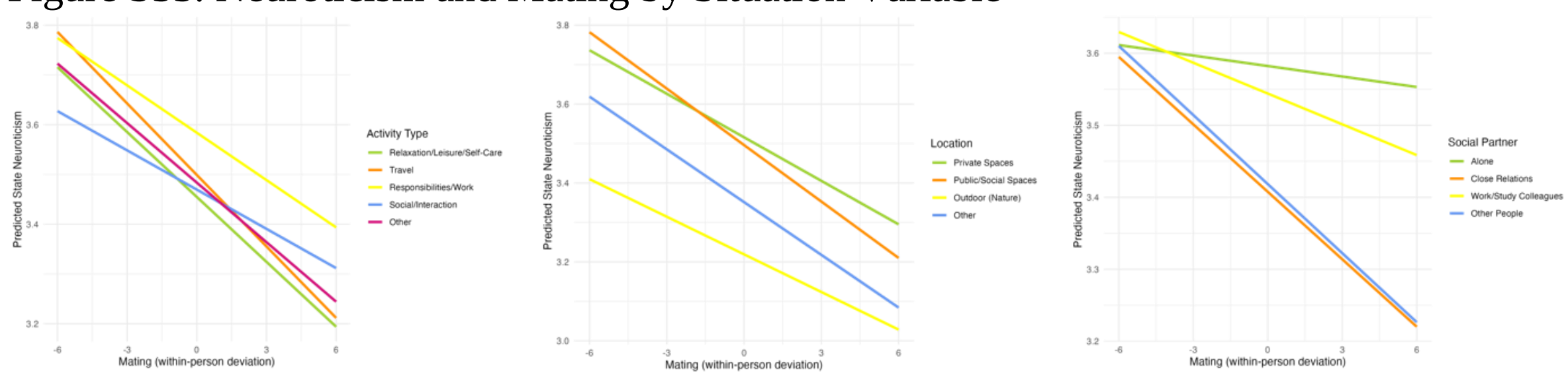

Figure S34. Neuroticism and Negativity by Situation Variable

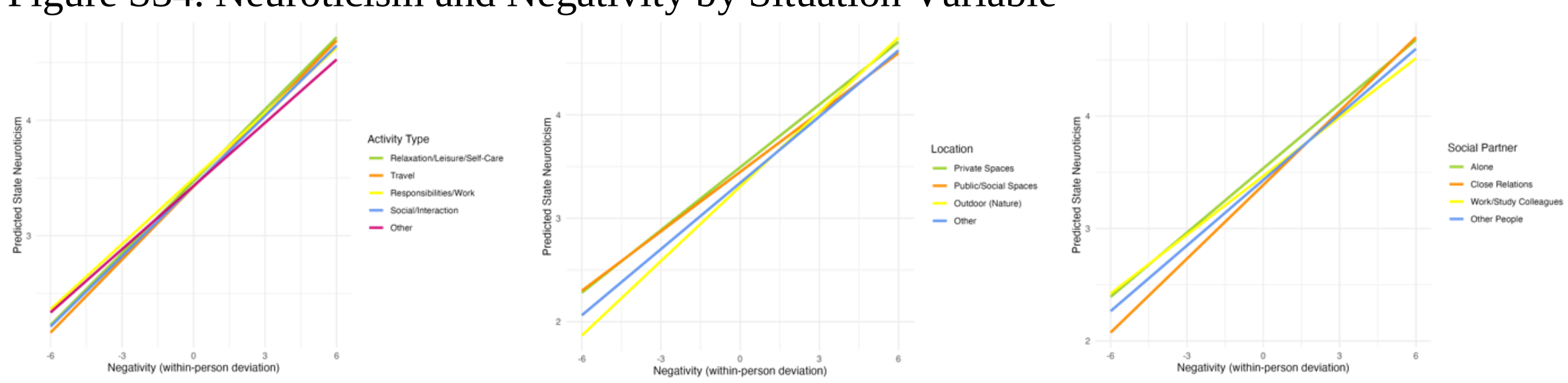

Figure S35. Neuroticism and Positivity by Situation Variable

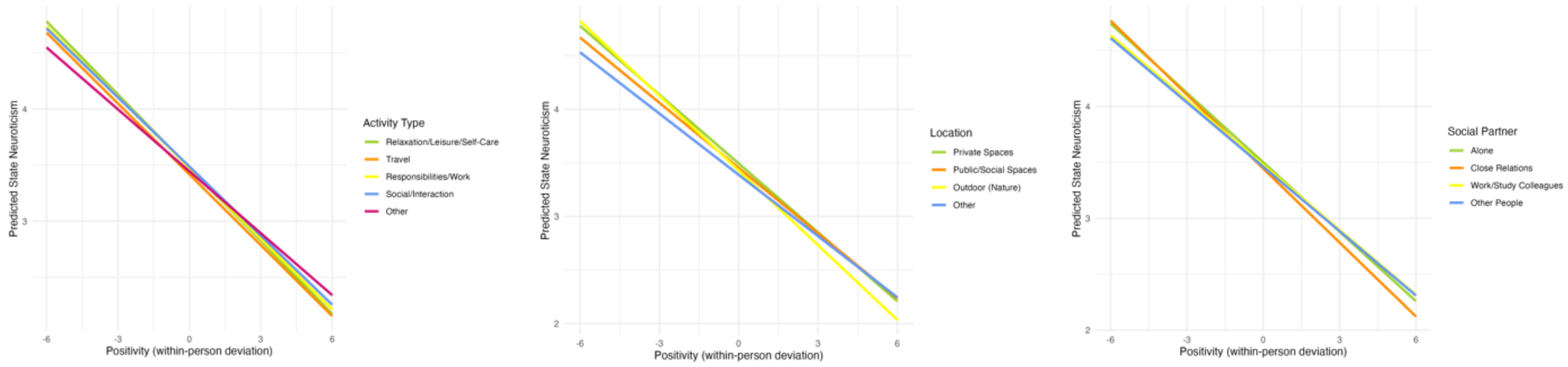

Figure S36. Neuroticism and Sociality by Situation Variable

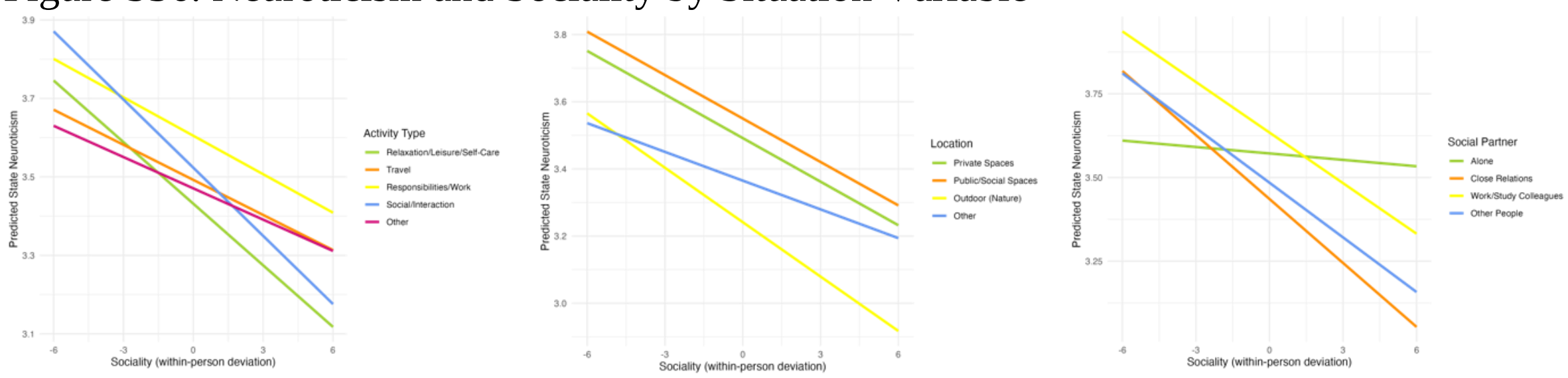

Figure S37. Neuroticism and Typicality by Situation Variable

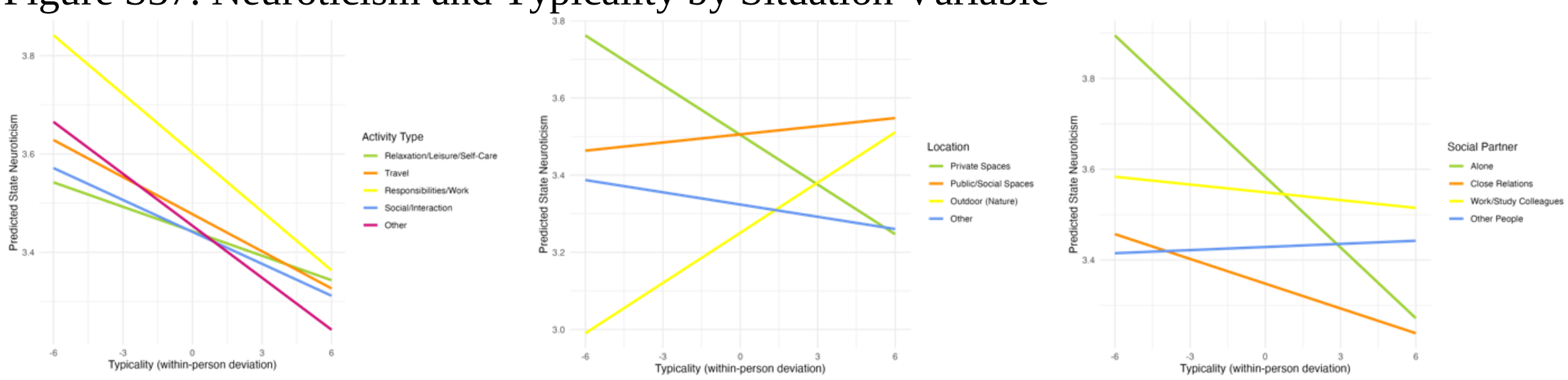

Figure S38. Openness and Adversity by Situation Variable

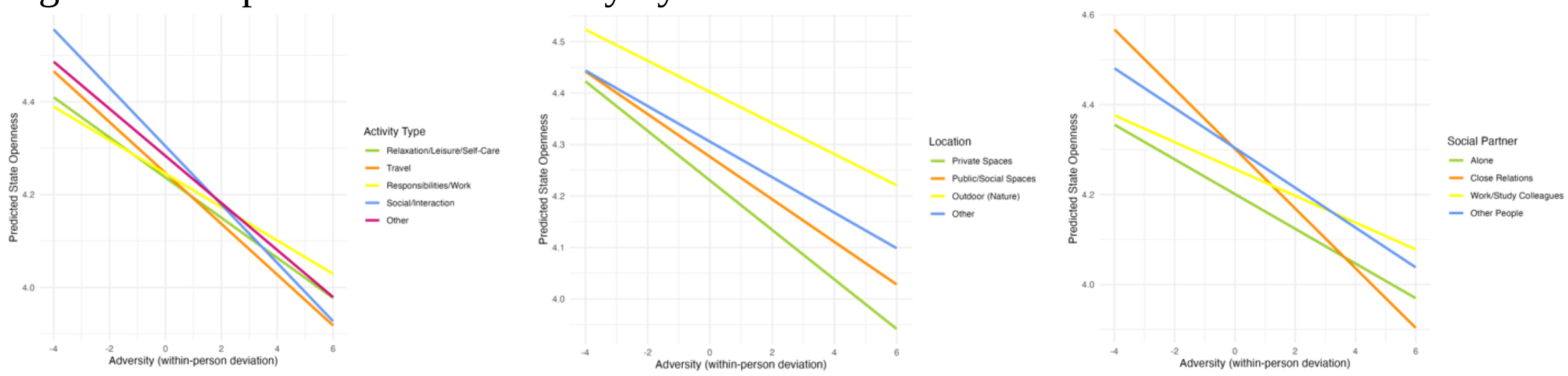

Figure S39. Openness and Deception by Situation Variable

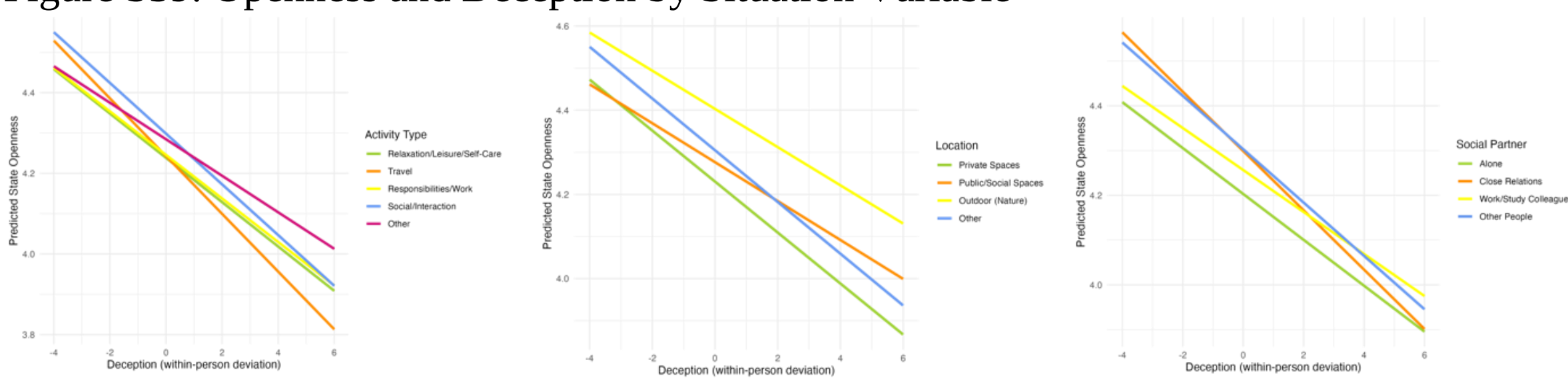

Figure S40. Openness and Duty by Situation Variable

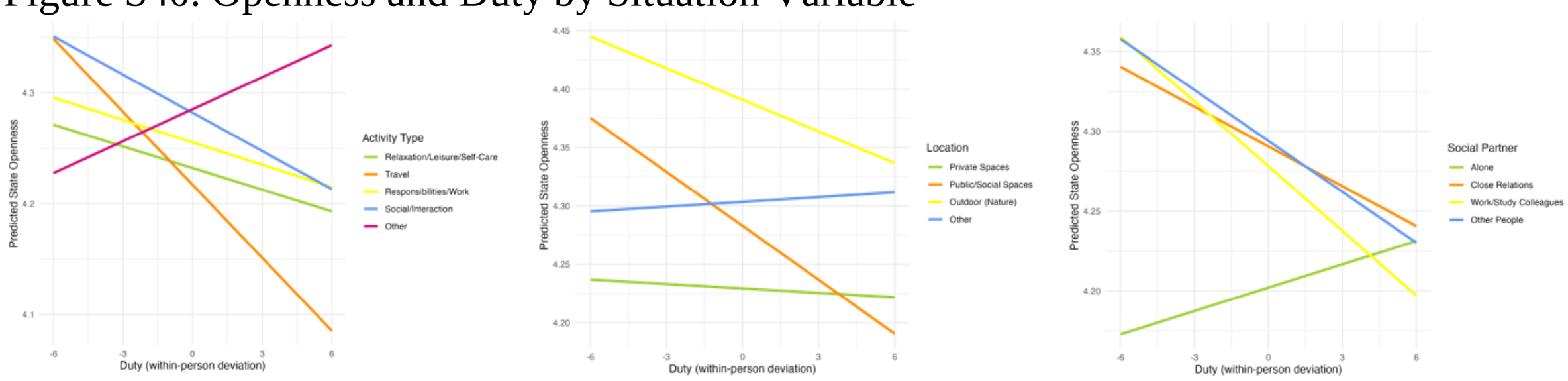

Figure S41. Openness and Intellect by Situation Variable

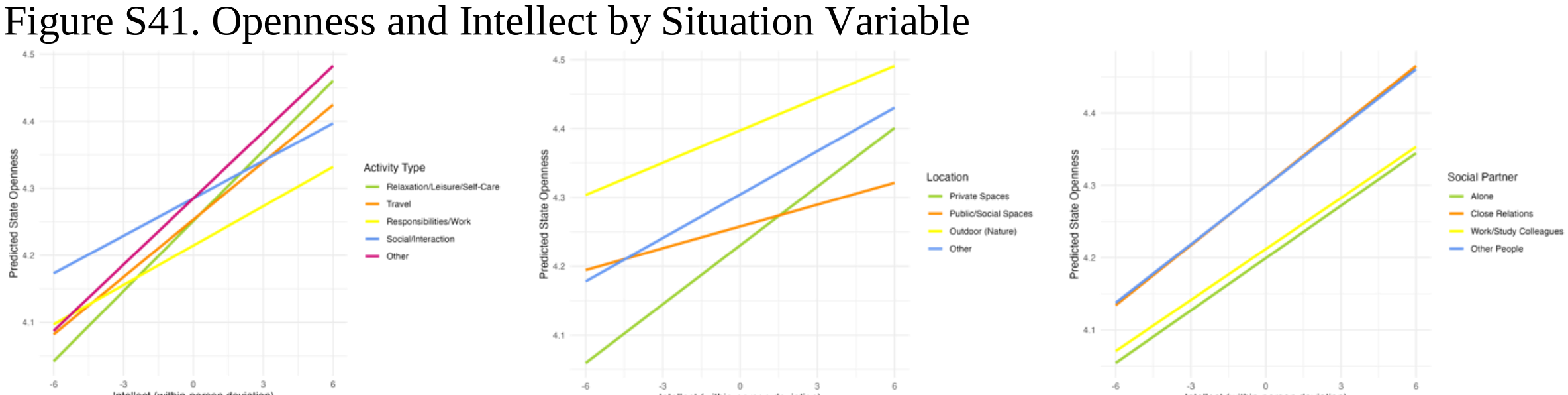

Figure S42. Openness and Mating by Situation Variable

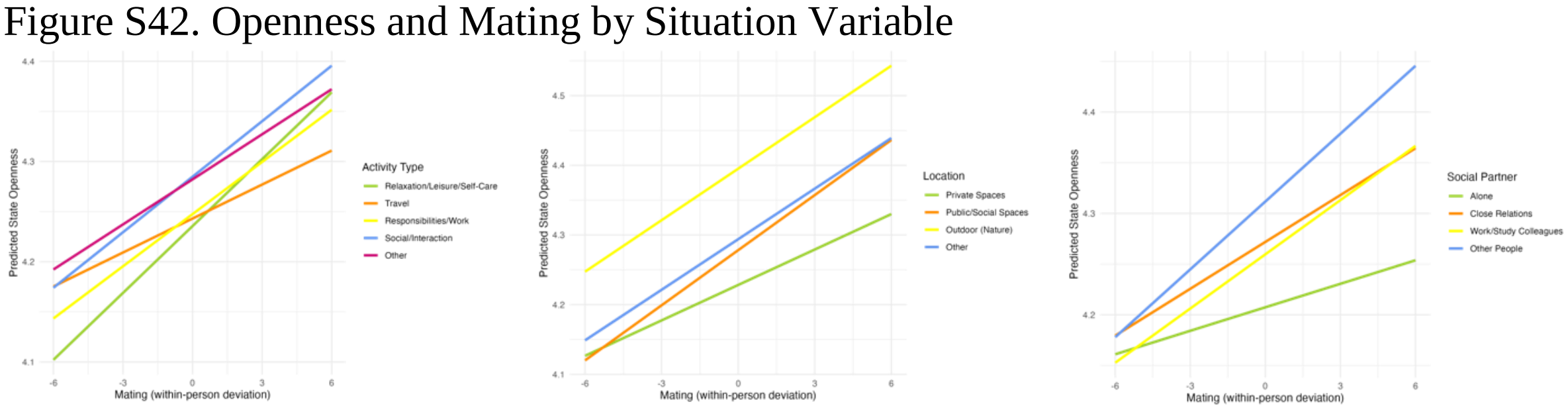

Figure S43. Openness and Negativity by Situation Variable

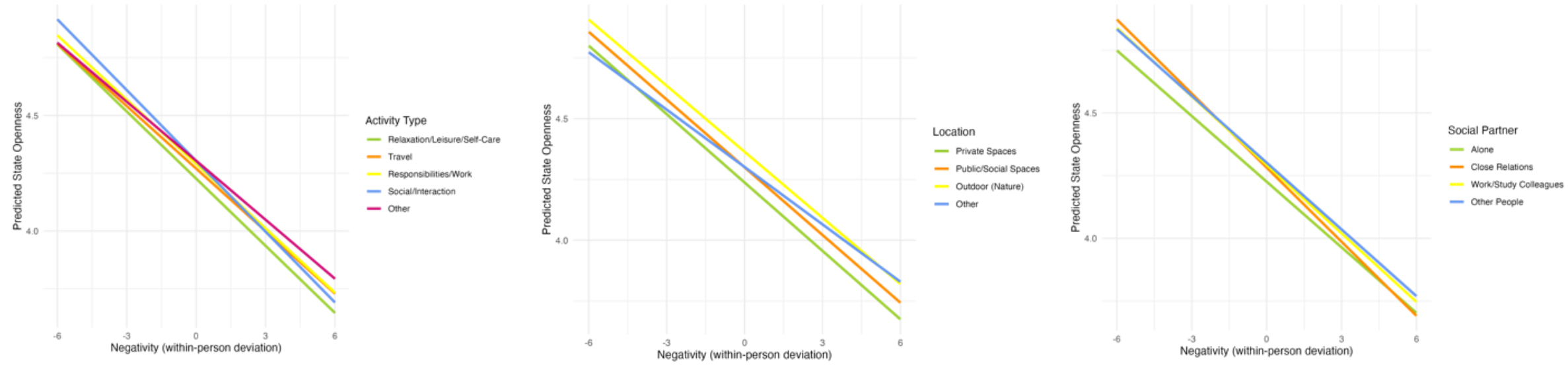

Figure S44. Openness and Positivity by Situation Variable

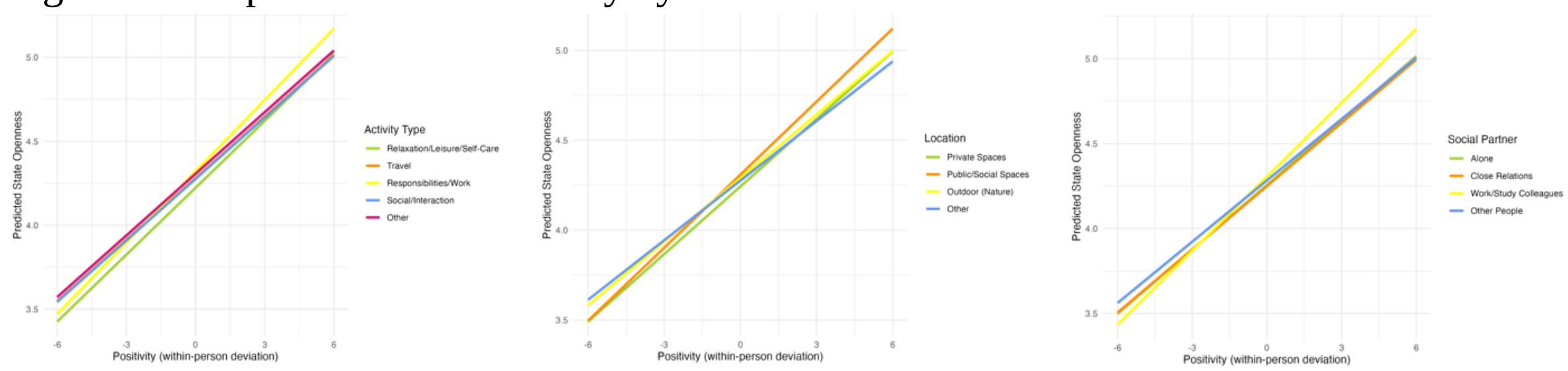

Figure S45. Openness and Sociality by Situation Variable

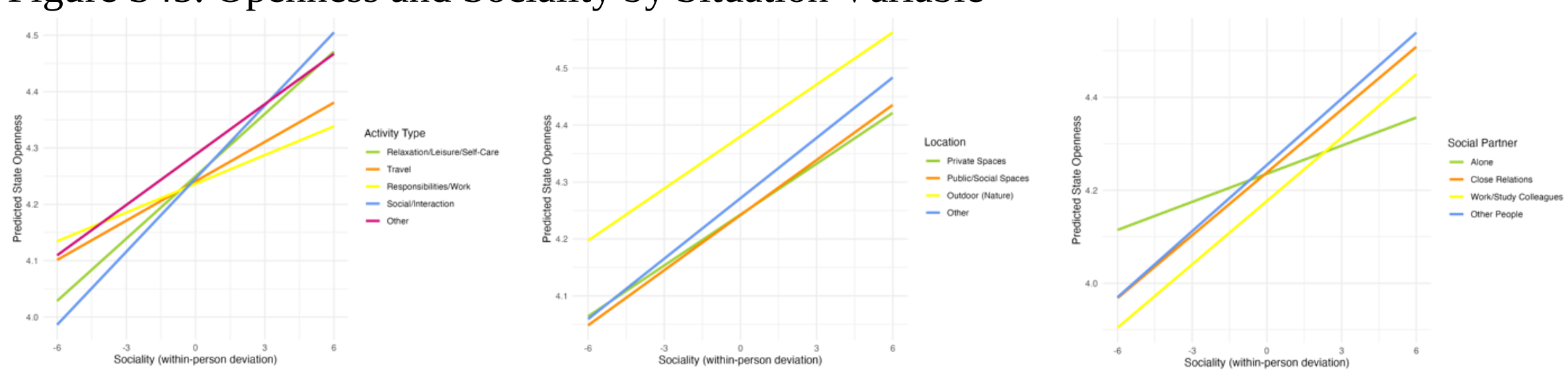

Figure S46. Openness and Typicality by Situation Variable

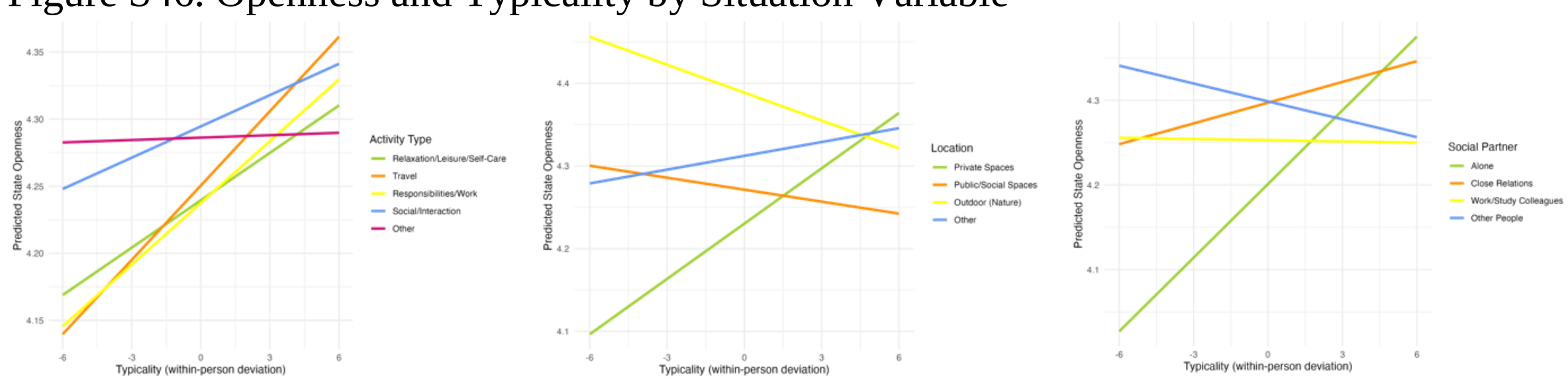

Figure S47

Spaghetti Plots of Extraversion and the DIAMONDS Situation Perceptions in a Laboratory Setting

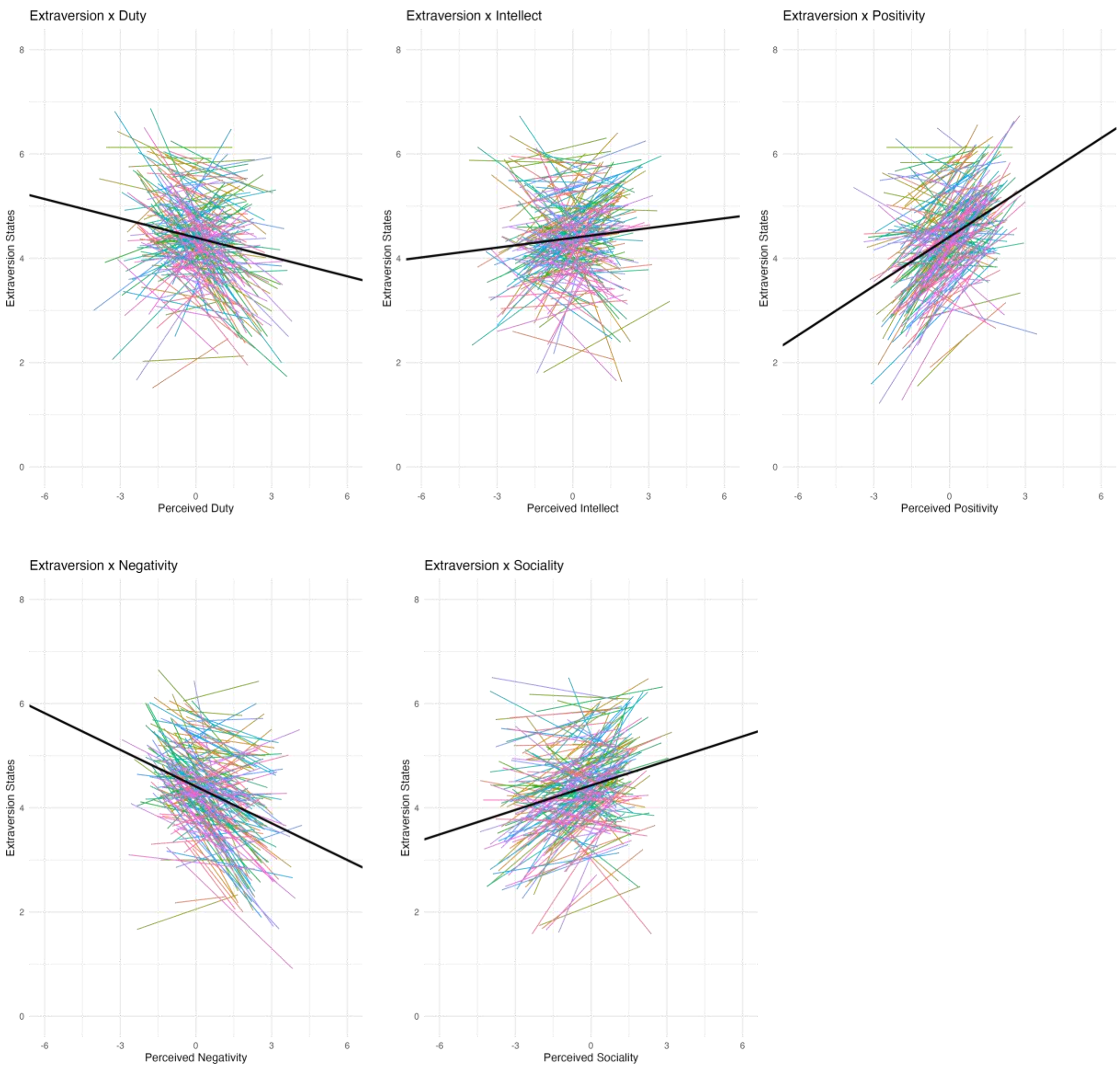

Note. Only models for which Model IV—including random effects—provided the best fit are displayed in the figure.

Figure S48

Spaghetti Plots of Agreeableness and the DIAMONDS Situation Perceptions in a Laboratory Setting

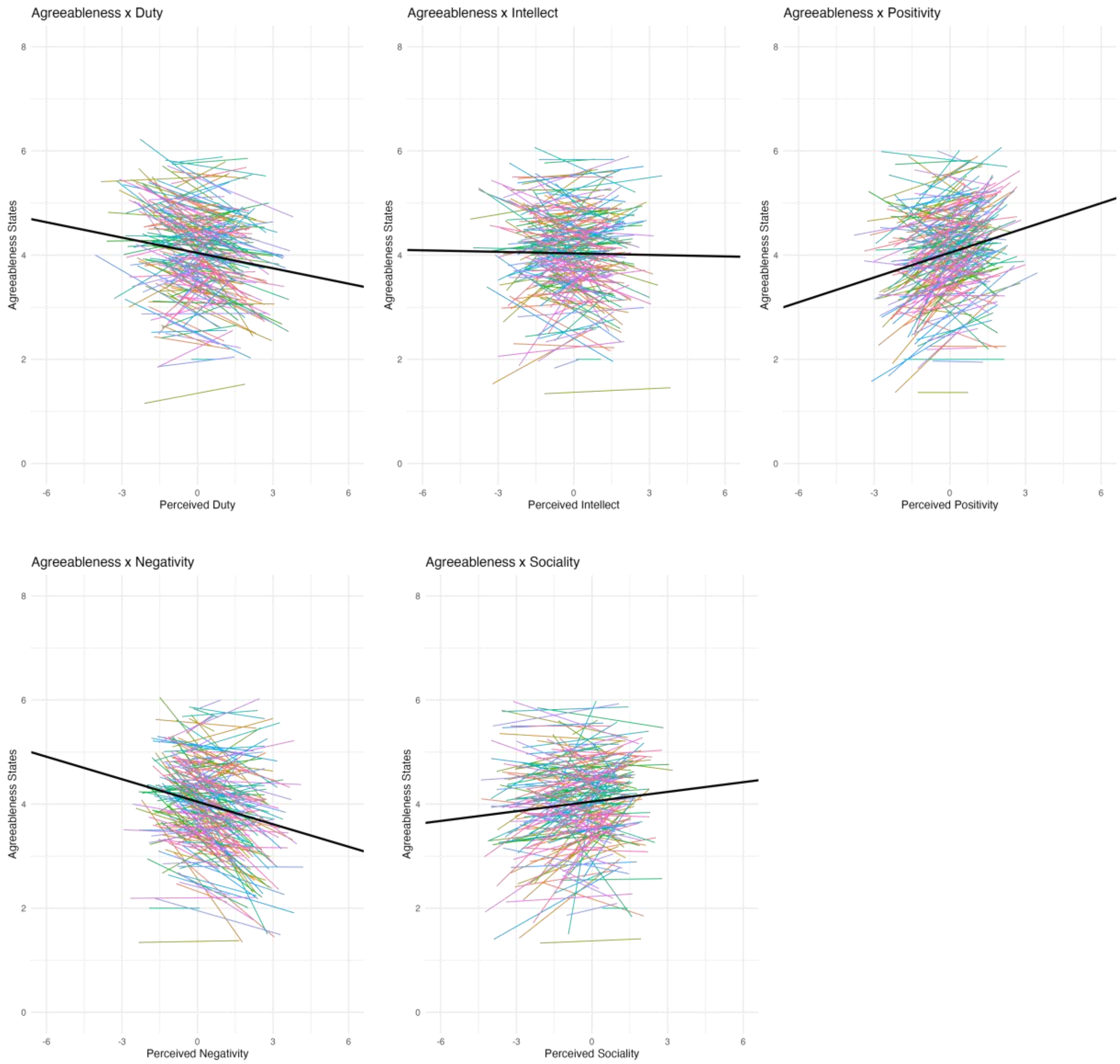

Note. Only models for which Model IV—including random effects—provided the best fit are displayed in the figure.

Figure S49

Spaghetti Plots of Conscientiousness and the DIAMONDS Situation Perceptions in a Laboratory Setting

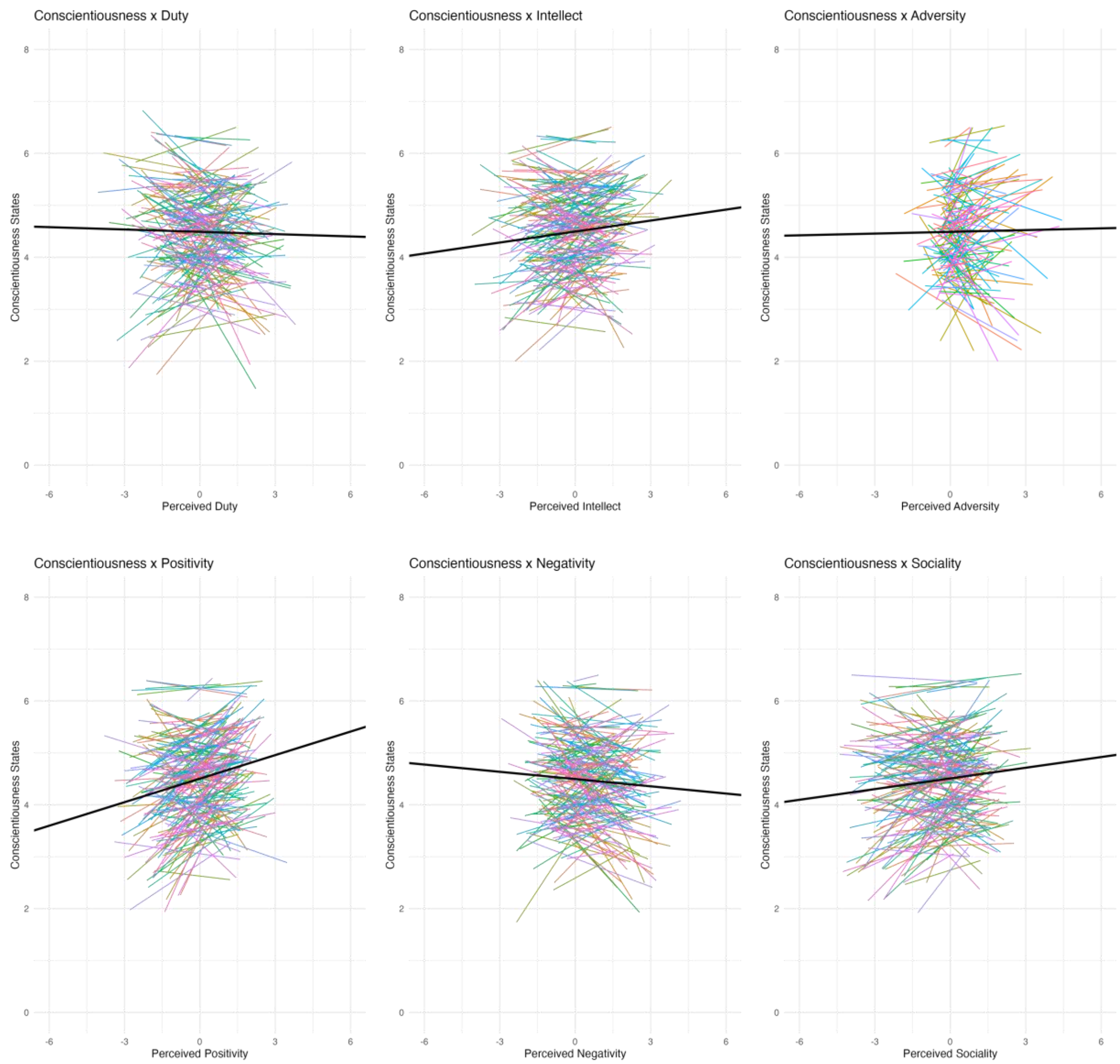

Note. Only models for which Model IV—including random effects—provided the best fit are displayed in the figure.

Figure S50

Spaghetti Plots of Neuroticism and the DIAMONDS Situation Perceptions in a Laboratory Setting

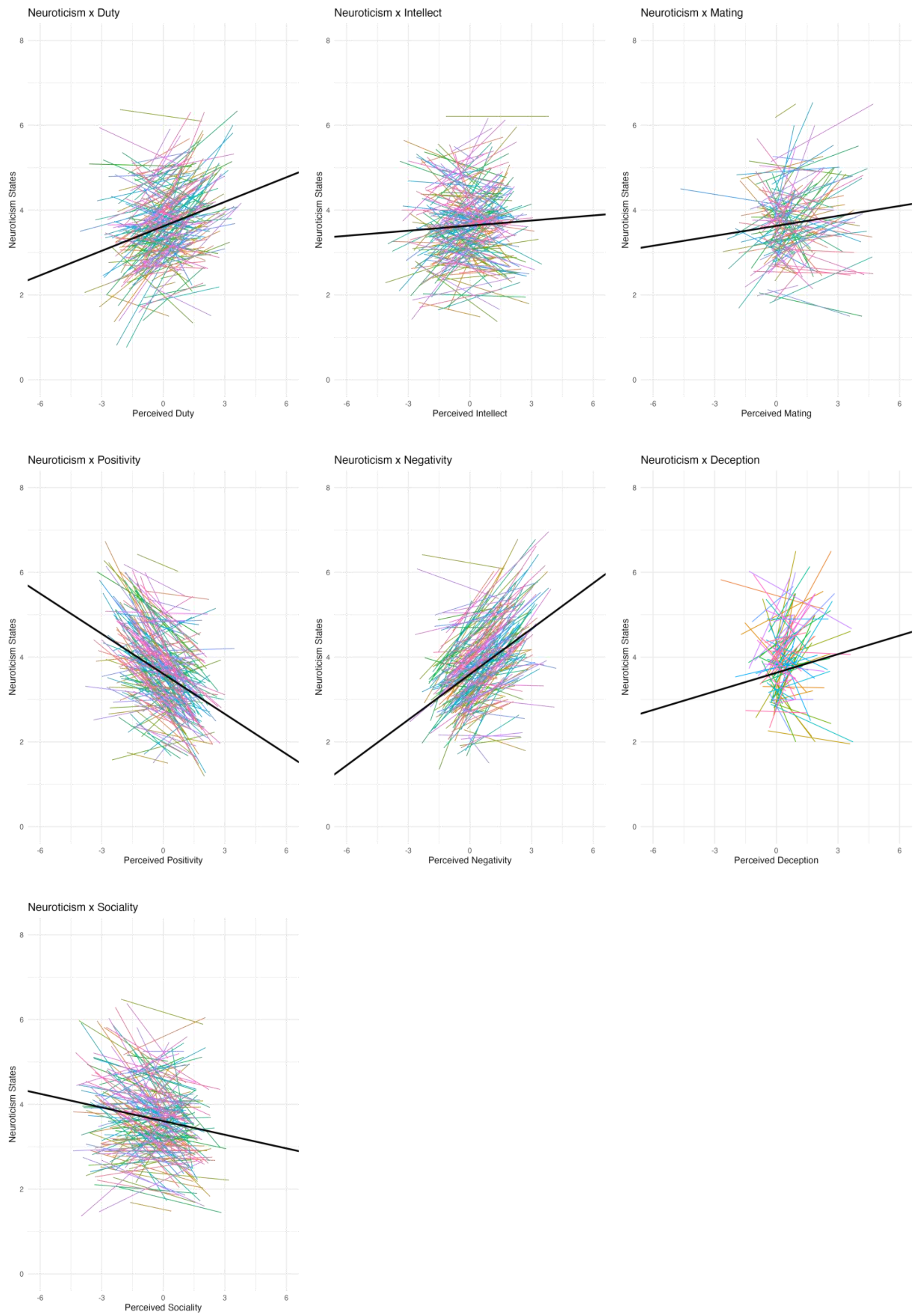

Neuroticism x Duty
Neuroticism States
Perceived Duty
Neuroticism x Intellect
Neuroticism States
Perceived Intellect
Neuroticism x Mating
Neuroticism States
Perceived Mating
Neuroticism x Positivity
Neuroticism States
Perceived Positivity
Neuroticism x Negativity
Neuroticism States
Perceived Negativity
Neuroticism x Deception
Neuroticism States
Perceived Deception
Neuroticism x Sociality
Neuroticism States
Perceived Sociality

Note. Only models for which Model IV—including random effects—provided the best fit are displayed in the figure.

Figure S51

Spaghetti Plots of Openness and the DIAMONDS Situation Perceptions in a Laboratory Setting

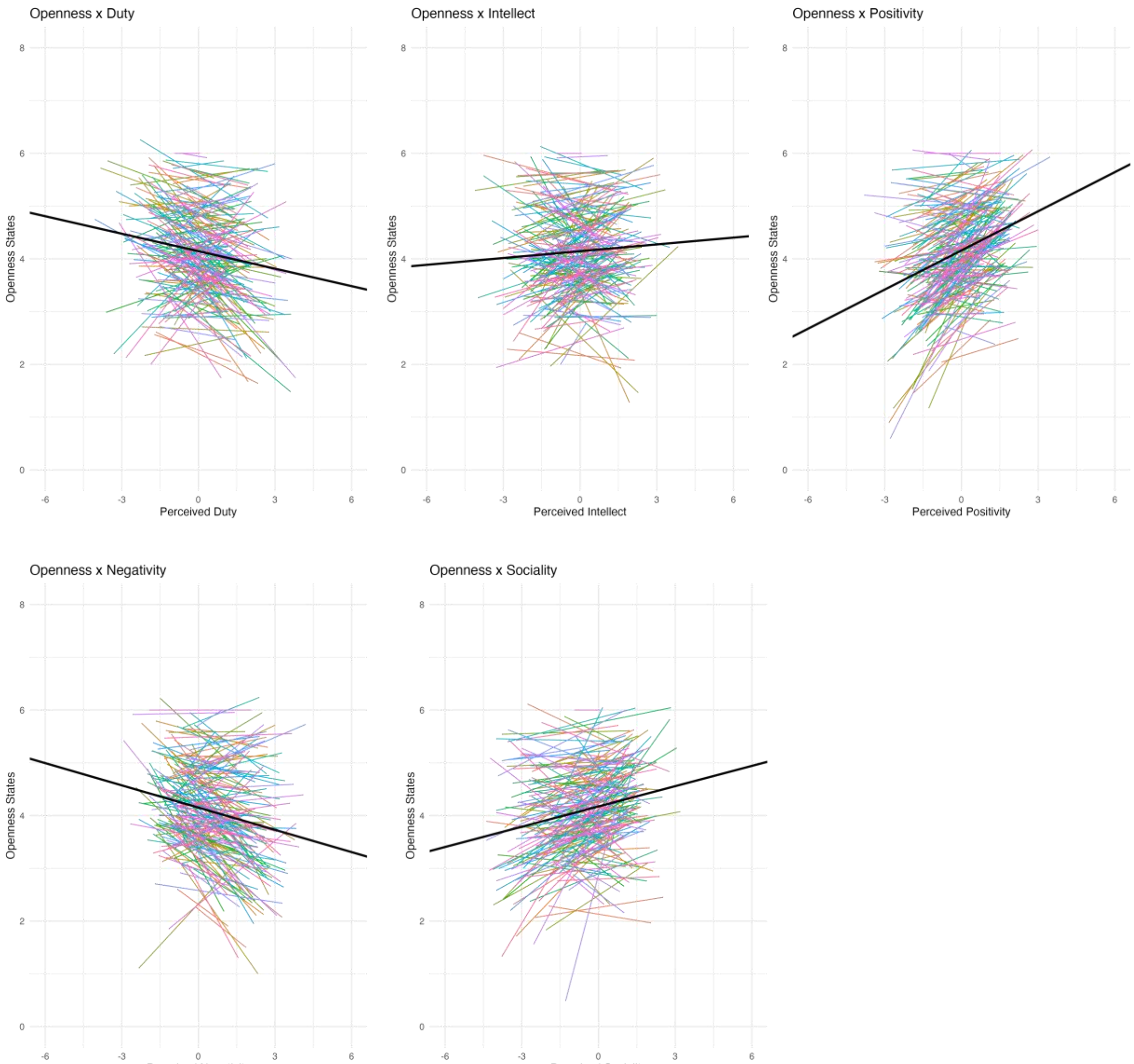

Note. Only models for which Model IV—including random effects—provided the best fit are displayed in the figure.

Figure S52

Spaghetti Plots of Extraversion and the DIAMONDS Situation Perceptions in a Naturalistic Setting

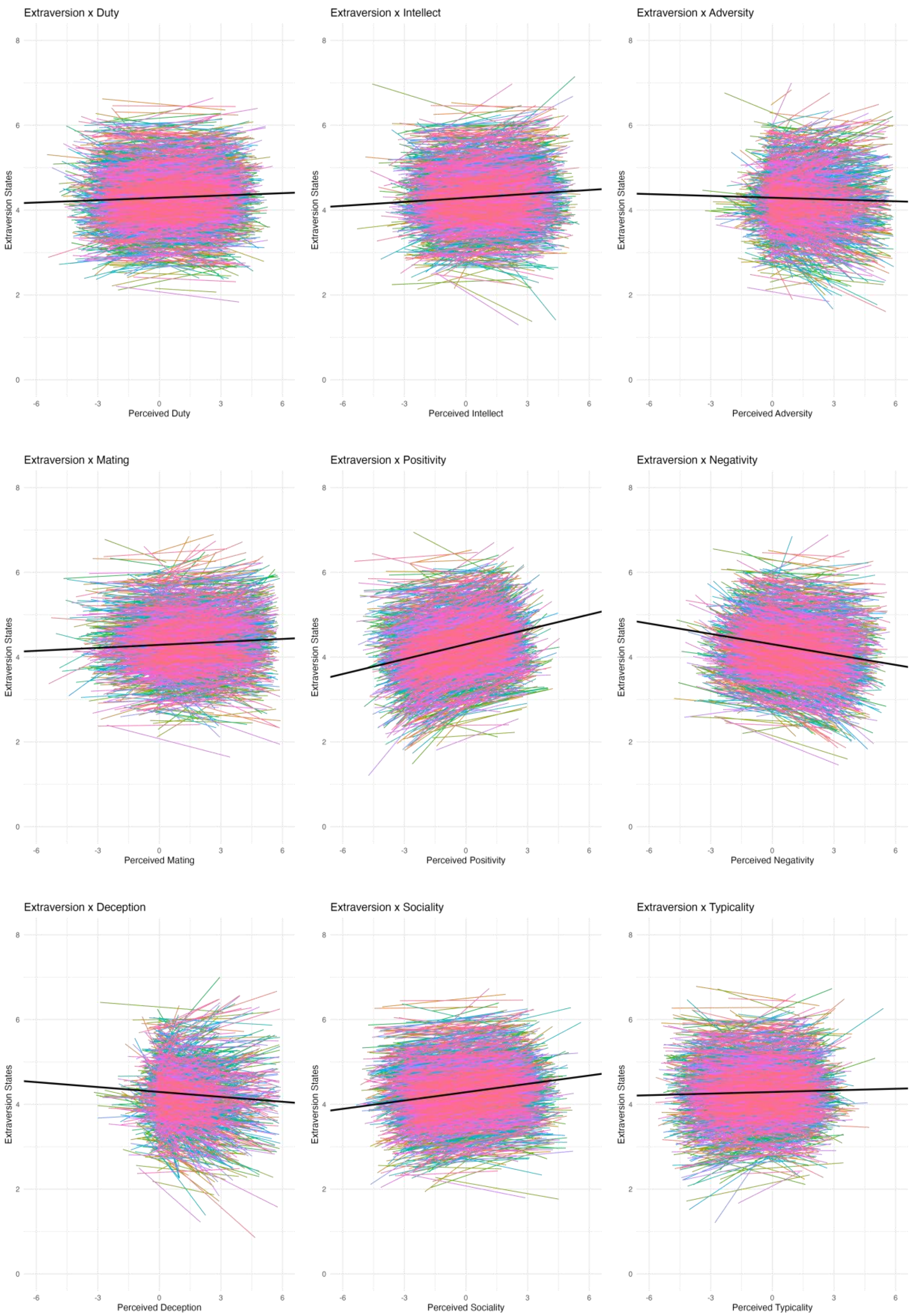
Extraversion x Duty
Extraversion x Intellect
Extraversion x Adversity
Extraversion x Mating
Extraversion x Positivity
Extraversion x Negativity
Extraversion x Deception
Extraversion x Sociality
Extraversion x Typicality
Extraversion States
Perceived Duty
Perceived Intellect
Perceived Adversity
Perceived Mating
Perceived Positivity
Perceived Negativity
Perceived Deception
Perceived Sociality
Perceived Typicality
0
2
4
6
8
-6
-3
0
3
6

Figure S53

Spaghetti Plots of Agreeableness and the DIAMONDS Situation Perceptions in a Naturalistic Setting

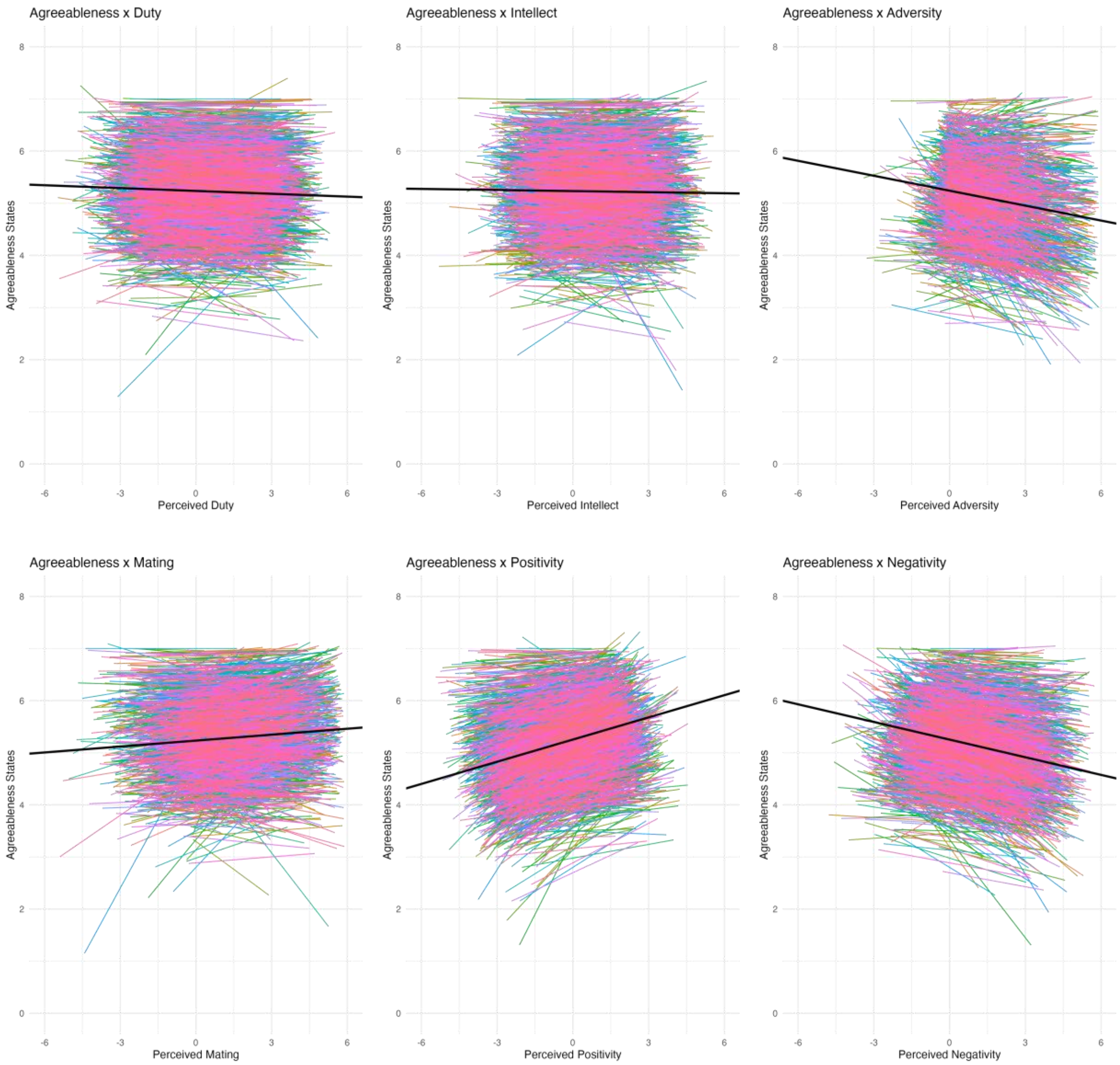

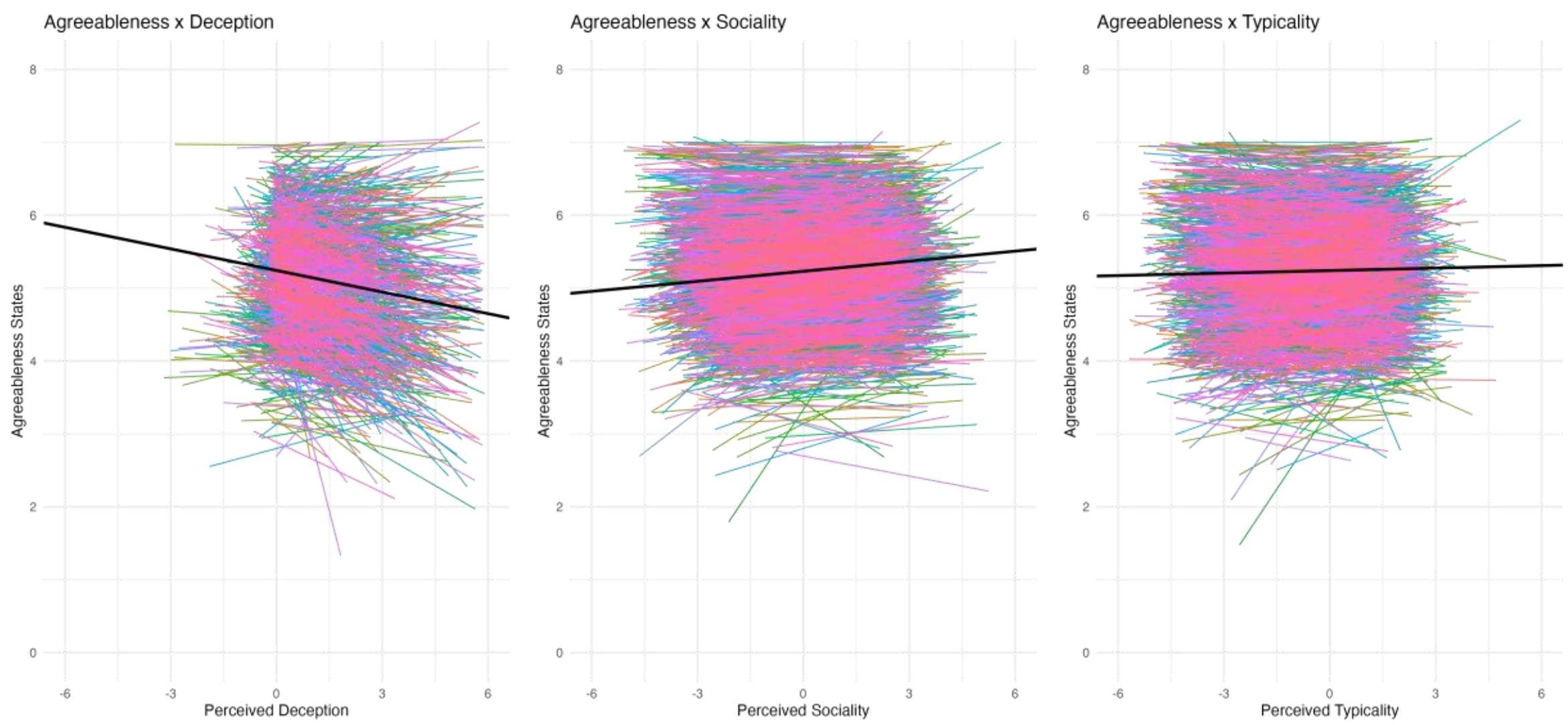

Figure S54

Spaghetti Plots of Conscientiousness and the DIAMONDS Situation Perceptions in a Naturalistic Setting

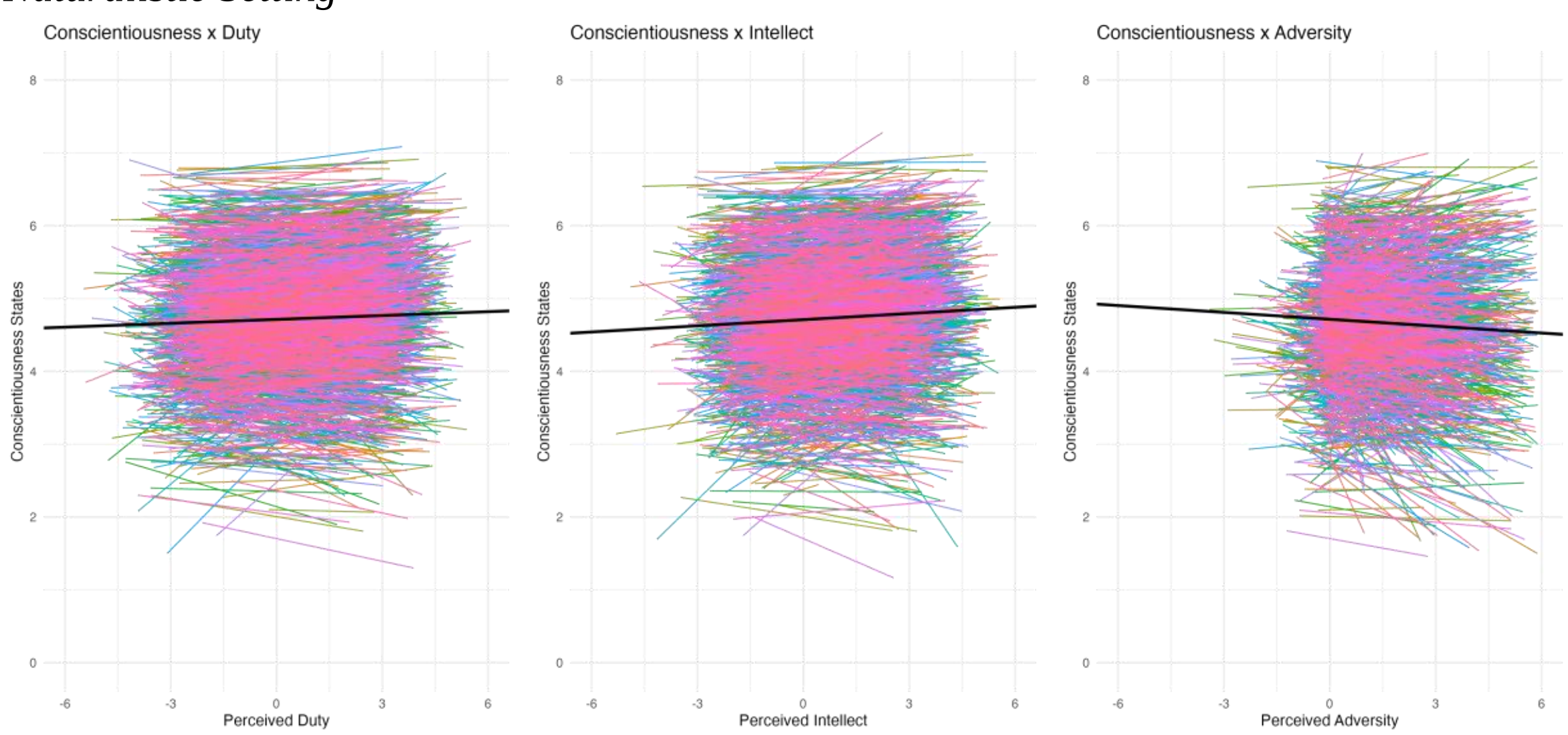

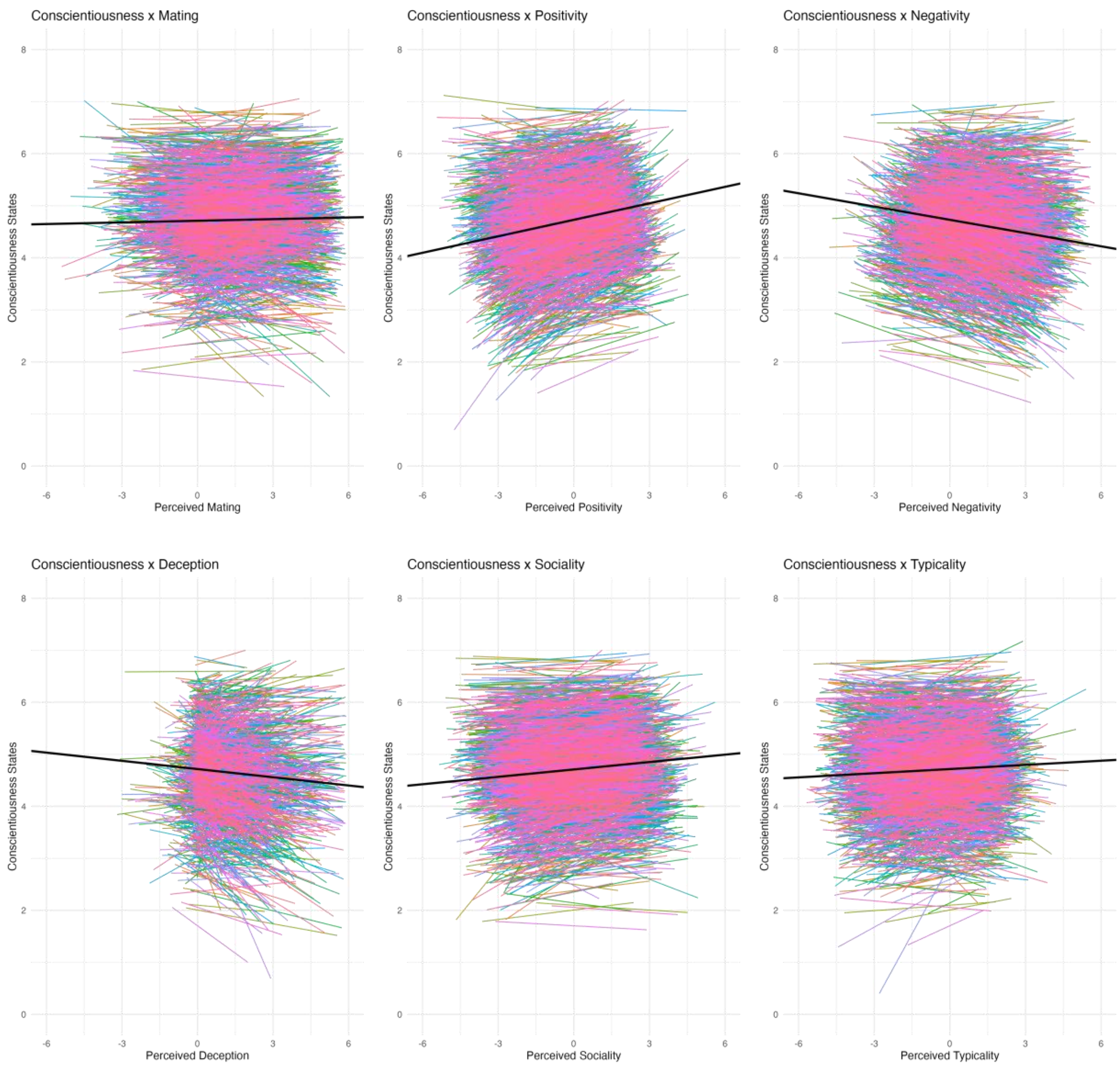

Figure S55

Spaghetti Plots of Neuroticism and the DIAMONDS Situation Perceptions in a Naturalistic Setting

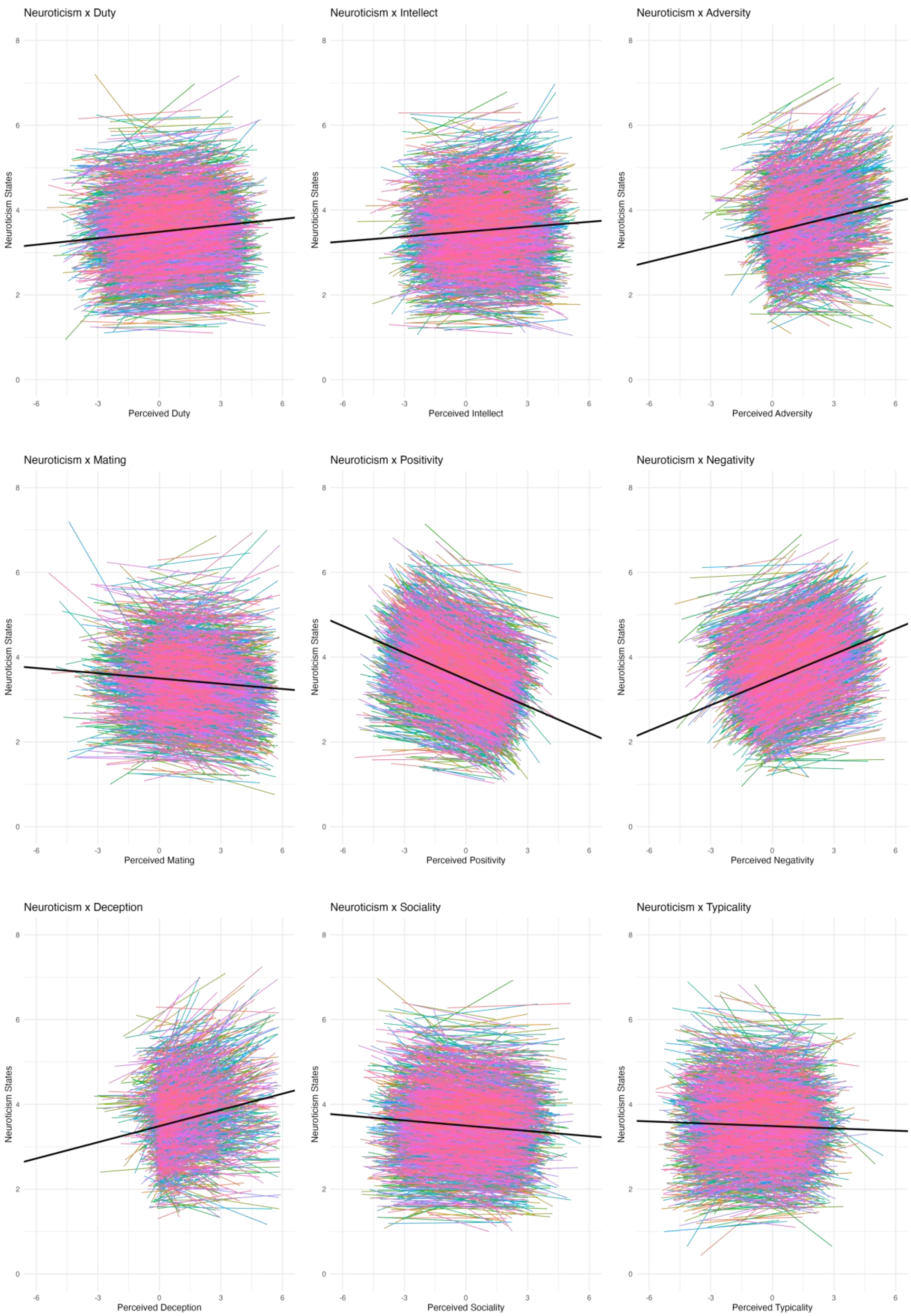
Neuroticism x Duty
Neuroticism States
Perceived Duty
Neuroticism x Intellect
Neuroticism States
Perceived Intellect
Neuroticism x Adversity
Neuroticism States
Perceived Adversity
Neuroticism x Mating
Neuroticism States
Perceived Mating
Neuroticism x Positivity
Neuroticism States
Perceived Positivity
Neuroticism x Negativity
Neuroticism States
Perceived Negativity
Neuroticism x Deception
Neuroticism States
Perceived Deception
Neuroticism x Sociality
Neuroticism States
Perceived Sociality
Neuroticism x Typicality
Neuroticism States
Perceived Typicality

Figure S56

Spaghetti Plots of Openness and the DIAMONDS Situation Perceptions in a Naturalistic Setting

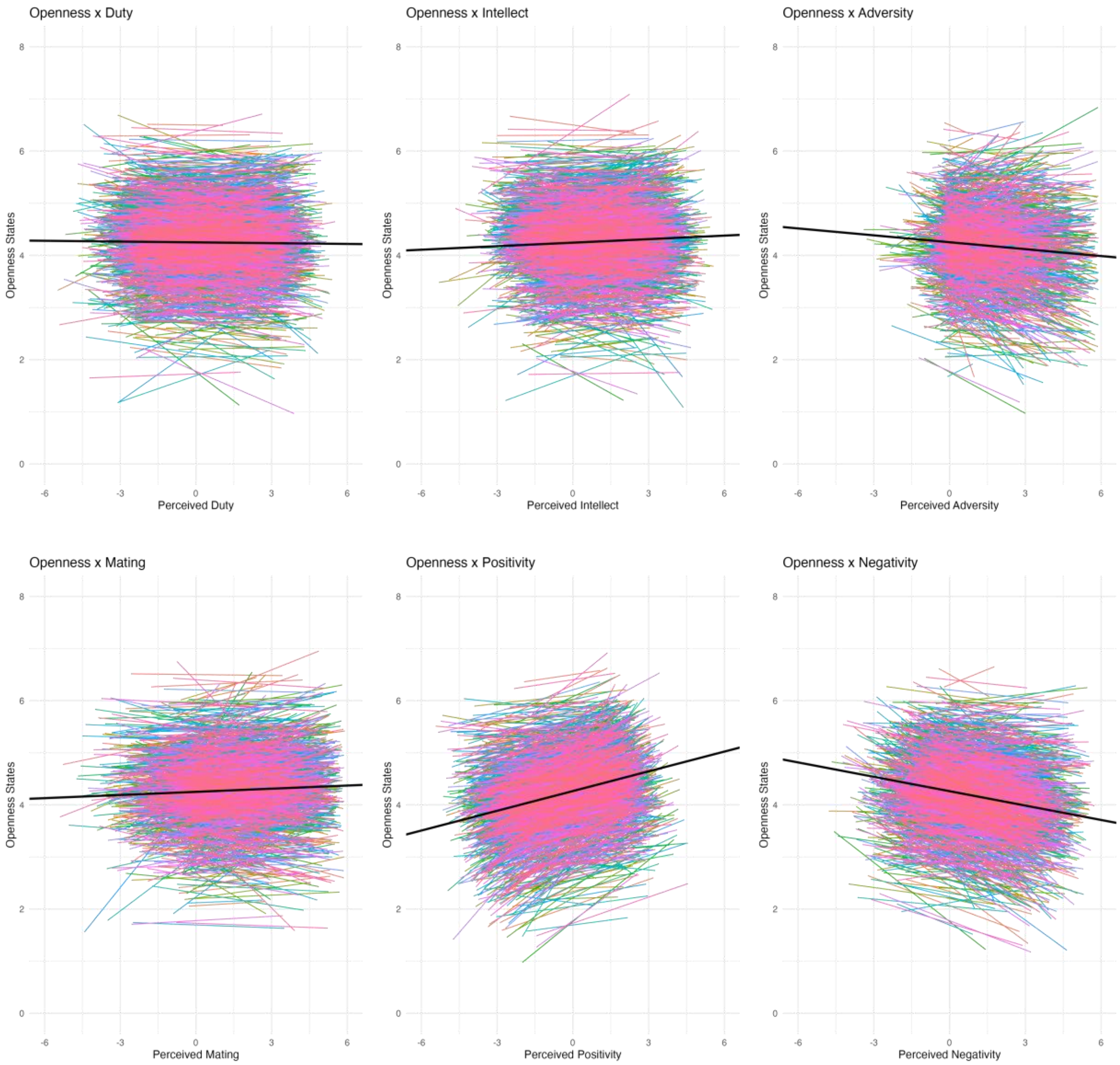

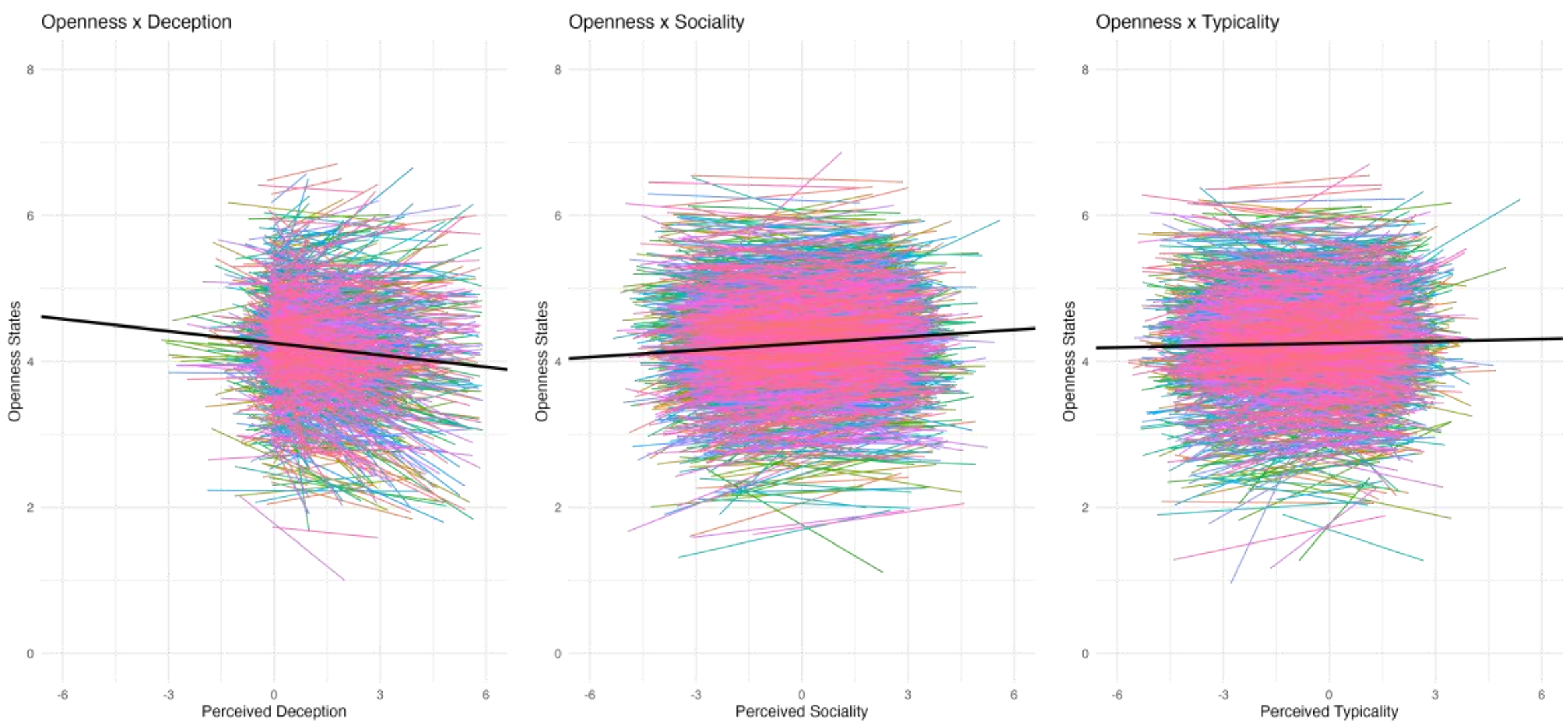

Openness x Deception
Openness x Sociality
Openness x Typicality
Openness States
Perceived Deception
Perceived Sociality
Perceived Typicality
0
2
4
6
8
-6
-3
0
3
6
